# Sustainable Myrcene-Based Elastomers via a Convenient Anionic Polymerization

**DOI:** 10.3390/polym13050838

**Published:** 2021-03-09

**Authors:** David Hermann Lamparelli, Magdalena Maria Kleybolte, Malte Winnacker, Carmine Capacchione

**Affiliations:** 1 Department of Chemistry and Biology “Adolfo Zambelli”, University of Salerno, Giovanni Paolo II Str., 84084 Fisciano, Italy; dlamparelli@unisa.it; 2 WACKER-Chair of Macromolecular Chemistry, Technische Universität München, Lichtenbergstraße 4 Str., 85747 Garching bei München, Germany; magdalena.kleybolte@tum.de; 3 Catalysis Research Center (CRC), Ernst-Otto-Fischer-Straße 1, 85748 Garching bei München, Germany

**Keywords:** myrcene, sodium hydride, anionic polymerization

## Abstract

Soluble heterocomplexes consisting of sodium hydride in combination with trialkylaluminum derivatives have been used as anionic initiating systems at 100 °C in toluene for convenient homo-, co- and ter-polymerization of myrcene with styrene and isoprene. In this way it has been possible to obtain elastomeric materials in a wide range of compositions with interesting thermal profiles and different polymeric architectures by simply modulating the alimentation feed and the (monomers)/(initiator systems) ratio. Especially, a complete study of the myrcene-styrene copolymers (PMS) was carried out, highlighting their tapered microstructures with high molecular weights (up to 159.8 KDa) and a single glass transition temperature. For PMS copolymer reactivity ratios, r_myr_ = 0.12 ± 0.003 and r_sty_ = 3.18 ± 0.65 and r_myr_ = 0.10 ± 0.004 and r_sty_ = 3.32 ± 0.68 were determined according to the Kelen–Tudos (KT) and extended Kelen–Tudos (exKT) methods, respectively. Finally, this study showed an easy accessible approach for the production of various elastomers by anionic copolymerization of renewable terpenes, such as myrcene, with commodities.

## 1. Introduction

In the last two decades, on the one side under globalization pressures there has been an extraordinary planetary expansion of plastics and rubbers manufactured for human use (359 million tons in 2018) [1]; on the other side concerns have grown about environmental problems, dwindling petrochemical reserves and climatic changes, also due to the accumulation of CO_2_ in the atmosphere.

Within this scenario, the scientific community and chemical industry have directed their efforts toward more sustainable processes [2] by using feedstock derived from renewable resources as alternatives to petroleum-based products in order to reduce our dependence on fossil raw materials and their adverse effects on the ecosystems [3,4]. Fortunately, nature offers many different compounds, which are excellent building blocks for constructing advanced materials: polysaccharides and their derivatives, lignin, suberin, vegetable oils, tannins, monomers originating from sugars, furan derivatives, lactic acid and fatty acid, which are just a few examples [5,6]. In this context, the large family of terpenes represents a natural, potentially important source of unsaturated hydrocarbons, useful in producing polymers [7,8,9,10,11]. Terpenes are ubiquitous molecules, characterized by the presence of one or more double bonds, found in many plants (mainly conifers), fungi and even some insect, which have both linear and cyclic structures and share 2-methyl-1,4-butadiene (isoprene) (C5) as an elementary unit [12]. Among these compounds, β-myrcene (M)—due to the structural similarity to isoprene (I) and butadiene (B) and large availability—has recently aroused a growing attention as building block for the synthesis of a vast range of polymers, including the elastomer synthesis. Really, the first works on M date back to the 40s of the last century and concern an emulsion polymerization [13] and a study to avoid the deterioration of the monomer during storage due to its reactivity [14]. M, a colorless liquid belongs to the class of acyclic monoterpenes (C10), contains three double bonds in its structure, bearing a conjugated diene moiety. Although it is naturally produced by various plants, M is industrially generated by pyrolysis of β-pinene and new developments on its production from microbial synthesis via metabolically engineered *Escherichia coli* were also investigated [15,16].

Recently, homopolymerizations of M and its copolymerization with various comonomers, such as conjugated dienes [17,18,19,20], styrene (S) [21,22,23,24] and derivatives [25], ethylene and propylene [26], methacrylates [27,28], itaconic acid [29], and lactide [30] by coordinative mechanism [31,32], free [23,33] and controlled radical polymerizations [34,35,36], cationic [37] and anionic [38,39,40] polymerization methods have been reported. Very recently, a temperature-controlled one-pot anionic approach for the preparation of diblock copolymers consisting of PS and PM blocks has been described [41]. It has also been reported the synthesis of bifunctional (hydroxyl, furan and pyridine group derivatives) monomers dienes obtained from M and their controlled copolymerization with isoprene via catalytic insertion mechanism [42]. Previously, Cawse and co-workers used hydroxyl-functionalized polymyrcene to modify a polyurethane network leading to improved impact properties and stress–strain behavior [43].

Depending on the polymerization conditions, the polymyrcene can consist of 1,4 *cis*/*trans*, 1,2 and 3,4 units. However, the most promising application in the large-scale use of these polymers as elastomers has been still lacking. Elastomeric materials play a fundamental role in human life, being used in countless applications ranging from tire to spacecraft seal [44,45].

In the rubber industry, excluding natural rubber (NR), polybutadiene (BR) and styrene-butadiene rubber (SBR) are the two main products [45,46], these latter are produced on a large scale from emulsion (E-SBR) [47,48] and solution (S-SBR) [49] polymerization. S-SBR is synthesized by an anionic polymerization process, which is initiated by organolithiums compounds, such as sec-butyl lithium (sec-BuLi) or *n*-butyl lithium (*n*-BuLi), soluble in common organic solvents. Indeed, anionic polymerization is still one of the most powerful synthetic tools for the preparation of well-defined polymers, allowing the control of the molecular architecture (molecular weight, molecular weight distribution, composition and microstructure) [50]. Carbanions (or oxanions) with metallic counterions are mostly the active centers, highly sensitive to oxygen, moisture and protic impurities in the chain propagation step.

Alkali metal hydrides (Li, Na, K) can be considered a viable alternative to organolithium compounds as anionic polymerization initiator but have been barely investigated because their low solubility in organic solvents. Notably, the addition of Lewis acid (R_3_Al, R_3_B, R_2_Zn, or R_2_Mg), in particular of trialkylaluminium (Et_3_Al and i-Bu_3_Al) to LiH, NaH and KH, allows for their solubilization in non-polar solvents by formation of bimetallic complexes [51,52,53,54].

They are compounds quickly available and stable under inert atmosphere in which one or more hydrogen centers have nucleophilic, basic or reducing properties, commonly used as reducing agents in organic synthesis. LiH, NaH and KH have been employed to very limited cases in polymerization [55] and only in recent years systems based on their association with alkyl, alkoxide and hydride derivatives of Al, Mg and Zn in the RAP of styrene and dienes have been explored [56,57,58]. Deffieux et al. reported that triisobutylaluminium (TIBA) and sodium hydride form soluble heterocomplexes (*i*-Bu_3_Al/NaH) usable as initiating systems for the retarded anionic polymerization (RAP) of S at high temperature in concentrated monomer conditions [56].

**Figure 1:**
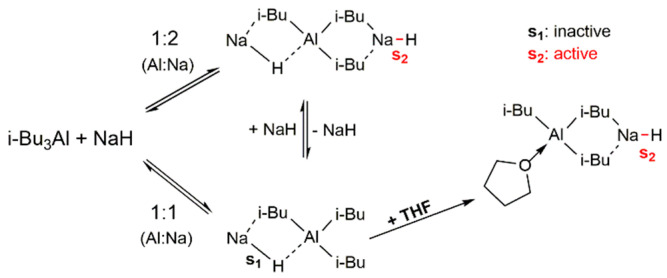
Formation of 1:2 and 1:1 Al/Na heterocomplexes of *i*-Bu_3_Al/NaH system and influence of THF on an inactive 1:1 complex, as suggested by Deffieux et al. [58].

The best performances were obtained at ratios 0.7 < Al:Na < 1, since for stoichiometric proportion or higher than 1 the system was inactive. As depicted in Figure 1, at ratios Al:Na ≥ 1, only the 1:1 complex with an inactive site (S_1_) was formed. Instead, in the presence of a slight excess of NaH with respect to TIBA, in addition to the 1:1 complex, the formation of the 1:2 complex with an active site was postulated. Furthermore, the addition of tetrahydrofuran (THF) as a second polar additive increased the reactivity of the system and could lead to a mixture of complexes in which the formation of an active Na–H bond (S_2_) was favored [54,57].

In the present paper, the anionic homo-, co- and ter-polymerization of M, S and I by sodium hydride in combination with TIBA, as anionic initiator heterocomplexes, in highly concentrated monomer media at 100 °C were investigated. These initiating systems can represent a very useful and economical approach for the copolymerization of M with commodities such as S and I, enlarging the armory of techniques that allow the synthesis of more sustainable terpene-based elastomers.

## 2. Materials and Methods

### 2.1. Materials

All chemicals were stored and handled under an argon atmosphere using standard Schlenk techniques or an MBraun glovebox.

All glassware was stored overnight in an oven at 130 °C prior to use. β-myrcene (≥95%), isoprene and styrene (≥99%) were all purchased from Sigma-Aldrich (Steinheim, Germany) and were purified by overnight stirring over calcium hydride and distillation under reduced pressure. Monomers were stored on molecular sieves at 4 °C and before each set of experiments the amount of water was measured with a Mettler Toledo KF Coulometer DL36 Karl Fischer (Greifensee, Switzerland) instrument (allowed max. 15 ppm H_2_O). Toluene (Sigma-Aldrich) and tetrahydrofuran (Sigma Aldrich) (THF) as solvents dried were obtained with an MBraun MBSPS-800 (Garching, Germany) solvent purification system and were deoxygenized by three freeze-pump-thaw cycles, before storing on molecular sieves. Sodium hydride was stored under argon atmosphere in a drybox. Triisobutylaluminum (Al(i-Bu)_3_, Sigma-Aldrich) (TIBA) was used as received.

### 2.2. Preparation of NaH/i-Bu_3_Al Initiator System

Under argon, NaH (0.096 g, 4 × 10^−3^ mol) was placed in a glass flask before adding 50 mL of dry and degassed toluene. Then, 3 mL of TIBA solution (25% wt in toluene) was added with a syringe to reach the ratio of Al/Na = 0.8. The final solution was let under vigorous stirring at 50 °C, until NaH was partially solubilized (4 h).

### 2.3. Typical Polymerization Procedure

Polymerizations were carried out under argon at 100 °C in a glass flask for 72 h. 88 μL of *i*-Bu_3_Al/NaH solutions (ratio Al/Na = 0.8) and 25 μL of THF were added with a syringe to dry and degassed toluene (0.5 mL). Finally, 0.5 mL of myrcene was added to reaction mixture and the system was maintained under stirring after equilibration at the desired temperature. The polymerization was stopped by adding of methanol containing a little amount of hydrochloric acid and 2,6-bis(1,1-dimethylethyl)-4-methylphenol (BHT) as stabilizing agent. The polymer was washed and dried under vacuum until constant weight. 230 mg polymer (58% yield, Mw = 50.2 KDa, Đ = 1.7).

### 2.4. Typical Co-polymerization Procedure

Co-Polymerizations were carried out under argon at 100 °C in glass flasks for 72 h. 30 μL of *i*-Bu3Al/NaH solutions (ratio Al/Na = 0.8) and 9 μL of THF were added with a syringe to dry and degassed toluene (0.5 mL). Finally, 0.28 mL of myrcene and 0.19 mL of styrene was sequentially added to reaction mixture and the system was maintained under stirring after equilibration at the desired temperature. The co-polymerization was stopped by adding of methanol containing a little amount of hydrochloric acid and 2,6-bis(1,1-dimethylethyl)-4-methylphenol (BHT) as stabilizing agent. The co-polymer was washed and dried under vacuum until constant weight. 295 mg polymer (75% yield, Mw = 151.2, Đ = 2.1).

### 2.5. Preparation of Diblock Copolymer

In a glass flask equipped with magnetic stirrer 30 μL of i-Bu3Al/NaH solutions (ratio Al/Na = 0.8) and 9 μL of THF were added with a syringe to dry and degassed toluene (0.5 mL). At 100 °C, the polymerization starts after injection of 0.19 mL of styrene. After 8 h, the glass flask was opened in a drybox and was quickly added 0.28 mL of myrcene. The polymer was coagulated after further 72 h in methanol containing a little amount of hydrochloric acid and 2,6-bis(1,1-dimethylethyl)-4-methylphenol (BHT). The polymer was washed and dried under vacuum at room temperature until constant weight. 366 mg polymer (93% yield, Mw = 60.2, Đ = 1.8).

### 2.6. Polymer Characterizations

^1^H and ^13^C NMR analysis were recorded Bruker AV III-300, AV HD-400 and AV III-500 spectrometer at room temperature. Chemical shifts δ are reported in ppm relative to tetramethylsilane and calibrated to the residual ^1^H or ^13^C signal of the deuterated solvent.

Diffusion-ordered spectroscopy (DOSY) experiments were carried out at 298 K on a Bruker Avance 600 (Karlsruhe, Germany) spectrometer with the standard Bruker pulse program (ledbpgp2s). The latter used a double stimulated echo sequence and LED, bipolar gradient pulses for diffusion, and two spoil gradients. For the duration of the experiment (about 25 min), the pulse gradients were increased from 5% up to 95% of the maximum gradient strength in a linear ramp. The parameters were set as follows: diffusion times were 2500 ms, the eddy current delay was 5 ms, the gradient recovery delay was 0.2 ms, and the gradient pulse was 1000 ms. Individual rows of the quasi-2D diffusion databases were phased and, after Fourier transformation and baseline correction, the diffusion dimension was processed with the Bruker software Topspin3.2 and Diffusion. By Gaussian fits diffusion coefficients were calculated, using T1/T2 software of Topspin3.2.

Size exclusion chromatography (SEC) measurements were performed using a PolymerLaboratoriesGPC50 Plus (Walnut Creek, CA, USA) chromatograph (calibrated with polystyrene standards) and equipped with Deflection RI detector at 40 °C with THF as the eluent (flow of 1 mL/min).

Differential scanning calorimetry (DSC) analysis was conducted on a DSC Q2000 (New Castle, DE, USA) instrument. 2–5 mg of polymer was sealed into a DSC aluminium pan and heated from −90 to 250 °C at 10 °C/min. The reported values are those determined in the second heating cycle.

Thermal gravimetric analysis (TGA) measurements were performed on a TA Instruments Q5000 SA (New Castle, DE, USA). The heating and cooling rate was adjusted to 10 °C/min and the temperature range measured was 40–600 °C under inert atmosphere of Argon.

Water contact angle measurement (WCA) were performed on a Krüss DropShape Analyzer (Hamburg, Germany). The evaluation was performed with the program ImageJ. The samples were applied via solvent casting on glass object carriers and measured several times. The average of these values represent the contact angle.

Scanning electron microscope (SEM) measurements were recorded on a JEOL field emission scanning electron microscope JSM-7500F (Garching, Germany). The secondary electrons produced by the electrons of the beam (primary electrons) in interaction with the atoms of the object under investigation served as information source. The samples were examined at different magnifications. 

## 3. Results and Discussion

### 3.1. Homopolymerizations of Myrcene, Styrene and Isoprene

Polymyrcene (PM), polystyrene (PS) and polyisoprene (PI) were synthesized by anionic polymerization using the simple combination of i-Bu_3_Al/NaH as initiating systems at 100 °C in degassed and dry toluene solutions (runs 1–9, Table 1). A blank experiment was tried with just NaH and did not provide any results.

The effect in terms of efficiency and polymerization control of a second polar additive such as THF was observed in the homopolymerizations of M. Its absence (run 2, Table 1) had negative effects on the polymerization yield (7%) and the molecular weight distribution (MWD) was found bimodal. Reasonably, as already observed in previous studies [56,58], THF increased the reactivity of the system also at ratios 0.7 < Al:Na < 1 and shifted the equilibrium of the complexes, favoring the formation of species with active sites (s2) to which propagation reactions are restricted.

According to ^1^H NMR analysis of the vinylic proton signals at δ 4.7−5.2 ppm (see Figure S1 and Equations (E1) and (E2) of supporting information, SI), PM consisted of an almost equimolar mixture of 1,4 *cis*/*trans* and 3,4 units, since 1,2 units were revealed only in a small percentage (2–3%). The signals between 5.00–5.20 ppm were found assignable to the olefinic protons of 1,4-units (*cis* and *trans*), whereas the doublets at 4.73–4.76 ppm belonged to 3,4-units. The structural irregularity of PM emerged from ^13^C {^1^H} NMR spectra (See Figure S2, SI), in which the overlap of some signals did not allow the accurate determination of the *cis* and *trans* geometric isomers for 1,4 units. However, a deeper analysis of the aliphatic portion of ^13^C NMR spectrum (See Figure S3, SI) permitted to detect the presence of many peaks (28.55, 29.89 and 37.23 ppm) related to head-to-head and tail-to-tail regio-irregular 1,4 units along the polymer backbone. These signals have already been reported for samples obtained by cationic and coordination polymerizations of M [21,37]. In the 1980s, pioneering anionic studies have shown that M can be polymerized using sec-butyllithium (sec-BuLi) in nonpolar solvents (e.g., benzene, cyclohexane), obtaining PM with a high amount of the preferable 1,4-units [38,59]. In recent years, the prevalence of 1,4 linkages for M with the same initiator, under different experimental conditions, in both homopolymerizations and copolymerizations has been confirmed [40,60]. At 10 °C in THF, Bolton et al. reported 1,4-, 3,4-, and 1,2-contents of about 30, 60, and 10%, respectively [25]. In the ‘green’ ether solvents, like cyclopentyl methyl ether and 2-methyltetrahydrofuran, 1,2 and 3,4 units (36–86%) were found enriched [61]. *n*-BuLi in combination with tetramethylethylenediamine (TMEDA) as the modifier provides random copolymer and block copolymer of M and S in which 3,4-units can be found in range 22–39% [62]. However, the presence of a higher percentage of 3,4-addition microstructures, as in our case (47–52%), could make PM more susceptible toward sulfur vulcanization as evidenced by Sarkar et al. [23,63]. This peculiar aspect could be the subject of further investigation in the future.

Size exclusion chromatography (SEC) analysis revealed molecular weights (Mw) in the range of 50.2–89.3 KDa, a dispersity index (Đ) of 1.7–2.0 with monomodal MWD. The broader distribution of Đ, compared to the narrower values generally obtained with anionic polymerizations, can be explained by several factors. Specially, the high operating temperature (100 °C) in experimental conditions could facilitate termination reactions, as well as the presence of a TIBA, which is a chain transfer agent. Yields increased prolonging the polymerization times from 24 h (58%) to 72 h (85%), instead Mw increased, at the expense of yield, increasing the monomer)/NaH ratio. The polymer chains of PM showed low T*_g_* values (usually below −60 °C) comparable to conventional rubbers.

PS obtained by *i*-Bu_3_Al/NaH heterocomplexes (runs 5–6, Table 1) had characteristic spectra of an atactic polystyrene (for ^1^H and ^13^C NMR see Figures S6 and S7 of SI) [64]. This evidence was confirmed by the differential scanning calorimetry (DSC) analysis that showed a T*_g_* around 105 °C without any Tm. Mw followed the same trend of PM, increasing in line with the increase of the monomer/NaH ratio. For the latter set to 1450, Mw grew up to 285.1 KDa with Đ value (1.5) and yield of over 90% in 8 h. The homopolymerization of isoprene (I) has been reported in literature using similar ternary initiating systems based on alkali metal hydride, trialkylaluminium and alkoxides in cyclohexane at different temperatures (80–120 °C), achieving mainly a mixture of 1,4 and 3,4 isoprene units and small amount of 1,2 units present (3–6%) [65]. Table 1 contains data relating to isoprene polymerizations (runs 7–9). The best monomer conversion (74%) was achieved after 24 h, starting from NaH = 6.0*10^−3^ mol/L and monomer/NaH = 500.

Figure S8 in supporting information displays a typical ^1^H NMR spectrum, in which the resonances above 4.50 ppm were attributed to olefinic protons of 3,4- addition (4.65–4.72 ppm), 1,2- addition (4.80–4.90 ppm), 1,4- (*cis*- and *trans*) addition (5.02 ppm) and 1,2- addition (5.70 ppm). Isoprene polymer compositions were determined by ^1^H and ^13^C {^1^H} NMR spectroscopy in solution according to Equations (S5)–(S9) (see SI). The contents of 1,4, 3,4-, and 1,2- units in the PI were in the ranges of 30–35%, 54–56% and 11–14%, respectively. The SEC analyses revealed that PI copolymers have Mw ranging from 24.9 to 59.1 KDa with Đ of 1.5–1.6. The T*_g_* of polymers measured by DSC ranging from −14.9 to −11.4 °C. TGA curves of PM, PS and PI in argon atmosphere are reported in SI (Figures S38–S40). From the TGA curve it can be clearly seen that below 300 °C there is no noticeable weight loss underling the thermal stability of the obtained materials. Moreover, plots for myrcene and isoprene conversions versus polymerization time and evolution of molecular weight and Ð versus conversion are shown in the Section S9 of SI.

### 3.2. Synthesis and Characterization of Poly(M−co-S), Poly(M−b-S), Poly(M−co-I) Copolymers and Poly(M-co-S-co-I) Terpolymers

Copolymerization of various monomers is a powerful strategy to modify the properties of manufactured polymers satisfying specific needs, for example modifying T*_g_*, reducing crystallinity, or changing the molecular architecture of polymer backbone chains in order to obtain new materials with better properties. The polymerizations of M with S and I were performed in toluene, at 100 °C, using a monomers/NaH ratio ranging from 500 to 1450. Table 2 summarizes the main results. Polymer compositions were fully elucidated by ^1^H NMR and ^13^C {^1^H} NMR spectroscopy according to the methods reported in the supplementary material (See Equations (S1)–(S4) and (S8)–(S13), SI). NMR spectra were recorded in CDCl_3_ and 1,1,2,2 tetrachloroethane-d2 (TCDE) in order to have all the diagnostic samples signals free from deuterated solvent peak. In all copolymers 1,2 units of M were not diagnosed.

**Figure 2:**
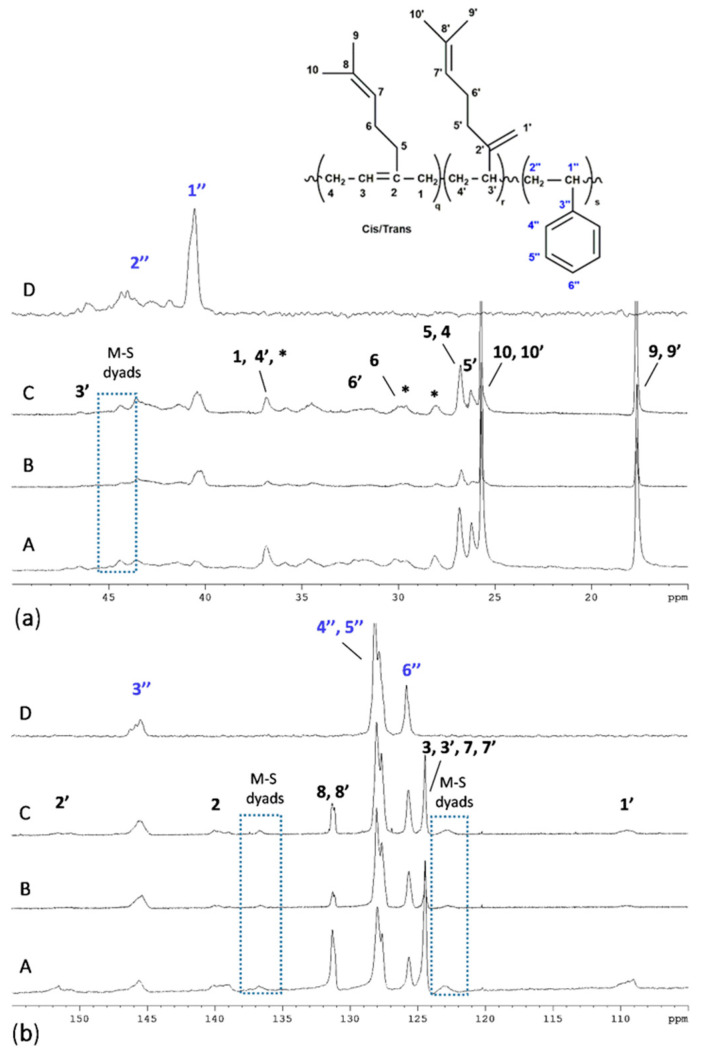
Aliphatic (**a**) and olefinic (**b**) regions of ^13^C NMR spectra (TCDE, 25 °C, 400 Mhz) of PMS, synthesized at different myrcene: styrene ratios, and of PS homopolymer. A) M:S (mol/mol) = 70:30 (run 12, Table 2); B) M:S (mol/mol) = 50:50 (run 13, Table 2); C) M:S (mol/mol) = 30:70 (run 14, Table 2); PS (run 5, Table 1). * Signals attributed to head-to-head and tail-to-tail regio-irregular 1,4 M units.

Complete assignments of ^13^C {^1^H} NMR spectra (Figure 2) of synthesized poly(M−*co*-S) copolymers (PMS) are in agreement with data given in the literature [21,23,37]. The direct comparison of carbon spectra of PMS copolymers and those of PM and PS homopolymers pointed the presence of additional signals (highlighted in Figure 2) that corresponded to the M-S dyads, which were already identified by Hulnik et al. in the emulsion cationic polymerization of M and S using a water-dispersible Lewis acid surfactant combined catalyst (LASC) [37]. It is interesting to observe the C1′’ and C2′’ signals of the styrenic unit, at higher myrcene contents (Figure 2), almost disappear simultaneously with the formation of the M-S dyads.

**Figure 3:**
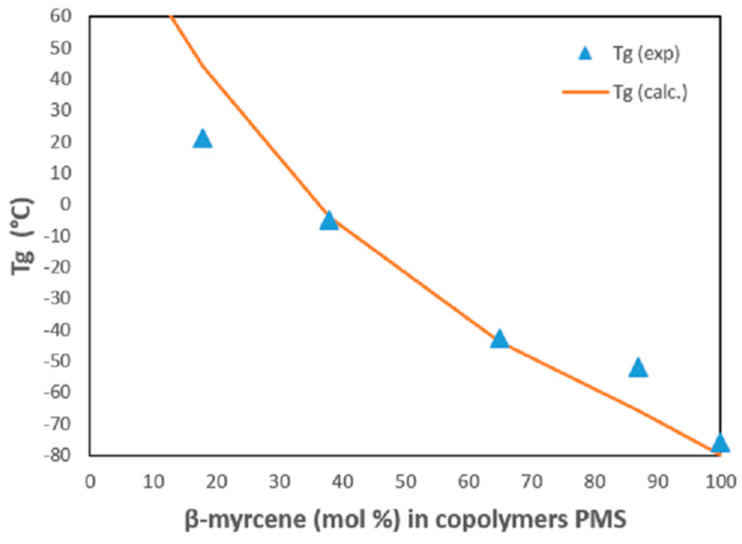
Glass transition temperatures (T*_g_*) at different β-myrcene content (mol %) incorporated in copolymers.

Head-to-head and tail-to-tail enchainments bridge of 1,4-*cis* M additions (see Figure S3 in detail, SI), previously observed in the PM, were also visible in all ^13^C {^1^H} spectra of PMS copolymers. Glass transition temperatures of PMS (runs 10–14) lie between that of the homopolymers (−76.5 and 105.5 °C for polymyrcene and atactic polystyrene, respectively) and are listed in Table 2. All the copolymers, with the exception of run 21, showed a single T*_g_* value which decreased with increasing amount of M incorporated in the copolymers. This is due to the decrease in the comonomer content of styrene having a rigid benzene ring. For comparison purposes, the Fox equation was applied for the calculation of T*_g_* values, showing a good correlation between experimental and theoretical data (Figure 3):

$$\frac{1}{T_{g}}=\;\sum \frac{W_{i}}{T_{g}{}_{i}}$$

where W*_i_* is weight fraction of component *i* and T*_g_* and T*_gi_* are the glass transition temperatures of the copolymer and of the homopolymer i, respectively.

The reactivity ratios for myrcene-styrene copolymerization were estimated by the Kelen–Tudos (KT) method (See paragraph 2 and Table T3, SI), considering the monomer feed ratios and the copolymer compositions (Table T1, SI). To minimize the composition drift, the copolymerization reactions were stopped after 30 min, corresponding to a monomers conversion of 5–9%. In Figure A2 (SI) is presented the KT plots. The difference in reactivity between myrcene and styrene (r_myr_ = 0.12 ± 0.003 and r_sty_ = 3.18 ± 0.65) indicated that S is more reactive than M. The products of r_myr_ and r_sty_ were less than the unity (r_myr_ x r_sty_ = 0.38). To evaluate the reactivity ratios it was also used modified version of Kelen–Tudos called extended Kelen–Tudos method (exKT). In this method was included a new parameter called Z, by redefining η and ξ using partial molar conversion of the monomers (see paragraph 2.2, SI). The exKT parameters were shown in the Table T4 of SI. The reactivity of the monomers was found to be r_myr_ = 0.10 ± 0.004 and r_sty_ = 3.32 ± 0.68 with r_myr_ x r_sty_ = 0.33.

The two methods (KT and exKT) are in good agreement with each other, revealing that r_sty_ > 1 and r_myr_ < 1. Thus, the polymer chains are enriched in S in the initial stages of polymerization, forming short sequences of styrene units. When the polymerizations proceed to higher monomer conversion, the monomer feed is enriched in the less reactive M favoring its incorporation in the chains. These observations suggested a tendency of S and M to form tapered polymer, as also observed in other recent anionic polymerization works [60].

Keeping in mind the idea of making different PMS architectures, consecutive addition of the two monomers (run 21) produced a poly(M−b-S) diblock copolymer due to the living anionic polymerization character. GPC analysis of synthesized sample was unimodal with Ð = 1.8, confirming the formation of AB diblock copolymer. More generally, the principal evidence on the copolymeric nature was the marked difference in Mw among poly(M−b-S) and corresponding homopolymers (PM and PS) obtained under the same experimental conditions that led to exclude the formation of a polymer blend. As expected, ^13^C NMR spectrum of poly(M−b-S) confirmed the absence of M-S dyads diagnosed for PMS copolymer (Figure S14, SI), showing only the characteristic signals of M and S homosequences. Moreover, high molecular weight (60.2 KDa) did not allow detecting the junction between the two blocks.

DOSY demonstrated definitively the copolymeric nature for the examined samples (Figures S25 and S26, SI).

The DSC curve of poly(M−b-S) displayed two T*_g_* values (98.9 °C and −52.5 °C) due to the two different long monomer sequences, which are close to those of their homopolymers. Anionic polymerization promoted by *i*-Bu_3_Al/NaH was also applied to produce poly(M−*co*-I) (PMI). Copolymers of M and I were recently synthesized by lanthanum-based catalyst for coordinative chain transfer polymerization (generating high trans-1,4 microstructure for both M and I) [66], by cationic β-diimidosulfonate lutetium catalyst (obtaining isotactic 3,4-polymyrcene and polyisoprene) [67] and by PN3 type cobalt complexes [18] (obtaining predominantly 1,4-*cis* microstructure—up to 83% for myrcene).

**Figure 4:**
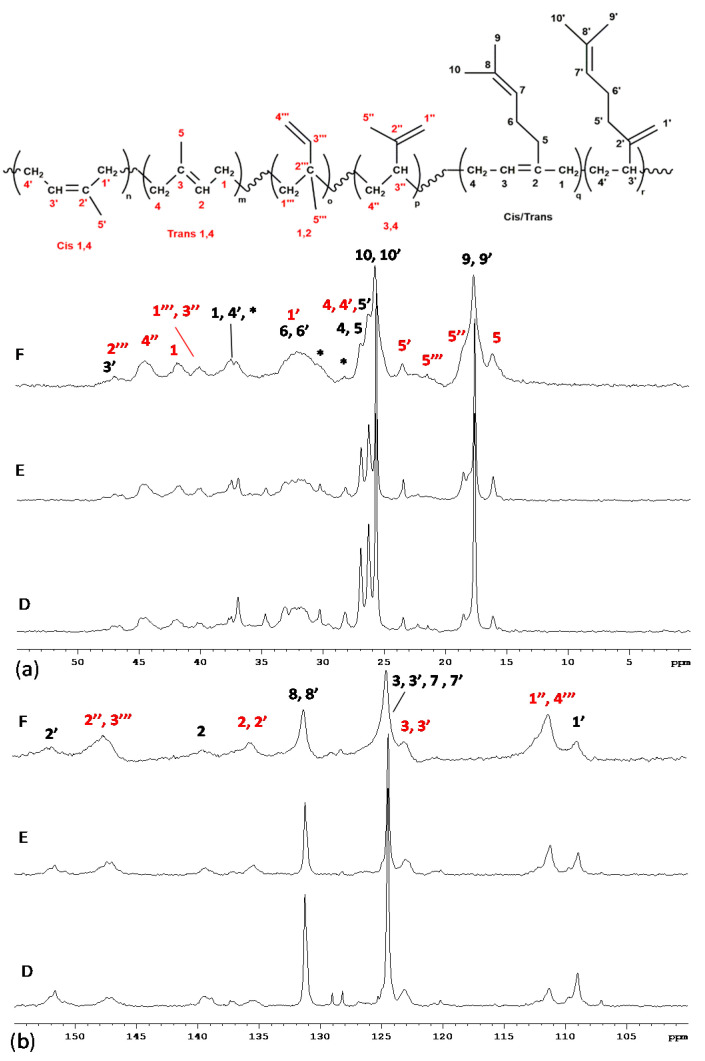
Aliphatic (**a**) and olefinic (**b**) regions of ^13^C NMR spectra (TCDE, 25 °C, 400 Mhz) of PMI, synthesized at different myrcene:isoprene ratios. D) M:I (mol/mol) = 70:30 (run 15, Table 2); E) M:I (mol/mol) = 50:50 (run 16, Table 2); F) M:I (mol/mol) = 30:70 (run 17, Table 2). * Signals attributed to head-to-head and tail-to-tail regio-irregular 1,4 M units.

The representative copolymerization data are summarized in Table 2 (runs 15–17). A conversion of 85% was obtained in 72 h at ratio M:I = 50:50 mol/mol of alimentation feed with NaH = 3.0*10^−3^ mol/L and [M+I]/NaH = 1000. The typical profile of the ^1^H NMR spectrum of PMI copolymers is shown in Figures S15 and S16 of SI. A comparison among the ^1^H NMR spectra of PM, PI and PMI is reported in Figure S15 (SI). Due to the structural similarity between monomers and the polyisoprenes sequences that contain all possible region-insertions (1,4-*cis*, 1,4-*trans*, 1,2 and 3,4 additions), many peaks of different comonomeric units felt in the same region also in the ^13^C {^1^H} NMR spectra. Thus, the difficulty in the integration of significant signals involved an uncertainty in determining polymer compositions (see SI, in particular the Equations (E10)–(E13) were based on quaternary carbons of the various concatenating units). Figure 4 shows the ^13^C NMR spectra of PMI copolymers which have been produced starting from different initial M:I (mol/mol) ratios. All the main peaks were assigned based on examples in the literature [18,21,37,67,68]. Furthermore, PMI copolymers showed a monomodal and molecular weight distribution in the range 1.7–1.9. Only one T*_g_* were found for PMI copolymers, and no melting temperatures Tm were detected.

Ter-polymerizations of myrcene, isoprene and styrene have been recently achieved by coordinative chain transfer using pentamethylcyclopentadienyl La(BH_4_)_2_(THF)_2_ combined with magnesium dialkyl and aluminum dialkyl, obtaining highly stereoregular copolymers in a wide range of compositions [31]. Three experiments (Table 2, runs 18–20) were performed to test anionic initiator systems also in the terpolymerizations of M, I and S and provided a yield on average 80%, Mw in the range of 90–145 KDa and MWD between 1.7 and 2.7 (Table 2, runs 18–20).

The structure of poly(M-*co*-S-*co*-I) (PMSI) with the assignments of the main peaks is exhibited in the Figure S21 (SI). In this case, the major complexity of PMSI NMR spectra reflects the microscopic structural irregularity, greatly limiting a depth NMR study.

TGA of copolymers synthesized revealed a good thermal stability for all the samples, with a decomposition temperature close to 350 °C (see Figures S41–S47, SI).

**Figure 5:**
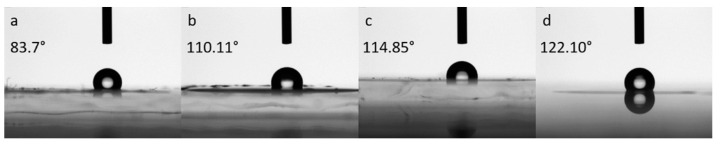
Water contact angles (WCA) measured for: PMS from run 14 of Table 2 (**a**); PMSI from run 19 of Table 2 (**b**); PI from run 7 of Table 1 (**c**) PMI from run 17 of Table 2 (d).

WCA is the most common method for determining the relative hydrophilicity of materials. Figure 5 shows the contact angles for some PMS (a), PMSI (b), PI (c), and PMI (d), respectively (see Table T7, SI). The rise in contact angle from PMS (83.7°) to PMI (122.1°) is evident. Reasonably this trend could be explained by the effect of the greater quantity in the samples of M and I which introduce small side chains in the structure of the copolymers and by their microstructure (mainly 3,4 addition), factors that increase hydrophobicity. The possibility of modulating the surface structure and properties of the copolymers by adjusting the microstructure of the segments has been already observed in previous studies [69,70].

SEM is a type of electron microscope that allow to scan the surface of a sample with a high-energy beam of electrons. SEM has the potential to generate images with a few nanometres spatial resolution, and has a relatively large depth of field (up to 100 times that of an optical microscope). This provides valuable information about the surface topography and composition of the scanned objects. The character of the surfaces is very important, as surface properties are of high interest for new elastomers in certain applications, especially such as tires or as sealings.

**Figure 6:**
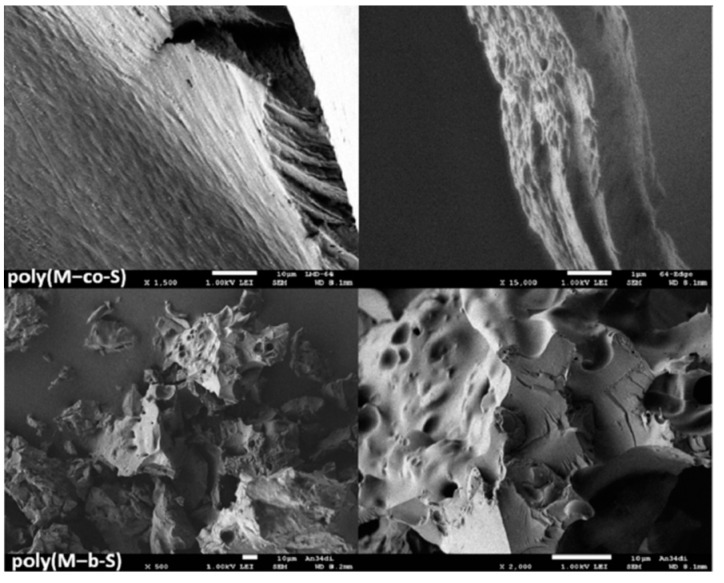
Scanning electron microscope (SEM) of poly(M−*co*-S) from run 14 of Table 2 and poly(M−b-S) from run 21 of Table 2.

The morphology of some samples, in particular poly(M−*co*-S) and poly(M−b-S) obtained, was subsequently investigated by SEM. Figure 6 shows the SEM images of runs 14 (upward) and 21 (below) collected in Table 2. These measurements show rough or wavy surface topographies of these polymers. Interestingly, edges and domains indicate the block structures present, and different dark areas in fine structures can show phase separation here (see Section S8 of the SI for more images). 

## 4. Conclusions

Homo- and co-polymerizations of myrcene with styrene and isoprene, and terpolymerization of all monomers have been reached using a soluble heterocomplexes consisting of sodium hydride in combination with triisobutylaluminum as anionic initiating system at 100 °C in toluene.

By adjusting the initial feed ratio, it was easily possible to obtain polymers in a wide range of composition with different architectures and various thermal properties. Molecular weights could be well controlled through the regulation of the monomers/NaH ratio.

Materials exhibited a T*_g_* values in the range requested for an elastomeric material and good thermal stability with a decomposition temperature exceeding 300 °C. Moreover, all the samples were fully characterized by SEC, GPC, and NMR spectroscopy (^1^H, ^13^C and DOSY experiments). In some cases, to study the surface properties, WCA and SEM characterizations were also performed. A more complete study of myrcene-styrene copolymers (PMS) was carried out, highlighting their tapered microstructures with high molecular weights (ranging from 48.1 to 159.8 KDa) and a single glass transition temperature. Indeed, for PMS the reactivity constants were determined by Kelen–Tudos (r_myr_ = 0.12 ± 0.003 and r_sty_ = 3.18 ± 0.65) and extended Kelen–Tudos (r_myr_ = 0.10 ± 0.004 and r_sty_ = 3.32 ± 0.68) methods from which we supposed the tendency of S and M to form tapered polymer.

In conclusion, here we show a convenient alkillithium-free system for the production of more sustainable elastomers by anionic polymerization, starting from biosourced monoterpenes such as myrcene. Our work is in line with the latest research in the field of elastomeric materials, which aim at the gradual replacement of fossil resources with bio-renewable monomers. The physical and mechanical tests of these myrcene-based elastomers and their behavior toward vulcanization could be the object of possible future developments and investigations.

## Figures and Tables

**Table 1:** Homopolymerizations of myrcene, styrene and isoprene, using i-Bu_3_Al/NaH as initiating systems at 100 °C.

Run ^[a]^	Monomers feed [M,S,I][mol %]	[NaH]	$\frac{\left[mon\right]}{\left[NaH\right]}$	Time [h]	Yield ^[b]^ [%]	M_W_^[c]^ [KDa]	Ð ^[e]^	T*_g_* ^[d]^ [°C]	Polymer Composition and Microstructure [%]		
									M [1,4/3,4/1,2]	S	I [1,4[*cis*]/3,4/1,2]
**1**	100,0,0	6.0 × 10^−3^	500	24	58	50.2	1.7	−61.8	100 [46/52/2]	-	-
**2 ^[e]^**	100,0,0	6.0 × 10^−3^	500	24	7	264.9	1.7	n.d.	100	-	-
**“**	“	“	“	“	“	7.9	1.7	n.d.	“	“	“
**3**	100,0,0	3.0 × 10^−3^	1000	72	85	79.6	1.7	n.d.	100 [49/48/3]	-	-
**4**	100,0,0	2.2 × 10^−3^	1450	72	40	89.3	2.0	−76.5	100 [51/47/2]	-	-
**5**	0,100,0	6.0 × 10^−3^	500	8	100	151.2	1.4	105.5	-	100	-
**6**	0,100,0	2.2 × 10^−3^	1450	8	91	285.1	1.5	n.d.	-	100	-
**7**	0,0,100	6.0 × 10^−3^	500	12	40	24.9	1.5	n.d.	-	-	100 [30[53]/56/14]
**8**	0,0,100	6.0 × 10^−3^	500	24	74	33.4	1.5	−11.4	-	-	100 [32[52]/55/13]
**9**	0,0,100	3.0 × 10^−3^	1000	24	55	59.1	1.6	−14.9	-	-	100 [35[53]/54/11]

^[a]^ Reactions were performed in toluene; (monomers) = 3.0; Al/Na = 0.8; tetrahydrofuran (THF)/Na = 50. ^[b]^ Conversion was determined by gravimetry. ^[c]^ Determined by size exclusion chromatography (SEC) in THF at 40 °C, calibrated with polystyrene standards. ^[d]^ Determined by DSC. ^[e]^ THF/Na = 0. N.d. = not determined.

**Table 2:** Co- and Ter-Polymerization of myrcene, styrene and isoprene, using i-Bu_3_Al/NaH as initiating systems at 100 °C.

Run ^[a]^	Monomers Feed [M,S,I][mol %]	[NaH]	$\frac{\left[mon\right]}{\left[NaH\right]}$	Time [h]	Yield ^[b]^ [%]	M_W_^[c]^ [KDa]	Ð ^[e]^	T*_g_* ^[f]^ [°C]	Polymer Composition and Microstructure [%]		
									M [1,4/3,4/1,2]	S	I
**10**	90,10,0	6.0 × 10^−3^	500	72	82	48.1	1.8	−47.7	87 [48/52]	13	-
**11**	90,10,0	2.2 × 10^−3^	1450	72	45	97.1	1.8	−52.2	89 [52/48]	11	-
**12**	70,30,0	2.2 × 10^−3^	1450	72	54	142.9	2.1	−42.4	65 [55/45]	35	-
**13**	50,50,0	2.2 × 10^−3^	1450	72	75	151.2	2.1	−4.9	38 [49/51]	62	-
**14**	30,70,0	2.2 × 10^−3^	1450	72	91	159.8	1.9	21.3	17 [56/44]	83	-
**15**	70,0,30	3.0 × 10^−3^	1000	72	78	71.3	1.7	−46.1	55 [59/41]	-	45
**16**	50,0,50	3.0 × 10^−3^	1000	72	85	66.5	1.9	−37.5	31 [58/42]	-	69
**17**	30,0,70	3.0 × 10^−3^	1000	72	80	62.9	1.8	−33.0	19 [53/47]	-	81
**18**	33,33,34	3.0 × 10^−3^	1000	72	83	98.7	2.7	−8.2	17	52	31
**19**	50,40,10	3.0 × 10^−3^	1000	72	81	145.2	2.2	−5.1	33	54	13
**20**	10,40,50	3.0 × 10^−3^	1000	72	80	90.9	1.7	11.0	8	45	47
**21 ^[e]^**	50,50,0	3.0 × 10^−3^	1000	8 + 72	88	60.2	1.8	98.9	36 [46/54]	64	-
**’**	’	’	’	’	’	’	’	−52.5	’	’	’

^[a]^ Reactions were performed in toluene; monomers = 3.0; Al/Na = 0.8; THF/Na = 50. ^[b]^ Conversion was determined by gravimetry. ^[c]^ Determined by SEC in THF at 40 °C, calibrated with polystyrene standards. ^[d]^ Determined by DSC. ^[e]^ Successive monomer additions (1st: styrene; 2nd: myrcene).

## Data Availability

The data presented in this study are available on request from the corresponding author.

## Supplementary Materials

The following are available online at https://www.mdpi.com/2073-4360/13/5/838/s1, Figures S1–S25: 1H, 13C and DOSY NMR of the representive samples; Figures S26–S37: DSC thermograms of representive samples; Figures S38–S47: TGA thermograms of representive samples; Figures S48–S61 GPC curves of representive samples; Figure S61: Conversion vs. time plot for isoprene polymerization; plots of MW (and Ð) vs conversion; Figure S62: Conversion vs. time plot for myrcene polymerization; plots of MW (and Ð) vs conversion. Table T1: Copolymerization of M and S with i-Bu3Al/NaH; Table T2: Reactivity ratio for copolymerization of M and S; Table S3: FR and KT parameters for PMS copolymers; Table T4: Reactivity ratio for copolymerization of M and S; Table S5: exKT parameters for PMS copolymers; Table T6: Reactivity ratio for copolymerization of M and S; Table T7: Contact angles for tested samples. Equations (E1)–(E16): Analysis of polymer compositions from NMR.
