# Supplementary material for: Sustainable Myrcene-Based Elastomers via a Convenient Anionic Polymerization
Source: Polymers (Basel). 2021 Mar 9;13(5):838. doi: 10.3390/polym13050838 (PMC7967150; doi:10.3390/polym13050838)
Supplement: Supplementary file 1 [file polymers-13-00838-s001.pdf]

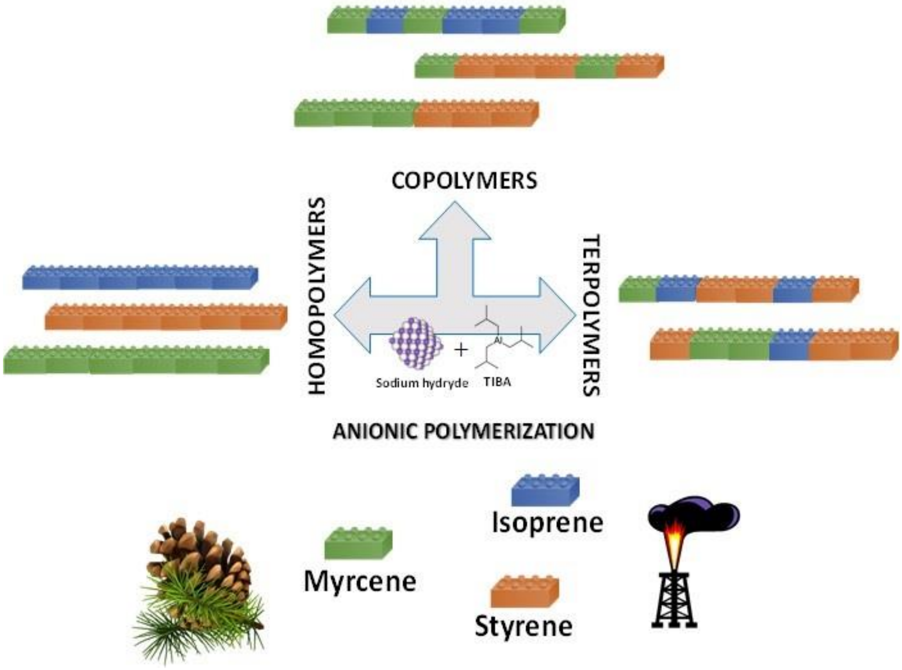

David Hermann Lamparelli, Magdalena Maria Kleybolte , Malte Winnacker and Carmine Capacchione

Sustainable myrcene-based elastomers via a convenient anionic polymerization

Sustainable myrcene-based elastomers via a convenient anionic polymerization

David Hermann Lamparelli[a], Magdalena Maria Kleybolte,[b] Malte Winnacker, *[b,c] and Carmine Capacchione *[b]

Abstract: Soluble heterocomplexes consisting of sodium hydride in combination with trialkylaluminum derivatives have been used as anionic initiating systems at 100 °C in toluene for convenient homo-, co- and ter-polymerization of myrcene with styrene and isoprene. In this way it has been possible to obtain elastomeric materials in a wide range of compositions with interesting thermal profiles and different polymeric architectures by simply modulating the alimentation feed and the [monomers]/[initiator systems] ratio. Especially, a complete study of the myrcene-styrene copolymers (PMS) was carried out, highlighting their tapered microstructures with high molecular weights (up to 159.8 KDa) and a single glass transition temperature. For PMS copolymer reactivity ratios, rmyr = 0.12 ± 0.003 and rsty =3.18 ± 0.65 and rmyr = 0.10 ± 0.004 and rsty = 3.32 ± 0.68 were determined according to the Kelen-Tudos (KT) and extended Kelen-Tudos (exKT) methods, respectively. Finally, this study showed an economic and alternative approach for the production of various elastomers by anionic copolymerization of renewable terpenes, such as myrcene, with commodities.

[a] *Department of Chemistry and Biology "Adolfo Zambelli", University of Salerno, Giovanni Paolo II Str., 84084 Fisciano, Italy.*
E-mail: ccapacchione@unisa.it
[b] *WACKER-Chair of Macromolecular Chemistry, Technische Universität München Lichtenbergstraße 4 Str., 85747 Garching bei München, Germany.*
[c] *Catalysis Research Center (CRC) Ernst-Otto-Fischer-Straße 1, Garching bei München 85748, Germany.*
E-mail: malte.winnacker@tum.de

Contents

1. Analysis of polymer compositions [1–6] 6

2 Determination of Reactivity Ratios 8

2.1 Fineman-Ross method 8

2.2 Kelen-Tudos method 8

2.3 Extended Kelen-Tudos method 9

3. NMR Characterizations 11

Figure S1. ^{1}H NMR spectrum ($CDCl_3$, 25 °C, 400 MHz) of PM from run 1 of Table 1 11

Figure S2. ^{13}C NMR spectrum ($CDCl_3$, 25 °C, 400 MHz) of PM from run 1 of Table 1 11

Figure S3. Enlargement of aliphatic region of ^{13}C NMR spectrum ($CDCl_3$, 25 °C, 400 MHz) of PM from run 1 of Table 1 12

Figure S4. ^{1}H NMR spectrum (TCDE, 25 °C, 400 MHz) of PM from run 4 of Table 1 12

Figure S5. ^{13}C NMR spectrum (TCDE, 25 °C, 400 MHz) of PM from run 4 of Table 1 13

Figure S6. ^{1}H NMR spectrum (TCDE, 25 °C, 400 MHz) of PS from run 5 of Table 1 13

Figure S7. ^{13}C NMR spectrum (TCDE, 25 °C, 400 MHz) of PS from run 5 of Table 1 14

Figure S8. ^{1}H NMR spectrum (TCDE, 25 °C, 400 MHz) of PI from run 7 of Table 1 14

Figure S9. ^{13}C NMR spectrum (TCDE, 25 °C, 400 MHz) of PI from run 7 of Table 1 15

Figure S10. ^{1}H NMR spectrum (TCDE, 25 °C, 400 MHz) of PMS from run 12 of Table 2 15

Figure S11. ^{13}C NMR spectrum (TCDE, 25 °C, 400 MHz) of PMS from run 12 of Table 2 16

Figure S12. ^{1}H NMR spectrum (TCDE, 25 °C, 400 MHz) of PMS from run 13 of Table 2 16

Figure S13. ^{1}H NMR spectrum (TCDE, 25 °C, 400 MHz) of PMS from run 14 of Table 2 17

Figure S14. ^{13}C NMR spectra (TCDE, 25 °C, 400 MHz) of PMS copolymer from run 13 (a) and PMS diblock from run 21 (b) of Table 2 18

Figure S15. ^{1}H NMR spectra (TCDE, 25 °C, 400 MHz) of PM (run 3), PI (run 7) and PMI (run 16) from Table 2 19

Figure S16. ^{1}H NMR spectrum (TCDE, 25 °C, 400 MHz) of PMI from run 16 of Table 2 19

Figure S17. ^{13}C NMR spectrum (TCDE, 25 °C, 400 MHz) of PMI from run 16 of Table 2 20

Figure S18. ^{13}C NMR spectrum (TCDE, 25 °C, 400 MHz) of PMI from run 17 of Table 2 20

Figure S19. ^{13}C NMR spectrum (TCDE, 25 °C, 400 MHz) of PMI from run 18 of Table 2 21

Figure S20. ^{1}H NMR spectrum (TCDE, 25 °C, 400 MHz) of PMSI from run 18 of Table 2 21

Figure S21. ^{13}C NMR spectrum (TCDE, 25 °C, 400 MHz) of PMSI from run 18 of Table 2 22

Figure S22. ^{13}C NMR spectrum ($CDCl_3$, 25 °C, 400 MHz) of PMSI from run 19 of Table 2 23

Figure S23. ^{13}C NMR spectrum (TCDE, 25 °C, 400 MHz) of PMSI from run 20 of Table 2 23

Figure S24. DOSY NMR spectrum ($CDCl_3$, 25 °C, 600 MHz) of PMS copolymers from run 13 of Table 2 24

Figure S25. DOSY NMR spectrum ($CDCl_3$, 25 °C, 600 MHz) of PMS diblock copolymers from run of 21 Table 2 25

4. DSC Thermal Analysis 26

Figure S26. DSC thermogram of PM from run 1 of Table 1. 26

Figure S27. DSC thermogram of PM from run 4 of Table 1. 26

Figure S28. DSC thermogram of PS from run 5 of Table 1. 27
Figure S29. DSC thermogram of PI from run 8 of Table 1. 27
Figure S30. DSC thermogram of PI from run 9 of Table 1. 28
Figure S31. DSC thermogram of PMS from run 10 of Table 2. 28
Figure S32. DSC thermogram of PMS from run 12 of Table 2. 29
Figure S33. DSC thermogram of PMS from run 14 of Table 2. 29
Figure S34. DSC thermogram of PMI from run 16 of Table 2. 30
Figure S35. DSC thermogram of PMSI from run 18 of Table 2. 30
Figure S36. DSC thermogram of PMSI from run 20 of Table 2. 31
Figure S37. DSC thermogram of diblock PMS from run 21 of Table 2. 31
5. TGA Analysis 32
Figure S38. TGA thermogram of PM from run 1 of Table 1. 32
Figure S39. TGA thermogram of PS from run 5 of Table 1. 32
Figure S40. TGA thermogram of PI from run 8 of Table 1. 33
Figure S41. TGA thermogram of PMS from run 12 of Table 2. 33
Figure S42. TGA thermogram of PMS from run 14 of Table 2. 34
Figure S43. TGA thermogram of PMI from run 15 of Table 2. 34
Figure S44. TGA thermogram of PMI from run 16 of Table 2. 35
Figure S45. TGA thermogram of PMI from run 17 of Table 2. 35
Figure S46. TGA thermogram of PMSI from run 20 of Table 2. 36
Figure S47. TGA thermogram of diblock PMS from run 21 of Table 2. 36
6. GPC Analysis 37
Figure S48. GPC curve of PM from run 1 of Table 1. 37
Figure S49. GPC curve of PM from run 2 of Table 1. 37
Figure S50. GPC curve of PM from run 3 of Table 1. 38
Figure S51. GPC curve of PM from run 4 of Table 1. 38
Figure S52. GPC curve of PS from run 5 of Table 1. 39
Figure S53. GPC curve of PI from run 8 of Table 1. 39
Figure S54. GPC curve of PI from run 9 of Table 1. 40
Figure S55. GPC curve of PMS from run 10 of Table 2. 40
Figure S56. GPC curve of PMS from run 12 of Table 2. 41
Figure S57. GPC curve of PMS from run 13 of Table 2. 41
Figure S58. GPC curve of PMI from run 16 of Table 2. 42
Figure S59. GPC curve of PMI from run 17 of Table 2. 42
Figure S60. GPC curve of PMSI from run 20 of Table 2. 43
Figure S61. GPC curve of diblock PMS from run 21 of Table 2. 43
7. CAM Analysis 44
8. SEM Analysis 46

9. Kinetic studies 47

Figure S61. Conversion vs. time plot for isoprene polymerization (on left); plots of M_W (and Đ) vs conversion (on right) 47

Figure S62. Conversion vs. time plot for myrcene polymerization (on left); plots of M_W (and Đ) vs conversion (on right) 47

10. References 47

11. Author Contributions 47

1. Analysis of polymer compositions [1–6]

1.1 Determination of $M^{3,4}$ and $M^{1,4}$ contents in PM and PMS copolymers by ^1H NMR (TCE-d_2, 25 °C)

- $M^{3,4}(mol\%) = \frac{A_B}{A_A+\frac{A_B}{2}+A_C} \cdot 100$ **Equation E1**

Where: A_A is the area of the signals $\mathbf{M}^{1,4}{}_3$, $\mathbf{M}^{1,4}{}_7$, $\mathbf{M}^{1,2}{}_7$ and $\mathbf{M}^{3,4}{}_7$ (δ = 5.12 ppm); A_B is the area of the signal $\mathbf{M}^{3,4}{}_1$ (δ = 4.77 ppm); A_C is the area of the signal $\mathbf{M}^{1,2}{}_3$ (δ = 5.63 ppm);

- $M^{1,4}(mol\%) = \frac{A_A-(\frac{A_B}{2}+A_C)}{A_A+\frac{A_B}{2}+A_C} \cdot 100$ **Equations E2**

- $M^{1,2}(mol\%) = 100 - (M^{3,4}(mol\%) + M^{1,4}(mol\%))$

1.2 Determination of M and S contents in PSM copolymers by ^1H NMR (TCE-d_2, 25 °C)

- $M(mol\%) = \frac{\frac{1}{2}(A_A+\frac{A_B}{2})}{\frac{1}{2}\left(A_A+\frac{A_B}{2}\right)+\frac{A_C}{5}} \cdot 100$ **Equation E3**

Where: A_A is the area of the signals $\mathbf{M}^{1,4}{}_3$, $\mathbf{M}^{1,4}{}_7$ and $\mathbf{M}^{3,4}{}_7$ (δ=5.12 ppm); A_B is the area of the signal $\mathbf{M}^{3,4}{}_1$ (δ=4.77 ppm); A_C is the area of the signals S_4, S_5 and S_6 (δ=6.45-7.45 ppm)

- $S\ (mol\%) = 100 - M(mol\%)$ **Equation E4**

1.3 Determination of $I^{3,4}$, $I^{1,4}$ and $I^{1,2}$ contents in PI by ^1H NMR ($CDCl_3$ or TCE-*d*2, 25 °C)

- $I^{3,4}(mol\%) = \frac{\frac{B_A}{2}}{\frac{B_A}{2}+(B_B-2B_C)+B_c} \cdot 100$ **Equation E5**

Where: B_A is the area of the signals $\mathbf{I}^{3,4}{}_1$ (δ = 4.60-4.80 ppm); B_B is the area of the signal $\mathbf{I}^{1,2}{}_4$ and $\mathbf{I}^{1,4(cis+trans)}{}_3$ (δ = 4.80-5.40 ppm) and B_C is the area of the signal $I^{1,2}{}_3$ (δ = 5.75-5.90 ppm)

- $I^{1,4}(mol\%) = \frac{(B_B-2B_C)}{\frac{B_A}{2}+(B_B-2B_C)+B_c} \cdot 100$ **Equation E6**

- $I^{1,2}(mol\%) = 100 - I^{3,4}(mol\%) - I^{1,4}(mol\%)$ **Equation E7**

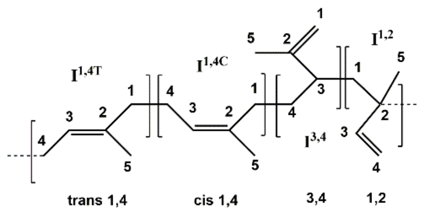

1.4 Determination of $I^{1,4\ cis}$ and $I^{1,4\ trans}$ contents in PI by ^{13}C NMR ($CDCl_3$, 25 °C)

- $I^{1,4}\ cis\ (mol\%) = \frac{C_A}{C_A+C_B} \cdot 100$ **Equation E8**

Where: C_A is the area of the signal $\mathbf{I}^{1,4\ CIS}{}_5$ (δ=23.5 ppm); C_B is the area of the signal $\mathbf{I}^{1,4\ TRANS}{}_5$ (δ=16.3 ppm)

- $I^{1,4}\,trans\,(mol\%) = 100 - I^{1,4}\,cis\,(mol\%)$ **Equation E9**

1.5 Determination of M and I contents in PMI copolymers by ^{13}C NMR ($CDCl_3$, 25 °C)

- $M(mol\%) = \frac{C_A}{C_A+C_B+C_C} \cdot 100$ **Equation E10**

where: C_A is the sum of the areas related to the signals $\mathbf{M}^{1,4\ (cis+trans)}{}_2$ (δ=139.3 ppm) and $\mathbf{M}^{3,4}{}_{2'}$ (δ=151.6 ppm); C_B is the area of the signals $\mathbf{I}^{3,4}{}_1$ and $\mathbf{I}^{1,2}{}_4$ (δ=111.4 ppm); C_c is the area of the signals $\mathbf{I}^{1,4}{}_2$ (δ=135.6 ppm)

- $I(mol\%) = 100 - M(mol\%)$ **Equation E11**

1.6 Determination of $M^{3,4}$ and $M^{1,4}$ contents in PMI copolymers by ^{13}C NMR ($CDCl_3$, 25 °C)

- $M^{3,4}(mol\%) = \frac{C_D}{C_A} \cdot 100$ **Equation E12**

where: C_A is the sum of the areas related to the signals $\mathbf{M}^{1,4\ (cis+trans)}{}_2$ (δ=139.3 ppm) and $M^{3,4}{}_{2'}$ (δ=151.6 ppm); C_D is the area of the signals $\mathbf{M}^{3,4}{}_{2'}$ (δ=151.6 ppm)

- $M^{1,4}(mol\%) = 100 - M^{3,4}(mol\%)$ **Equation S13**

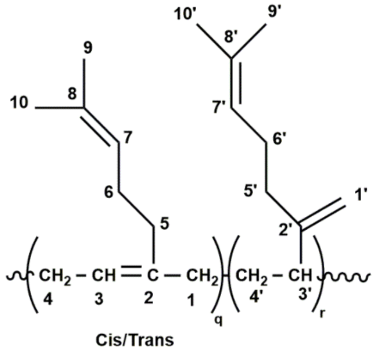

1.5 Determination of M and I contents in PMI copolymers by ^{13}C NMR (TCE-*d*2, 25 °C)

- $M(mol\%) = \frac{C_A}{C_A+C_B+C_C+C_E} \cdot 100$ **Equation E14**

where: C_A is the sum of the areas related to the signals $\mathbf{M}^{1,4\ (cis+trans)}{}_2$ (δ=139.3 ppm) and $\mathbf{M}^{3,4}{}_{2'}$ (δ=151.6 ppm); C_B is the area of the signals $\mathbf{I}^{3,4}{}_1$ and $\mathbf{I}^{1,2}{}_4$ (δ=111.4 ppm); C_c is the area of the signals $\mathbf{I}^{1,4}{}_2$ (δ=135.6 ppm); C_E is the area of the signals $\mathbf{S}_{3''}$ (δ=145.5 ppm) (see **Fig. S6**)

- $I(mol\%) = \frac{C_B+C_C}{C_A+C_B+C_C+C_E} \cdot 100$ **Equation E15**

- $S(mol\%) = \frac{C_E}{C_A+C_B+C_C+C_E} \cdot 100$ **Equation E16**

2 Determination of Reactivity Ratios

2.1 Fineman-Ross method

Fineman-Ross equation: **$[(f-1)/f]F) = r_1 - r_2 F^2/f$**

where: f = myrcene/styrene molar ratios in the polymer (by ^{1}H NMR analysis)

F = myrcene/styrene molar ratios in the feed

r_1, r_2 = reactivity ratios

Table T1. Copolymerization of M and S with i-Bu_3Al/NaH.

**Run** [a]	**Feed composition**		**Yield**	**Copolymer composition**		**M/S (molar ratio)**		**F^2/f**	**(f-1/f)F**
	**M**	**S**		**M**	**S**	**in feed (F)**	**in copolymer (f)**		
	**(*mol*)**	**(*mol*)**	**%**	**(*mol%*)**	**(*mol%*)**				
**1**	0.42	0,42	9.1	21	79	1,00	0,26	3,76	-2,76
**2**	0.42	0,13	8,4	42	58	3,24	0,72	14,47	-1,23
**3**	0.42	0,11	9,5	45	55	3,81	0,82	17,72	-0,85
**4**	0.42	0,07	6,8	49	51	5,99	0,96	37,38	0,24
**5**	0.42	0,04	8,6	66	34	10,5	1,94	56,73	5,09

[a] Reaction conditions: 88 µL, toluene 0.5 mL, tetrahydrofuran 25 µL, 100 °C, 30 min.

Table T2. Reactivity ratio for copolymerization of M and S.

**Iniziator**	***r_1***	***r_2***	***r_1•r_1***
i-Bu_3Al/NaH	0.13 ± 0.002	3.47 ± 0.91	0.45

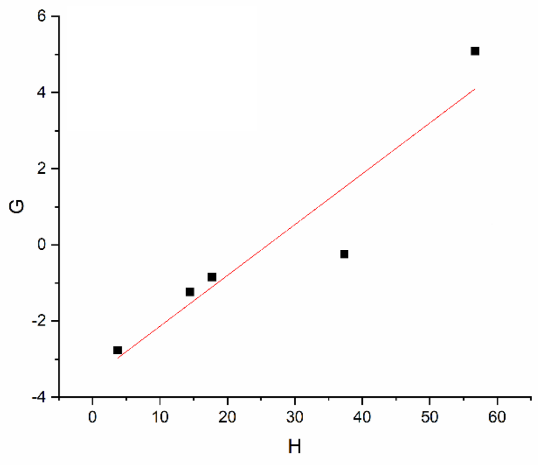

Figure A1. Fineman-Ross plot for copolymerization of myrcene and styrene with i-Bu3Al/NaH as initiating system at 100 °C in toluene and in the presence of THF (F = [Myr]/[Sty] in feed, f = [Myr]/[Sty] in copolymers, r_{Myr} = 0.13 ± 0.002 and r_{Sty} = 3.47 ± 0.91).

2.2 Kelen-Tudos method

The reactivity ratio can also be estimated with Kelen-Tudos equation (**$\eta = (r_1+r_2/\alpha)\xi - r_2/\alpha$**) which variables are expressed as:

G = (f-1/f)F
H = F^2/f
$\eta = G/(\alpha+H)$

$\xi = H/(\alpha + H)$

Where η and ξ are the functions of the parameters G and H, and α is a constant equal to $(H_{max} \times H_{min})^{1/2}$, H_{max} and H_{min} being the lowest and highest H values, respectively.
The intercepts at ξ = 0 and ξ = 1 of the η versus ξ plot gives $-r_1/\alpha$ and r_2

Table T3. FR and KT parameters for PMS copolymers

Sample	$F = M_1/M_2$	$f = m_1/m_2$	H	G	ξ	η
RR1	1,00	0,26	3,76	-2,76	0,20	-0,150
RR2	3,24	0,72	14,4	-1,23	0,50	-0,042
RR3	3,81	0,82	17,7	-0,85	0,55	-0,026
RR4	5,99	0,96	37,4	-0,24	0,72	-0,004
RR5	10,4	1,94	56,7	5,09	0,80	0,071

$\alpha = (H_{min} \times H_{max})^{1/2} = 14,60$.

Table T4. Reactivity ratio for copolymerization of M and S.

Iniziator	r_1	r_2	$r_1 \bullet r_1$
$i\text{-}Bu_3Al/NaH$	0.12 ± 0.003	3.18 ± 0.65	0.38

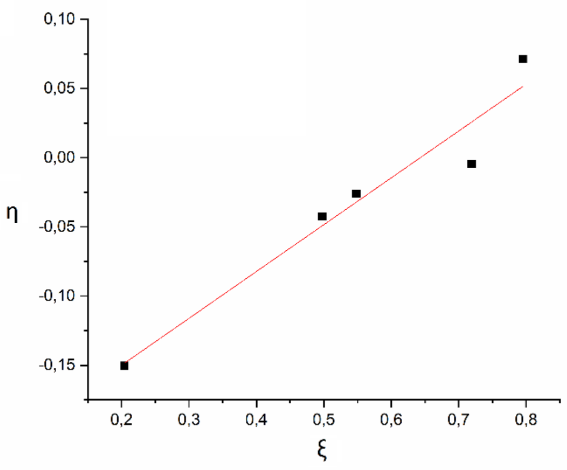

Figure A2. Determination of reactivity ratios using Kelen-Tudos method for PMS copolymers (r_{Myr} = 0.12 ± 0.003 and r_{Sty} = 3.18 ± 0.65).

2.3 Extended Kelen-Tudos method

$Z = \log(1 - S_1)/\log(1 - S_2)$ where S_1 and S_2 are the partial molar conversions:

$S_2 = w\,(\mu + F)/(\mu + f) \times 100$, w = percentage of conversions, $\mu = M_{wt\,2}/M_{wt\,1}$

$S_1 = S_2 f/F$;

They derived an expression, H = F2 / f and G = F (f – 1) / f In this method also, r2 and r1 can be readily obtained either graphically or computation using the least square methods by plotting 1 versus 0. The 1 versus 0 plot gives a straight line between 0 and 1 provided the system can be adequately described by the conventional copolymerization composition equation.

Table T5. exKT parameters for PMS copolymers

Sample	S_1	S_2	Z	H	G	ξ	η
RR1	0,041	0,155	0,249	4,26	-2,93	0,186	-0,129
RR2	0,050	0,225	0,202	17,6	-1,36	0,486	-0,038
RR3	0,059	0,274	0,189	22,8	-0,95	0,550	-0,023
RR4	0,043	0,266	0,140	48,4	-0,28	0,722	-0,004
RR5	0,066	0,358	0,154	81,2	6,09	0,814	0,061

$\alpha = (H_{min} \times H_{max})^{1/2} = 18,60$, $\mu = 0,765$.

Table T6. Reactivity ratio for copolymerization of M and S.

Iniziator	r_1	r_2	r_1•r_1
i-Bu_3Al/NaH	0.10 ± 0.004	3.32 ± 0.68	0.33

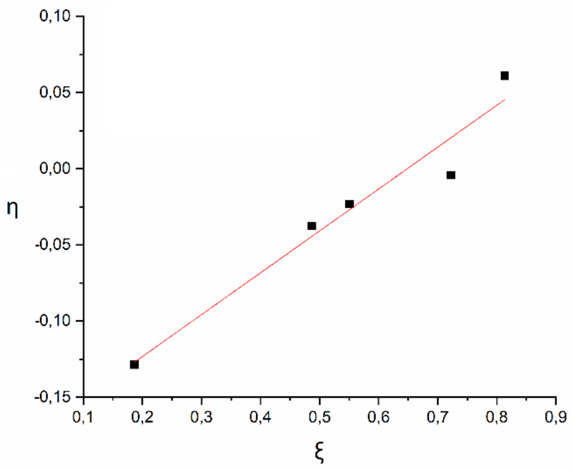

Figure A3. Determination of reactivity ratios using extended Kelen-Tudos method for PMS copolymers (r_{Myr} = 0.10 ± 0.004 and r_{Sty} = 3.32 ± 0.68).

3. NMR Characterizations

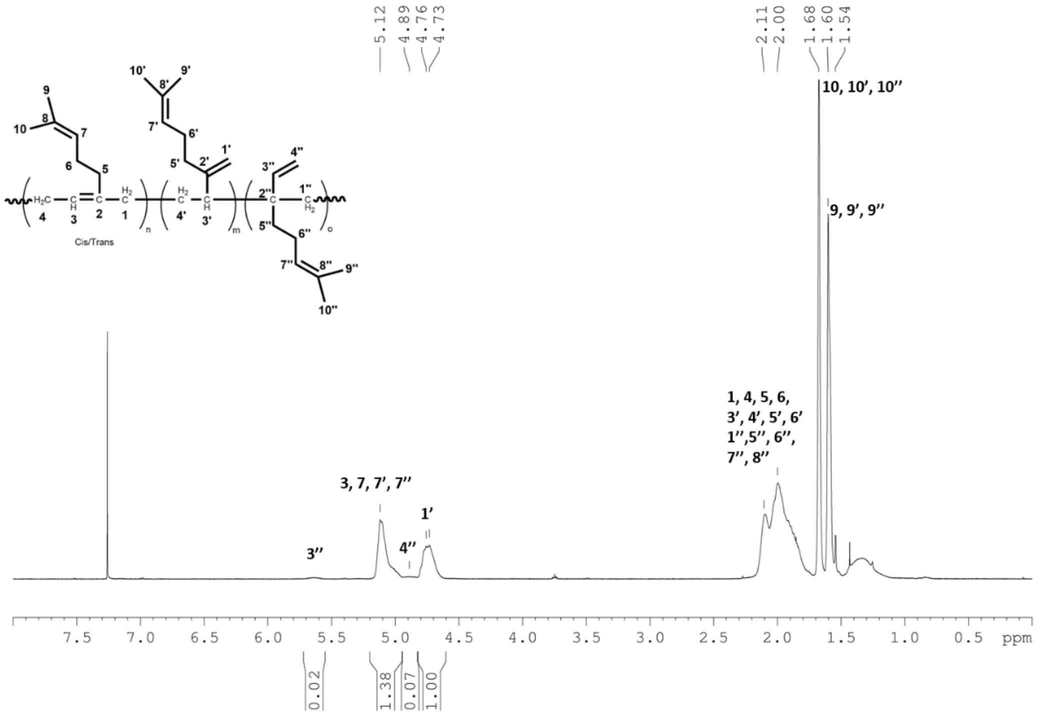

Figure S1. ^{1}H NMR spectrum ($CDCl_3$, 25 °C, 400 MHz) of PM from run 1 of **Table 1**

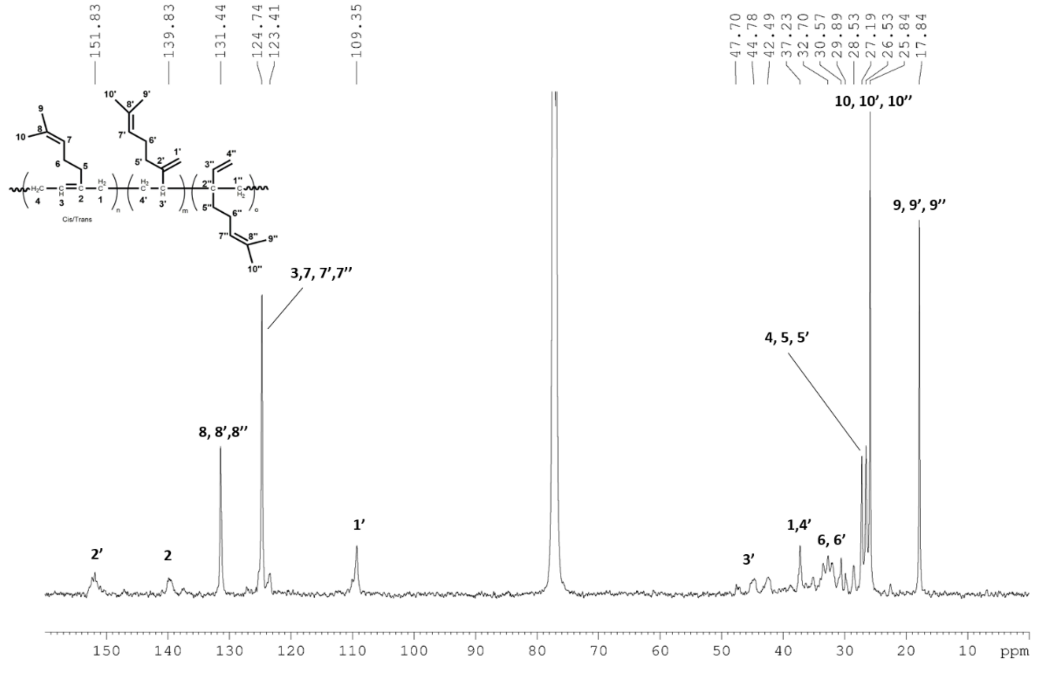

Figure S2. ^{13}C NMR spectrum ($CDCl_3$, 25 °C, 400 MHz) of PM from run 1 of **Table 1**

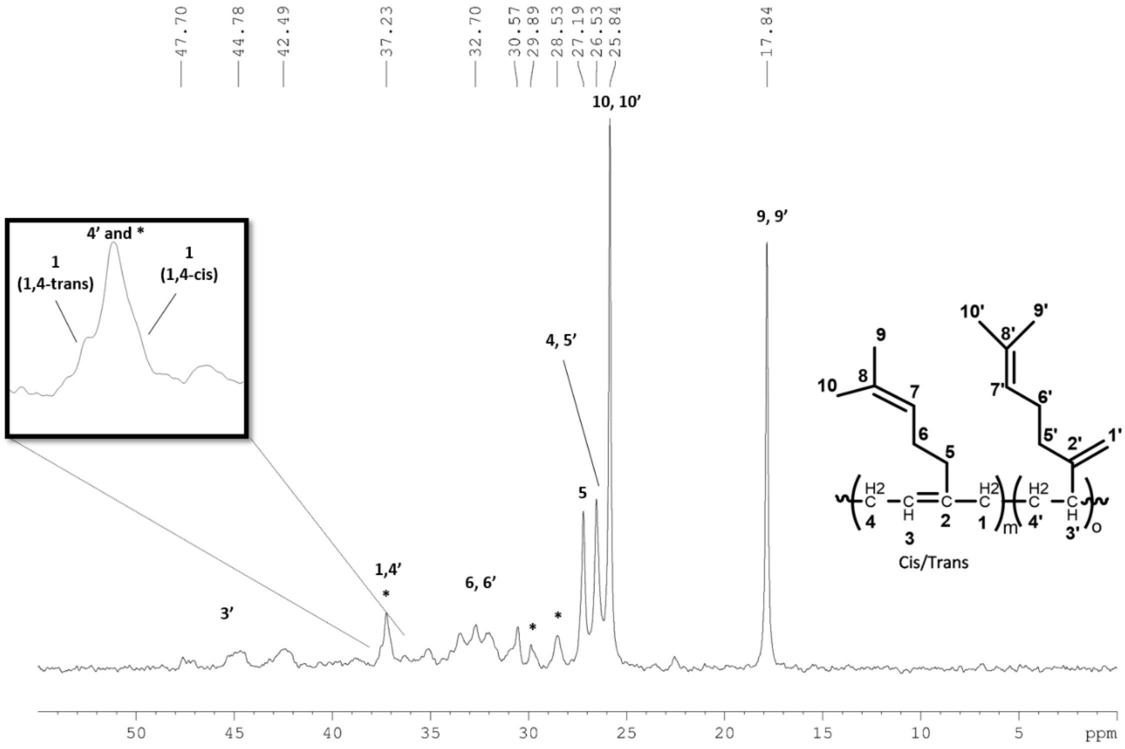

Figure S3. Enlargement of aliphatic region of ^{13}C NMR spectrum ($CDCl_3$, 25 °C, 400 MHz) of PM from run 1 of **Table 1**

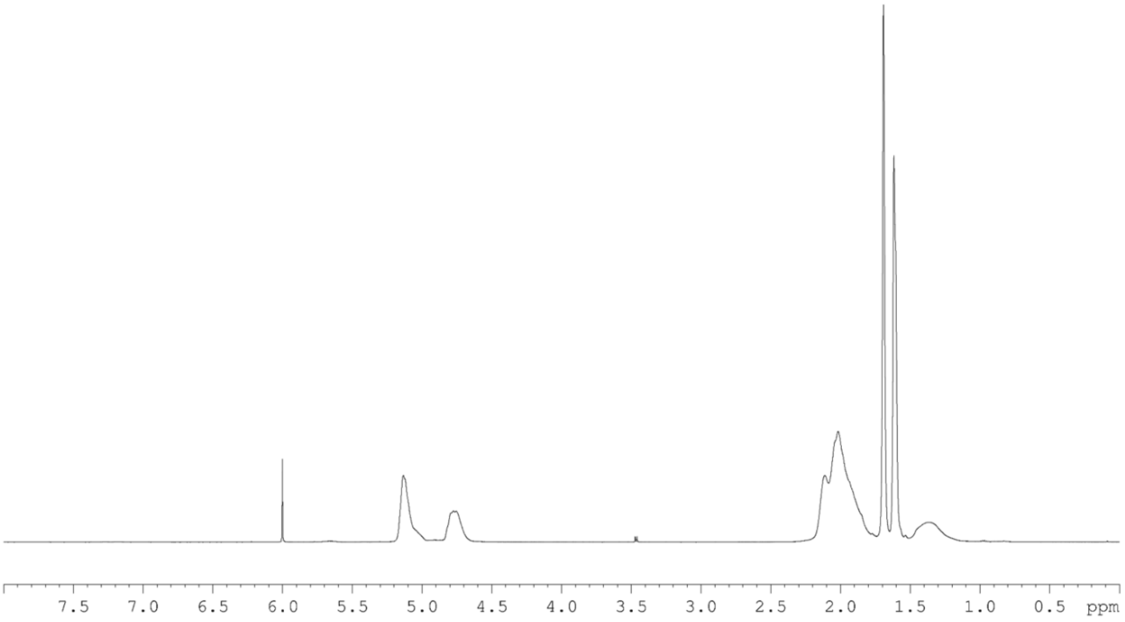

Figure S4. ^{1}H NMR spectrum (TCDE, 25 °C, 400 MHz) of PM from run 4 of **Table 1**

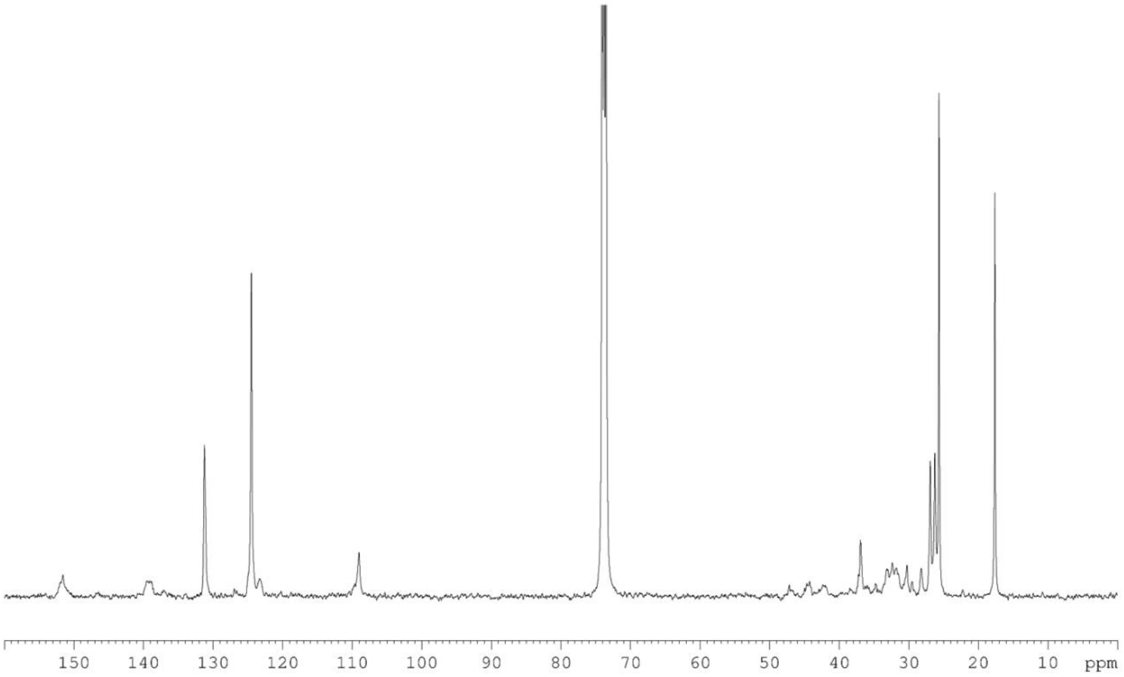

Figure S5. ^{13}C NMR spectrum (TCDE, 25 °C, 400 MHz) of PM from run 4 of **Table 1**

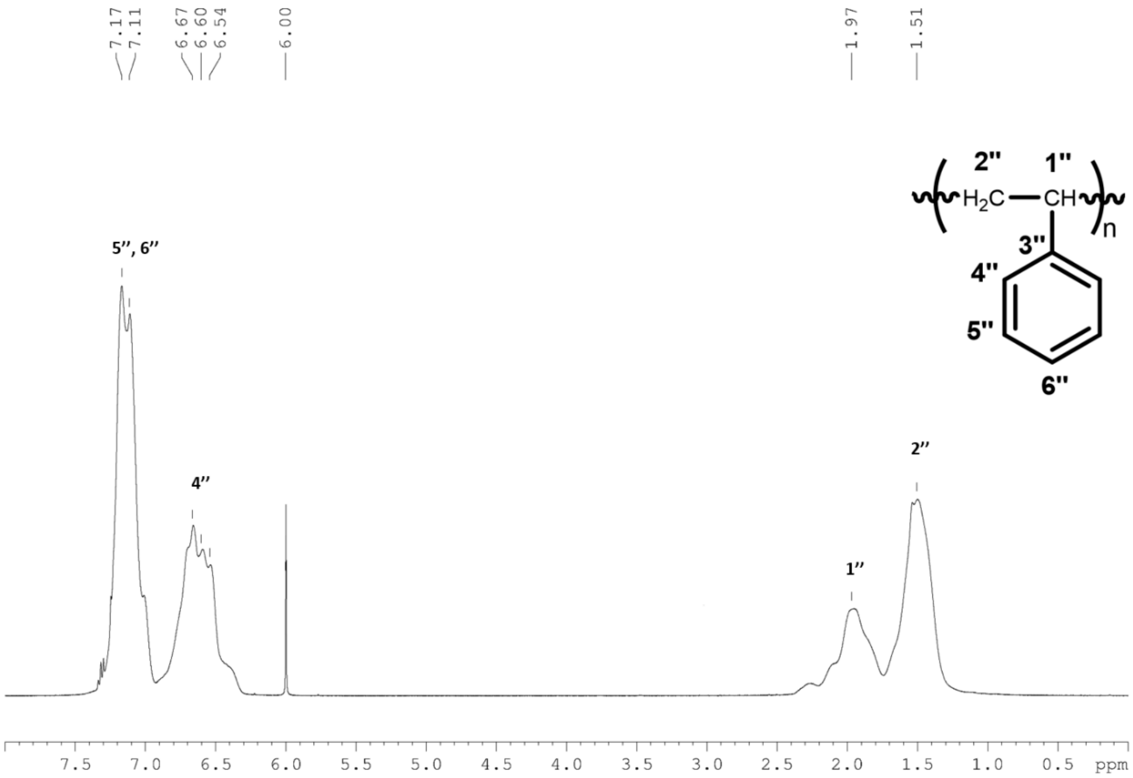

Figure S6. ^{1}H NMR spectrum (TCDE, 25 °C, 400 MHz) of PS from run 5 of **Table 1**

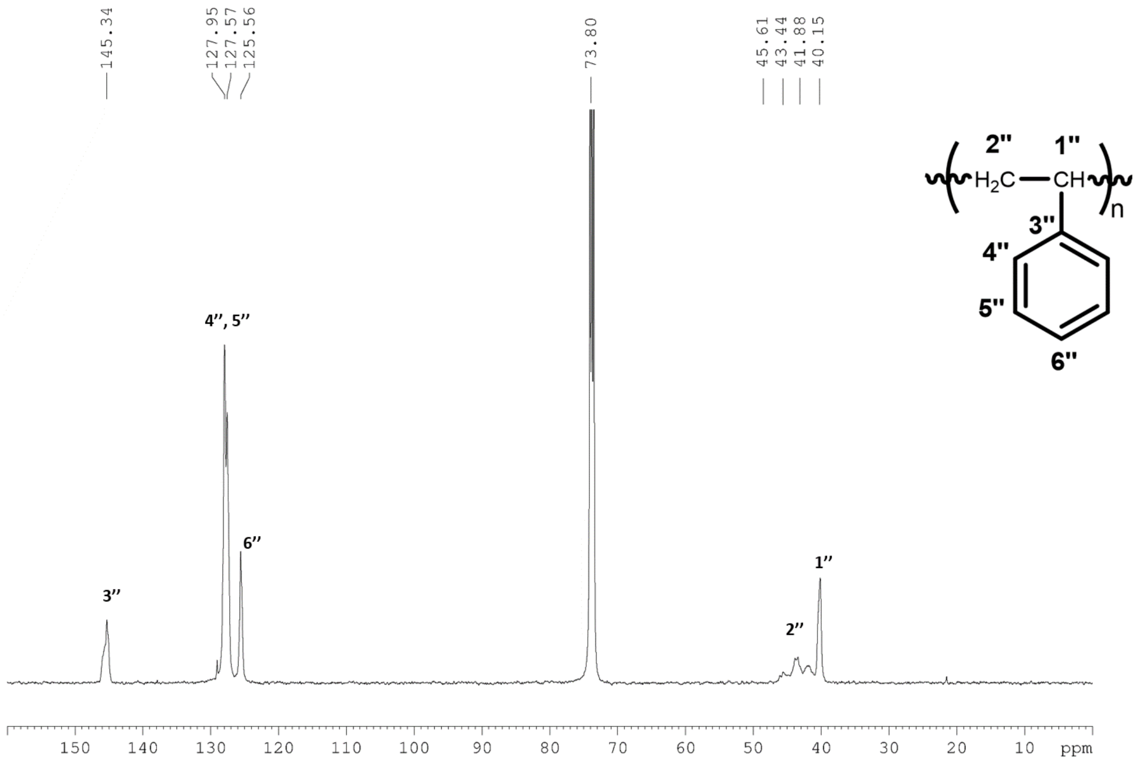

Figure S7. ^{13}C NMR spectrum (TCDE, 25 °C, 400 MHz) of PS from run 5 of **Table 1**

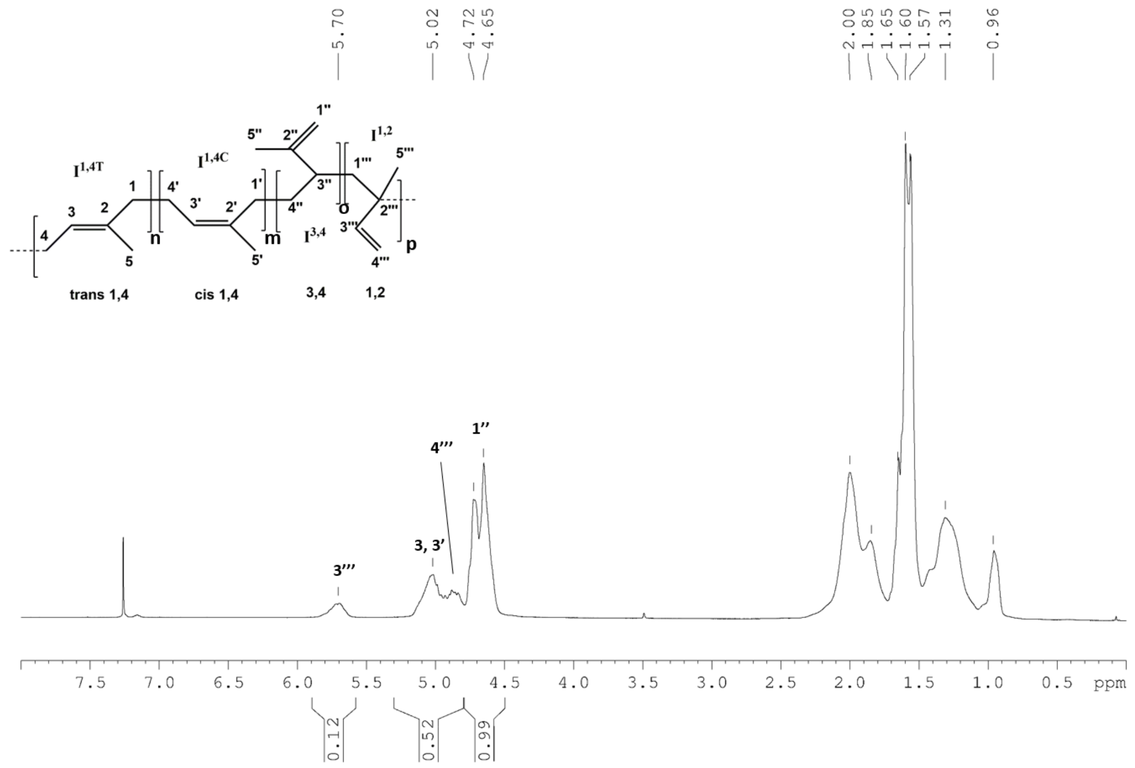

Figure S8. ^{1}H NMR spectrum (TCDE, 25 °C, 400 MHz) of PI from run 7 of **Table 1**

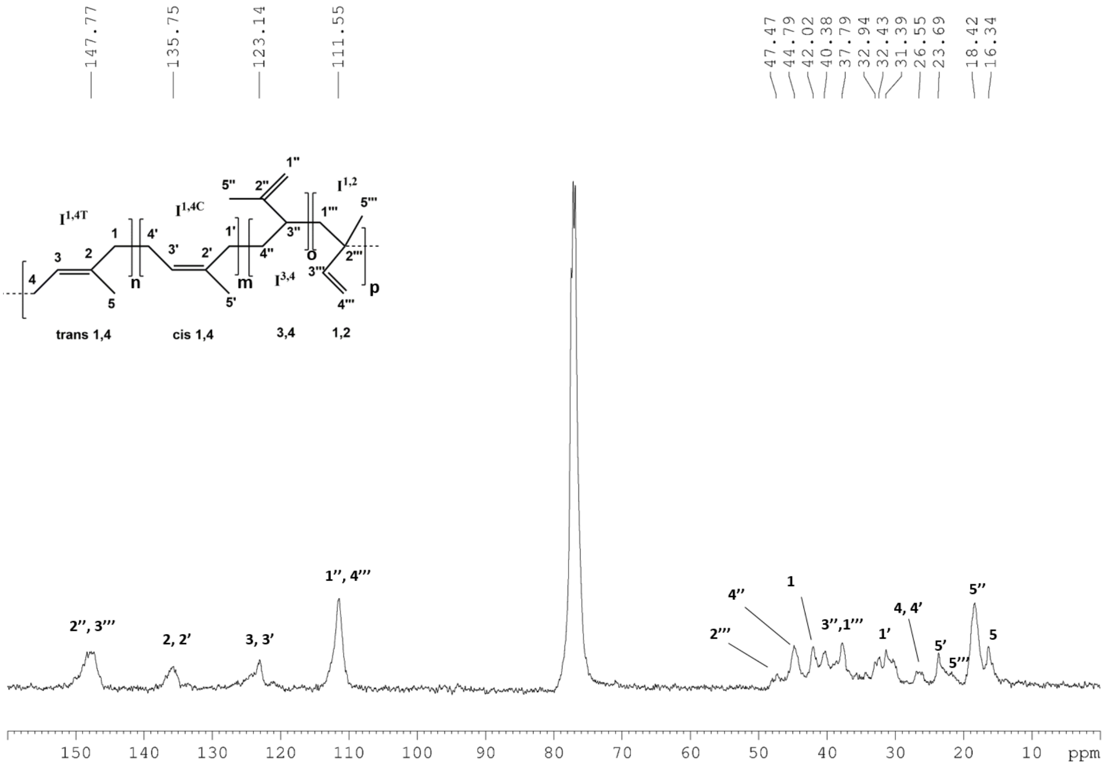

Figure S9. ^{13}C NMR spectrum (TCDE, 25 °C, 400 MHz) of PI from run 7 of **Table 1**

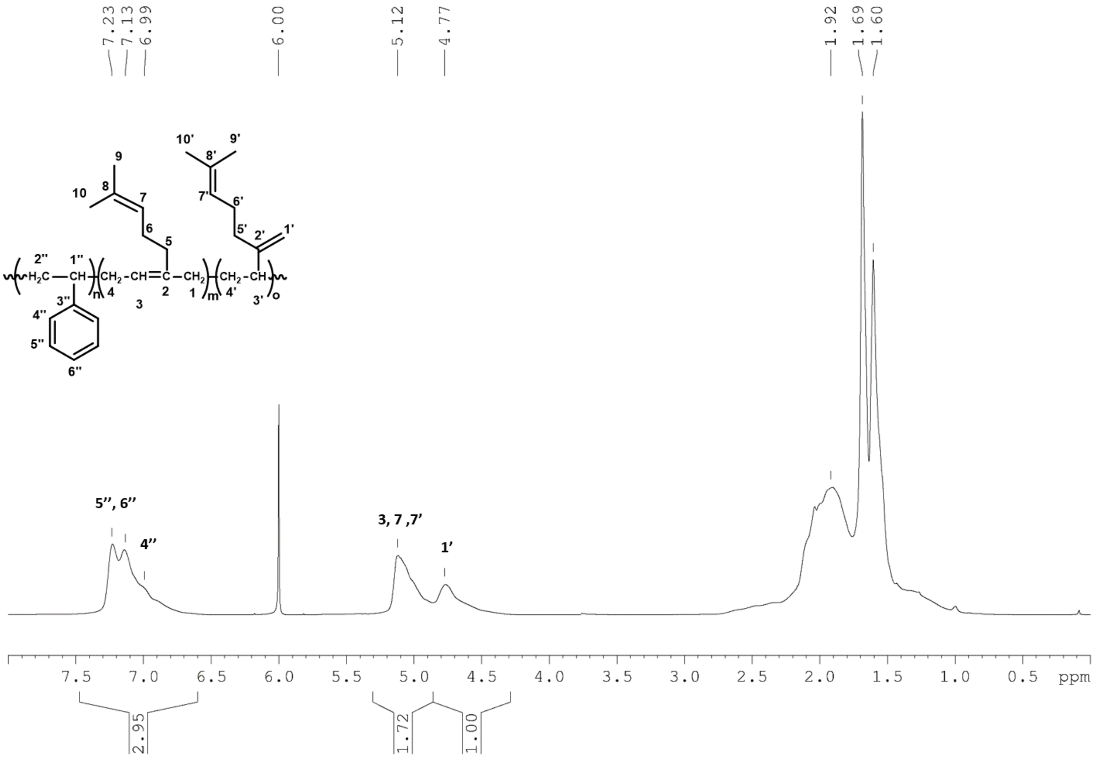

Figure S10. ^{1}H NMR spectrum (TCDE, 25 °C, 400 MHz) of PMS from run 12 of **Table 2**

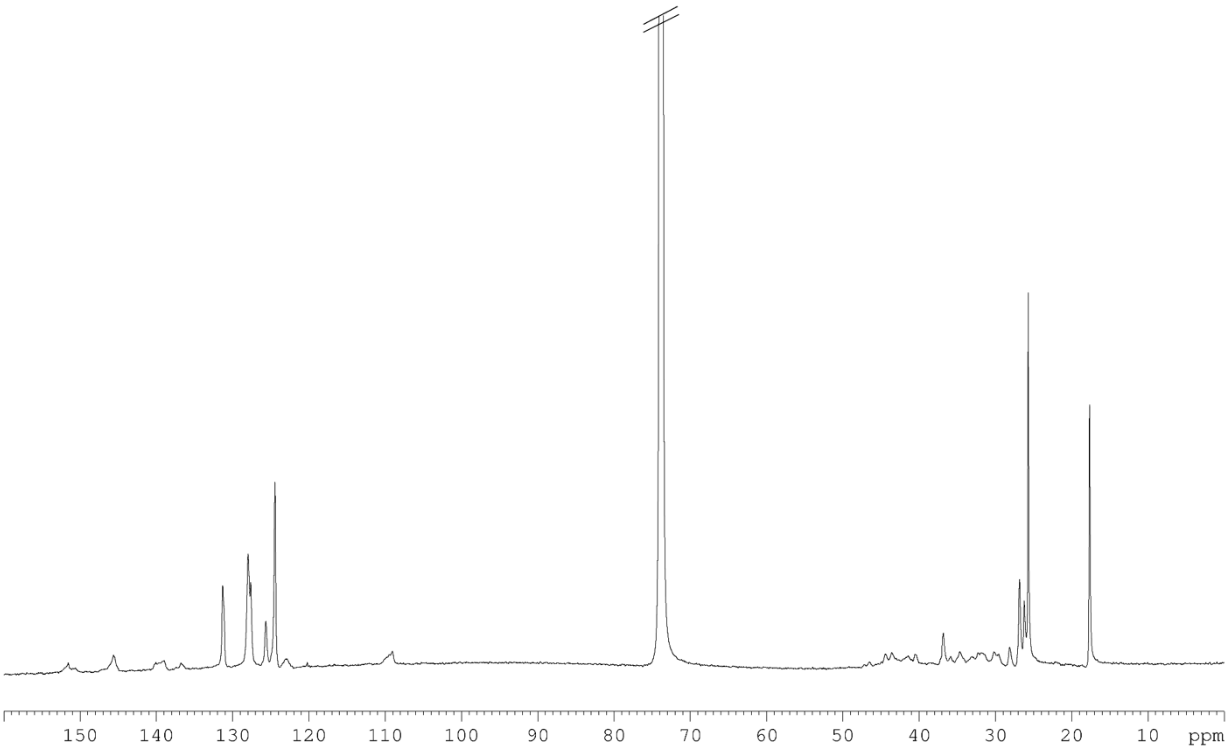

Figure S11. ^{13}C NMR spectrum (TCDE, 25 °C, 400 MHz) of PMS from run 12 of **Table 2**

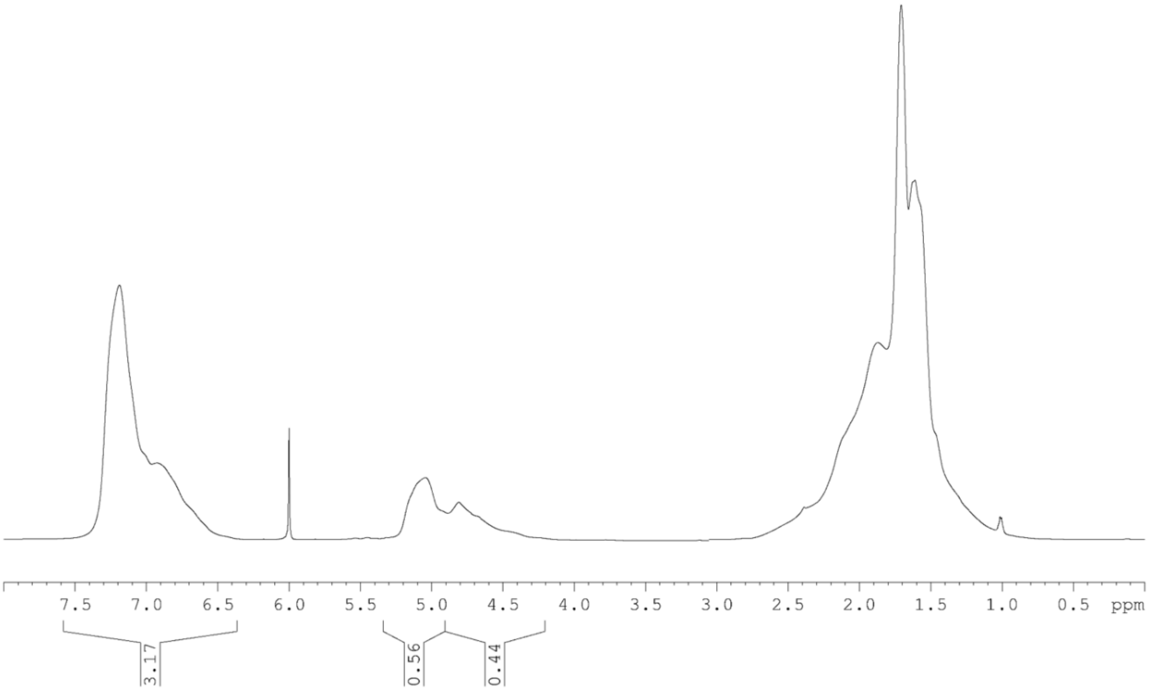

Figure S12. ^{1}H NMR spectrum (TCDE, 25 °C, 400 MHz) of PMS from run 13 of **Table 2**

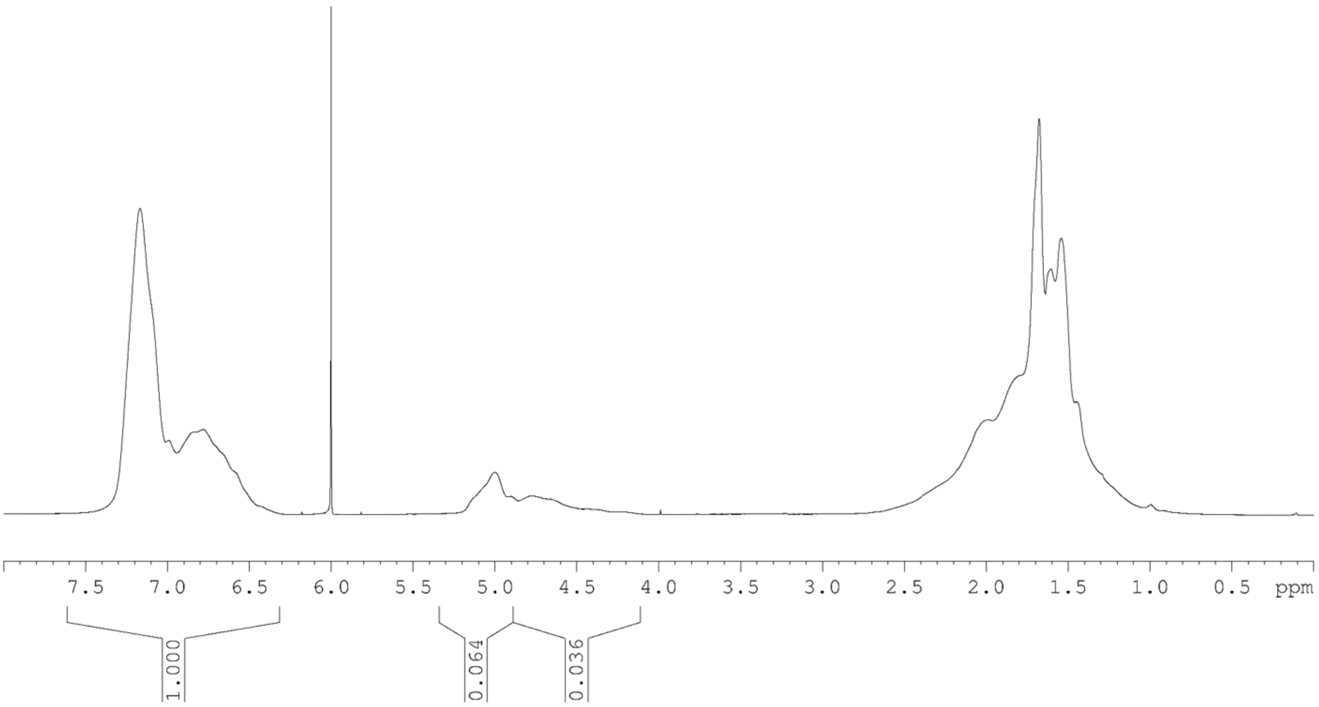

Figure S13. ^{1}H NMR spectrum (TCDE, 25 °C, 400 MHz) of PMS from run 14 of **Table 2**

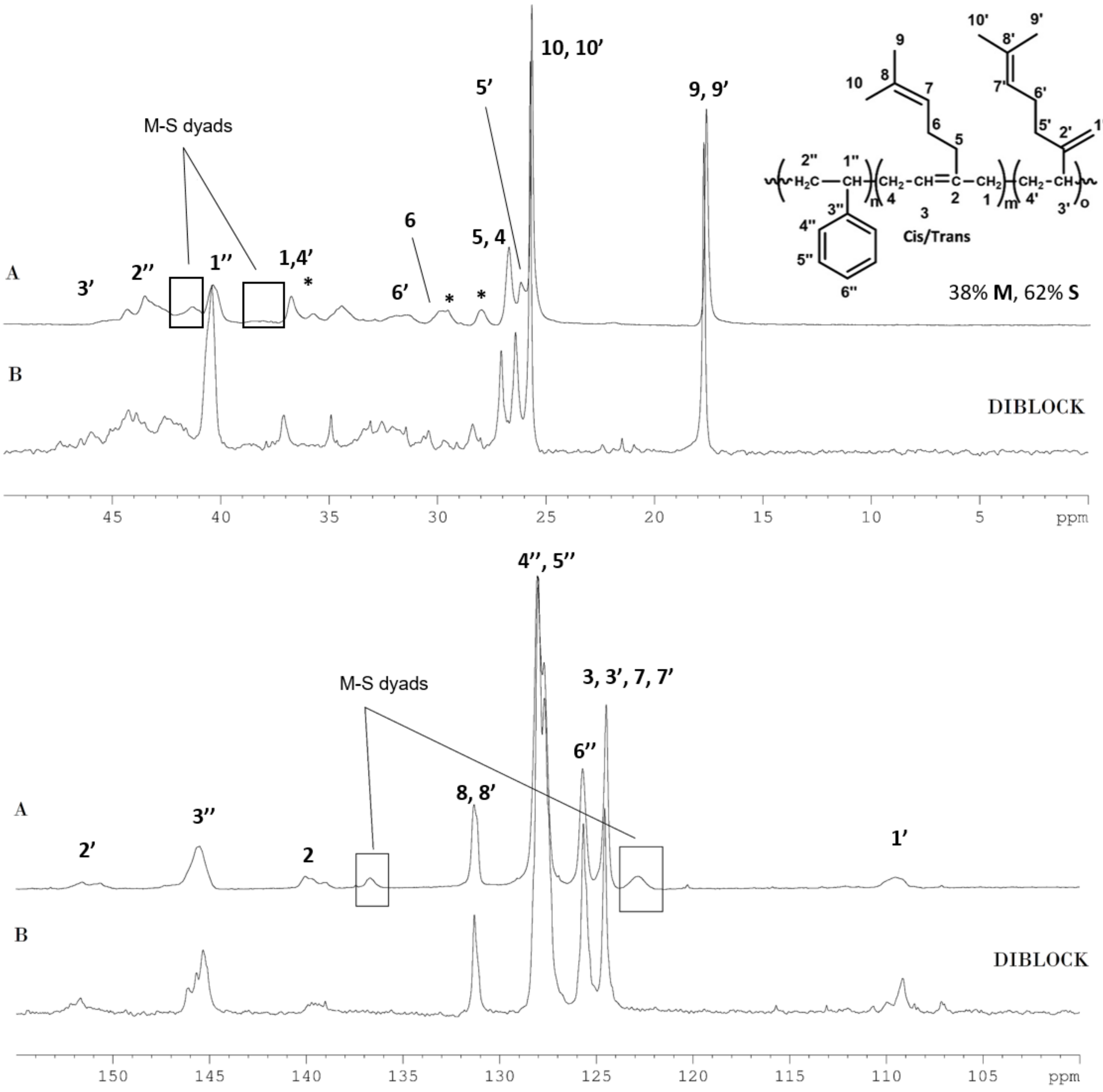

Figure S14. ^{13}C NMR spectra (TCDE, 25 °C, 400 MHz) of PMS copolymer from run 13 (a) and PMS diblock from run 21 (b) of **Table 2**

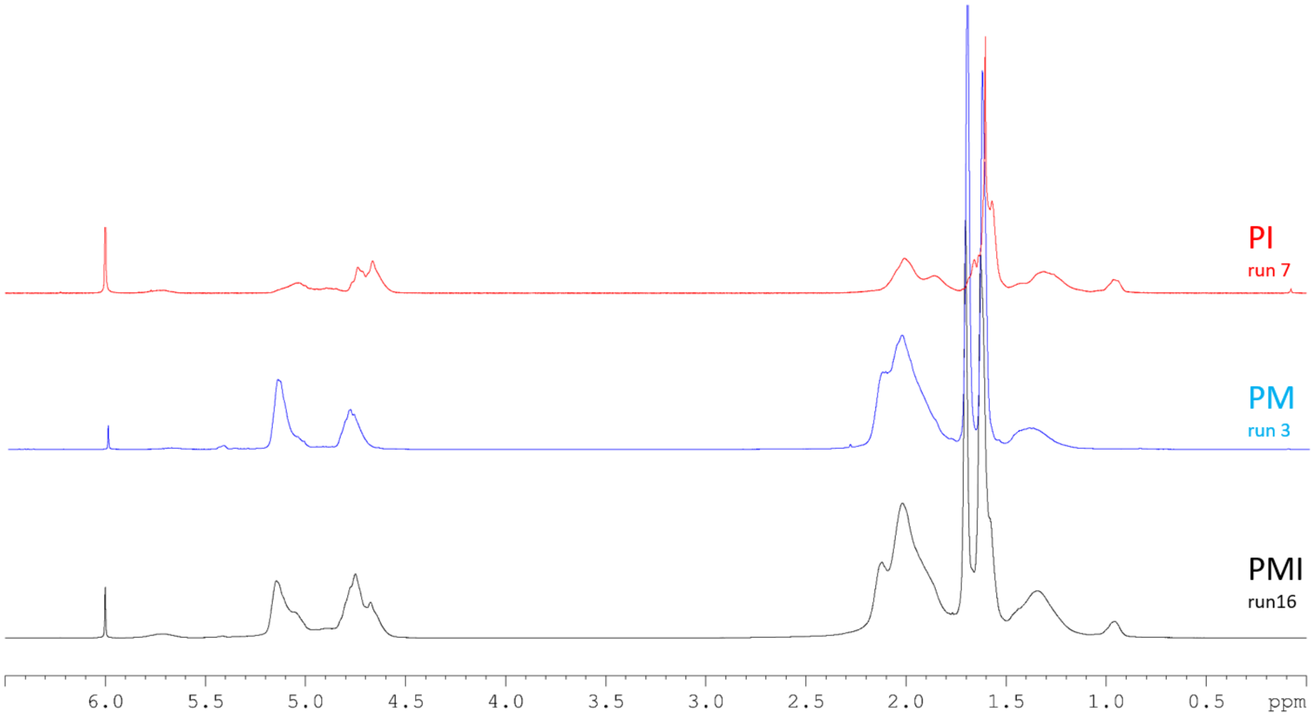

Figure S15. ^{1}H NMR spectra (TCDE, 25 °C, 400 MHz) of PM (run 3), PI (run 7) and PMI (run 16) from **Table 2**

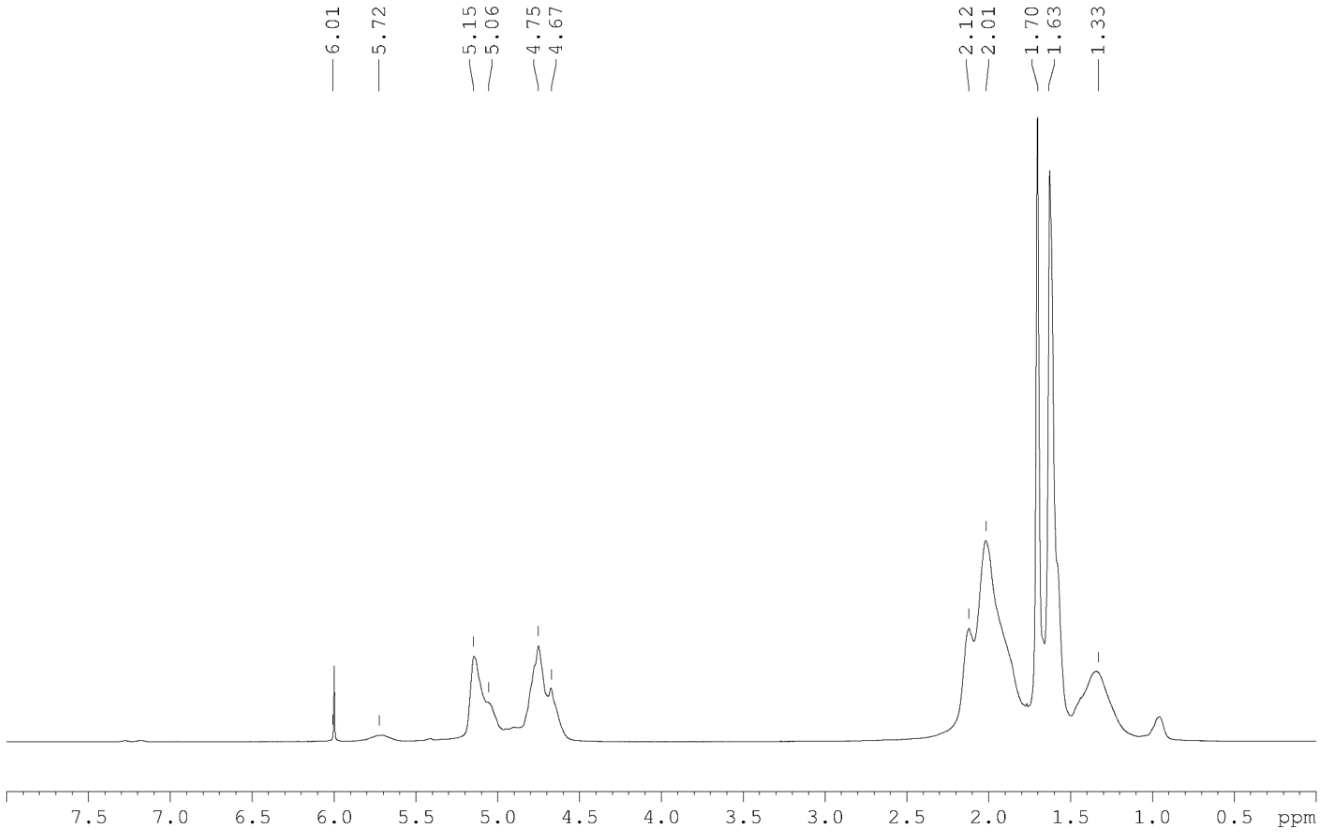

Figure S16. ^{1}H NMR spectrum (TCDE, 25 °C, 400 MHz) of PMI from run 16 of **Table 2**

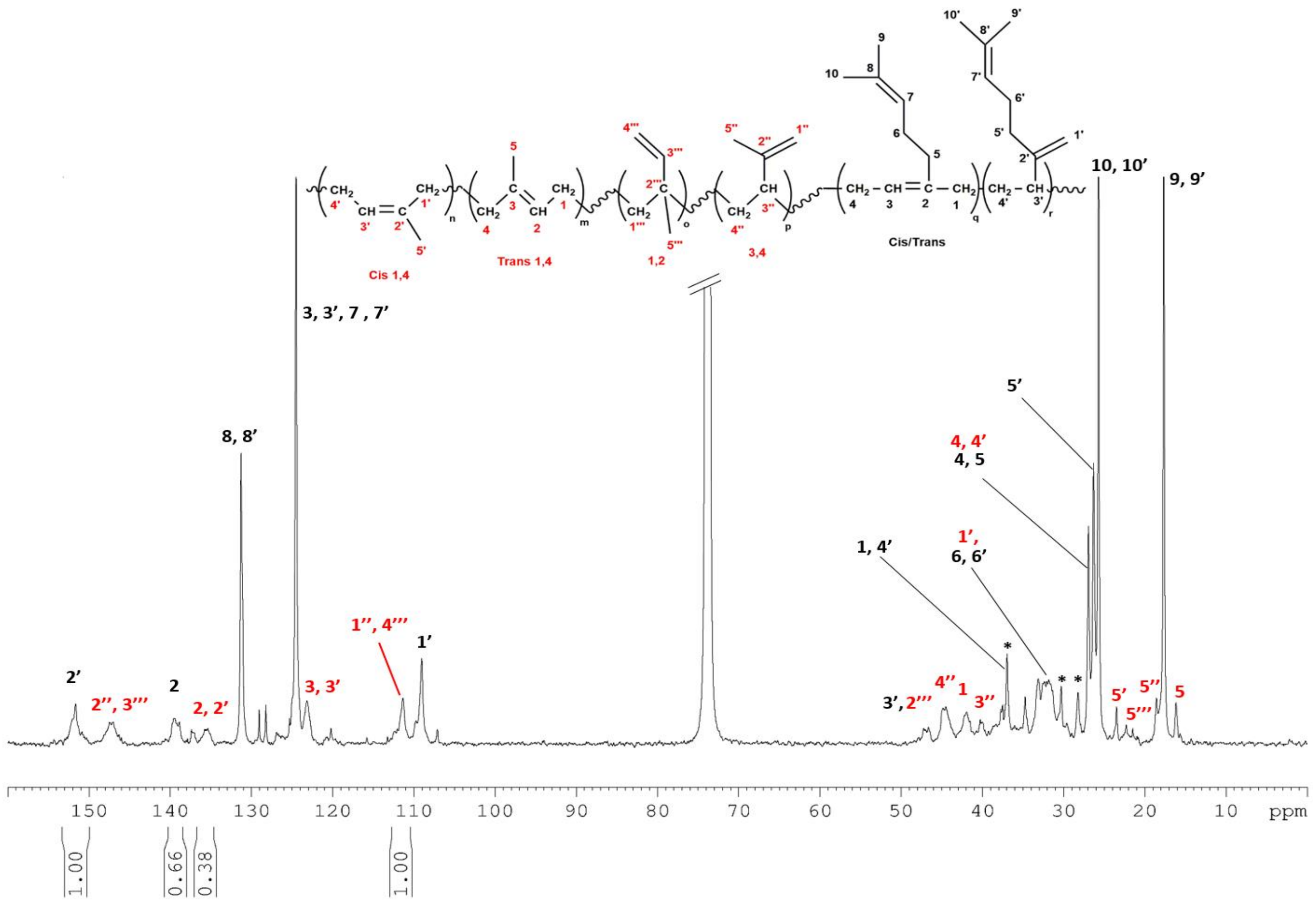

Figure S17. ^{13}C NMR spectrum (TCDE, 25 °C, 400 MHz) of PMI from run 16 of **Table 2**

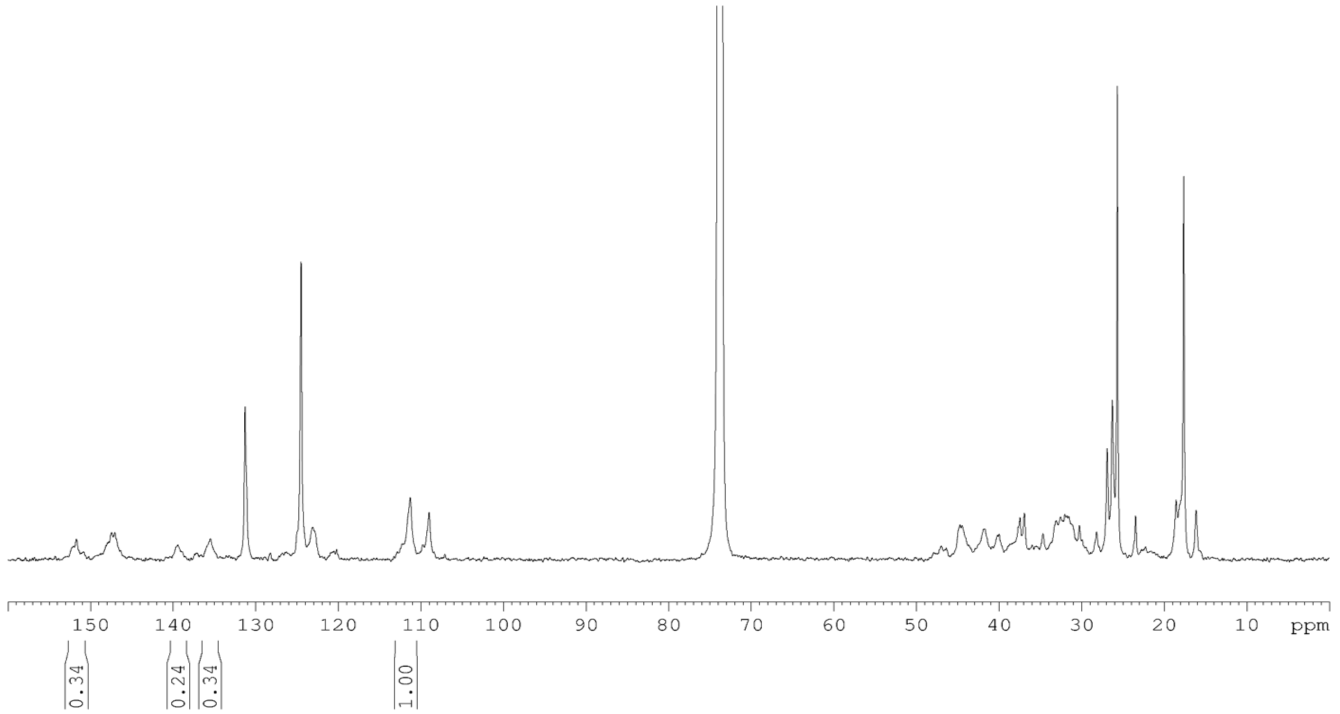

Figure S18. ^{13}C NMR spectrum (TCDE, 25 °C, 400 MHz) of PMI from run 17 of **Table 2**

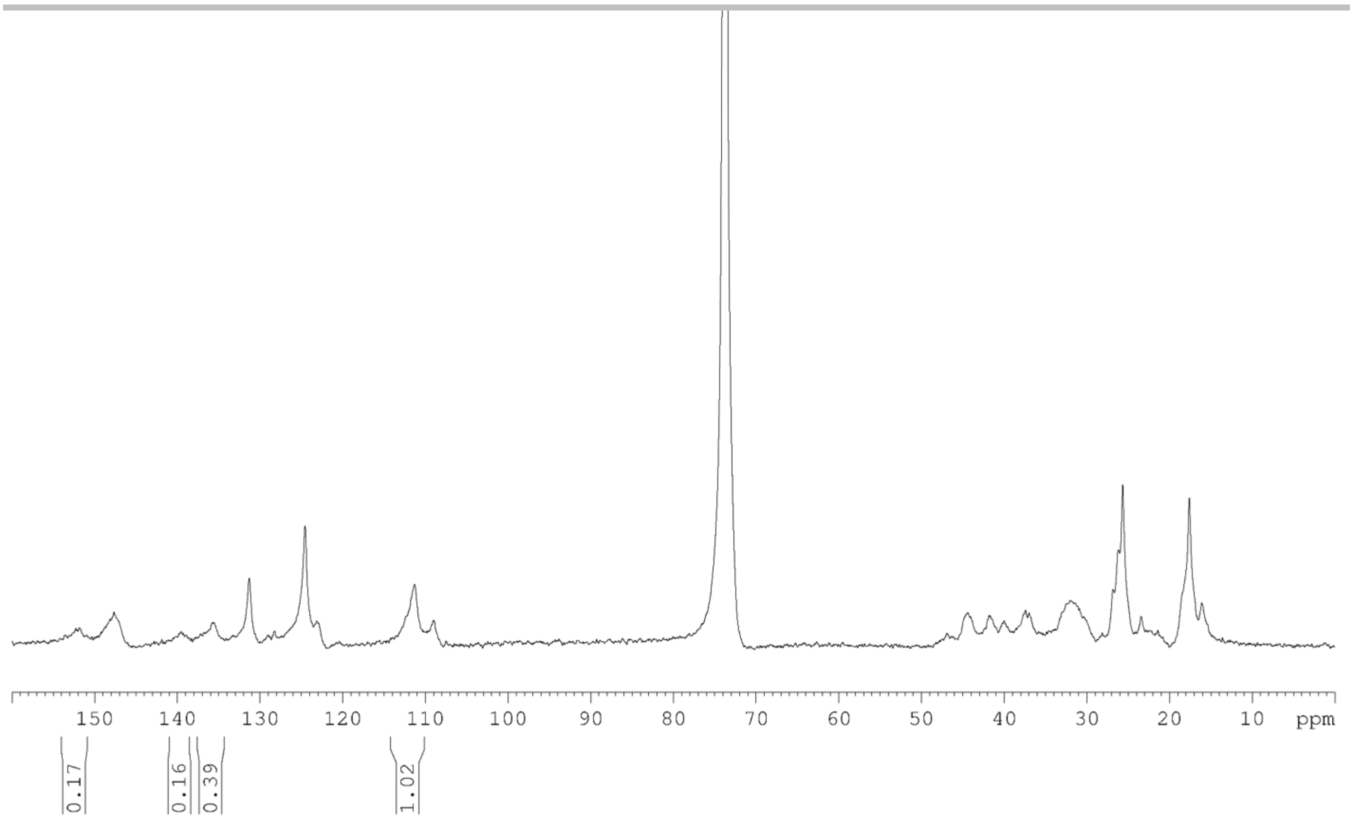

Figure S19. ^{13}C NMR spectrum (TCDE, 25 °C, 400 MHz) of PMI from run 18 of **Table 2**

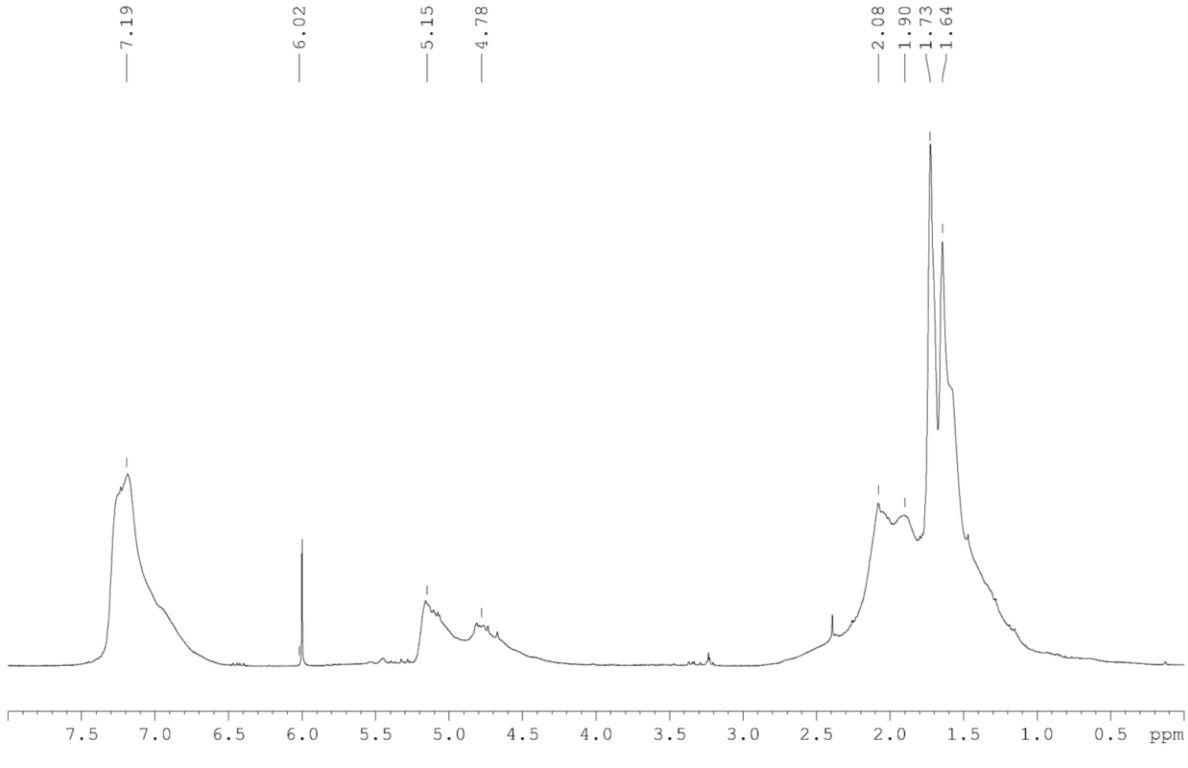

Figure S20. ^{1}H NMR spectrum (TCDE, 25 °C, 400 MHz) of PMSI from run 18 of **Table 2**

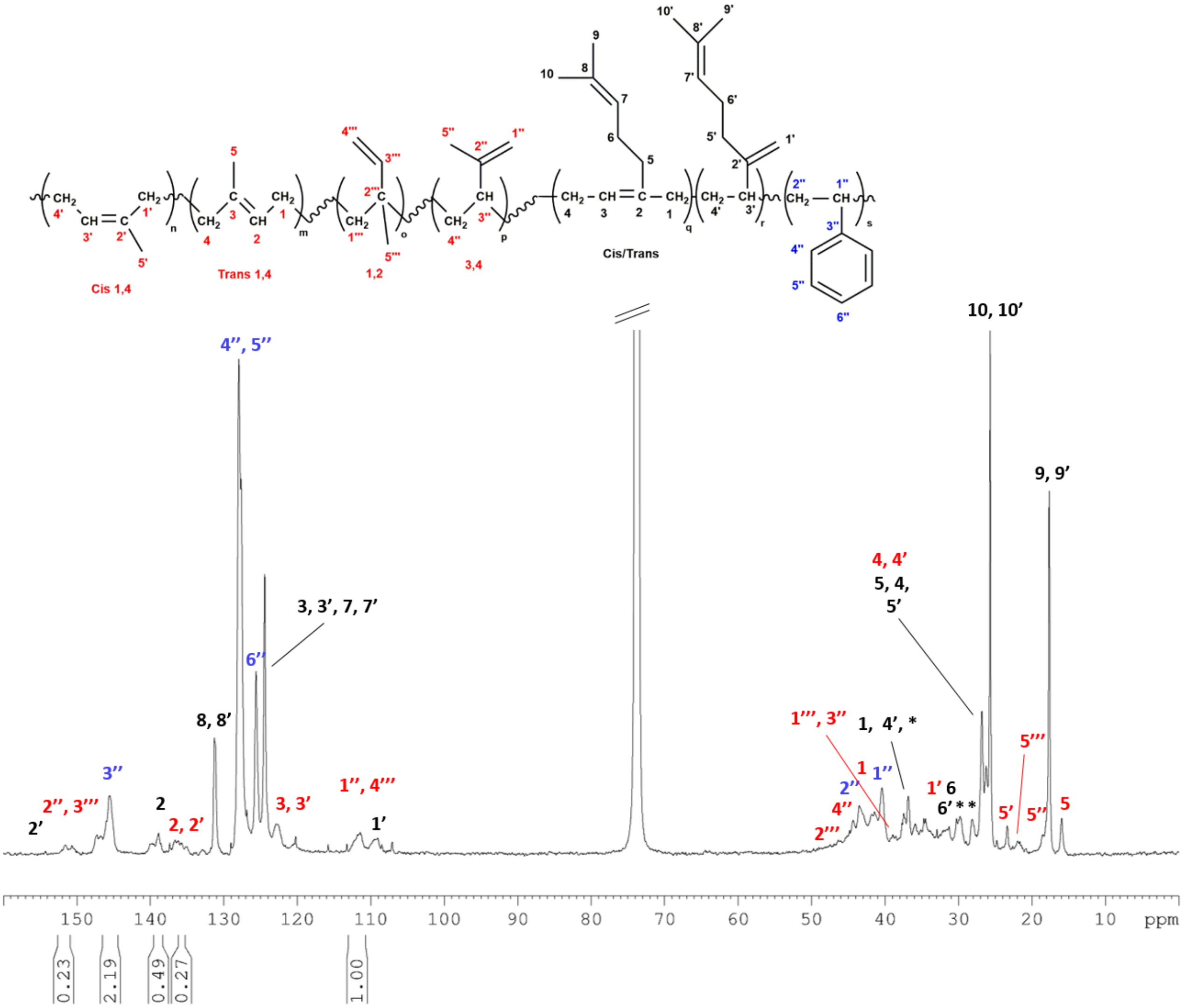

Figure S21. ^{13}C NMR spectrum (TCDE, 25 °C, 400 MHz) of PMSI from run 18 of **Table 2**

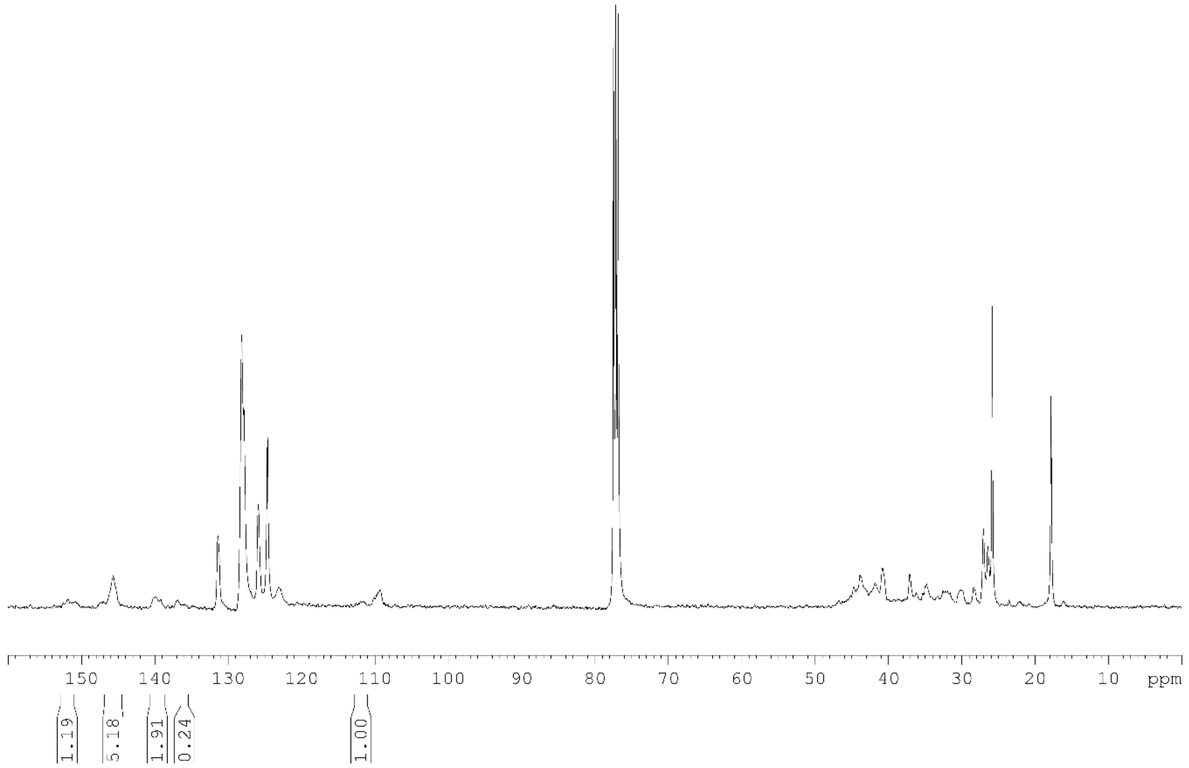

Figure S22. ^{13}C NMR spectrum ($CDCl_3$, 25 °C, 400 MHz) of PMSI from run 19 of **Table 2**

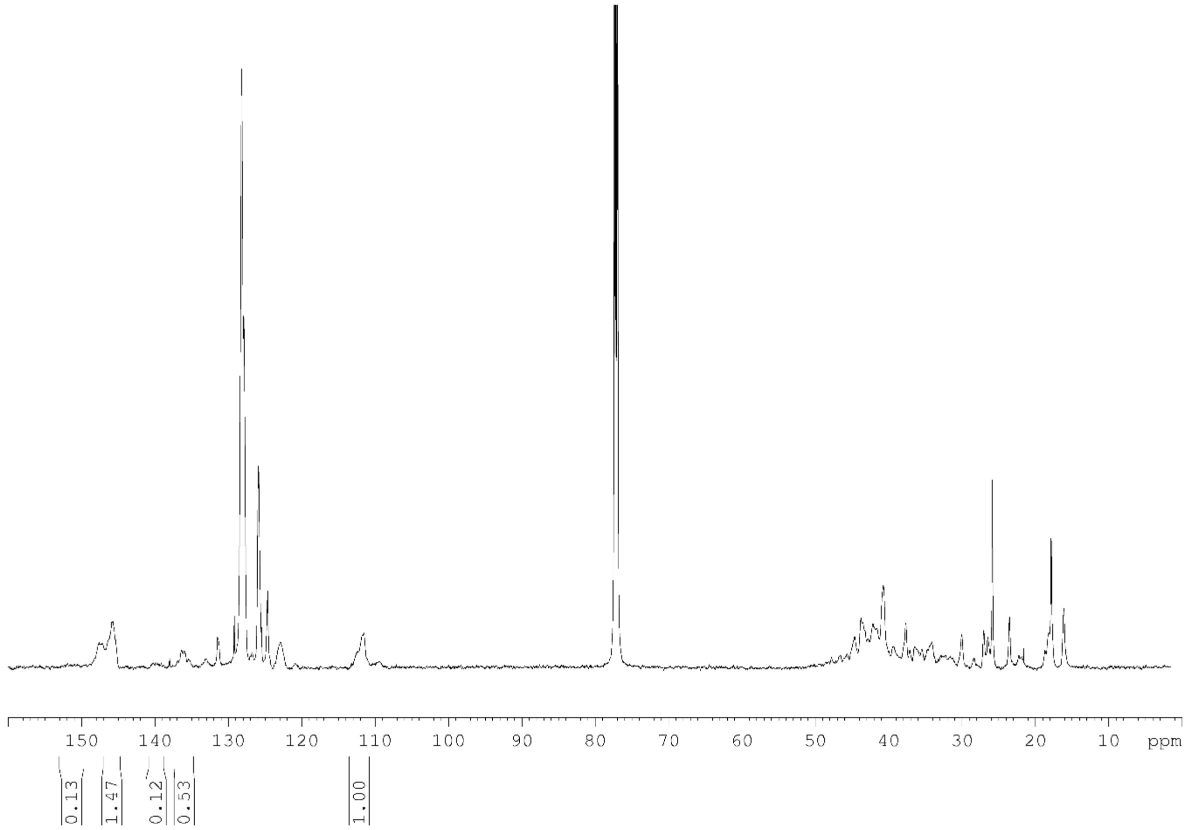

Figure S23. ^{13}C NMR spectrum (TCDE, 25 °C, 400 MHz) of PMSI from run 20 of **Table 2**

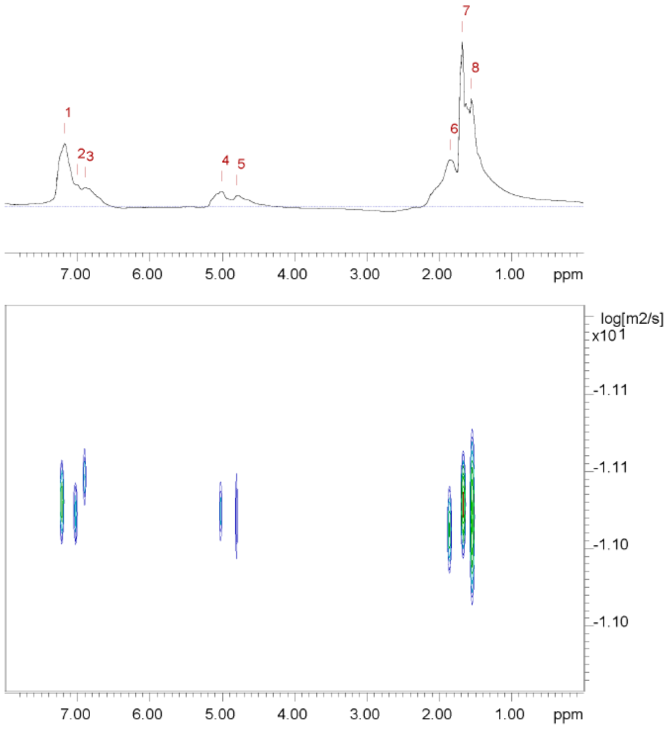

Fitted function:	f (x) = A * exp (-D * x^2 * gamma^2 * littleDelta^2 (bigDelta-littleDelta/3)* 10^4
used gamma:	26752 rad/(s*Gauss)
used little delta:	0.0050000 s
used big delta:	0.14990 s
used gradient strength:	variable
Random error estimation of data:	RMS per spectrum (or trace/plane)
Systematic error estimation of data:	worst case per peak scenario
Fit parameter Error estimation method:	from fit using arbitray y uncertainties
Confidence level:	95%
Used peaks:	automatically picked peaks
Used integrals:	peak intensities
Used Gradient strength:	all values (including replicates) used

Peak name	F2 [ppm]	D [m2/s]	error
1	7.190	8.09e-12	3.034e-13
2	7.008	8.28e-12	3.385e-13
3	6.889	7.89e-12	2.786e-13
4	5.013	8.62e-12	3.627e-13
5	4.795	9.39e-12	7.118e-13
6	1.854	8.84e-12	3.726e-13
7	1.683	8.57e-12	3.458e-13
8	1.558	8.74e-12	6.227e-13

Figure S24. DOSY NMR spectrum ($CDCl_3$, 25 °C, 600 MHz) of PMS copolymers from run 13 of **Table 2**

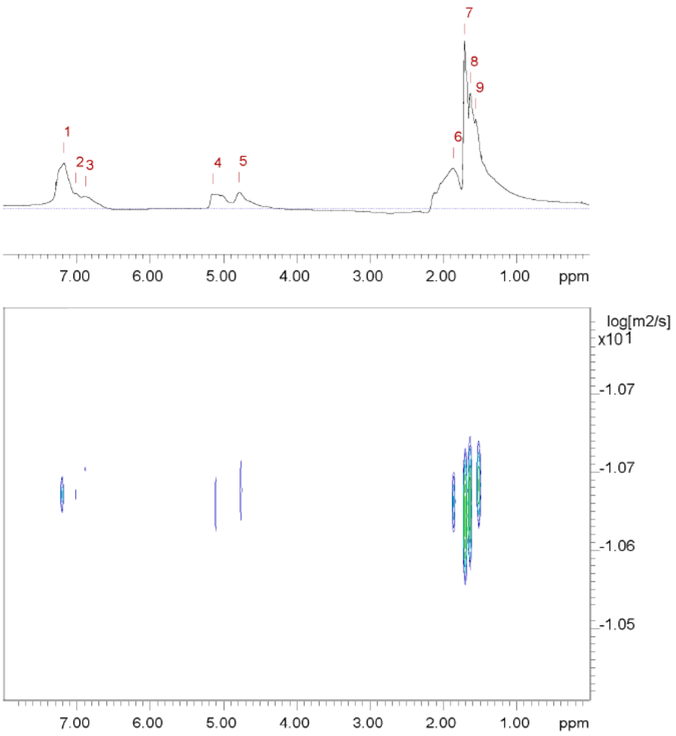

Fitted function:	f (x) = A * exp (-D * x^2 * gamma^2 * littleDelta^2 (bigDelta-littleDelta/3)* 10^4
used gamma:	26752 rad/(s*Gauss)
used little delta:	0.0050000 s
used big delta:	0.14990 s
used gradient strength:	variable
Random error estimation of data:	RMS per spectrum (or trace/plane)
Systematic error estimation of data:	worst case per peak scenario
Fit parameter Error estimation method:	from fit using arbitray y uncertainties
Confidence level:	95%
Used peaks:	automatically picked peaks
Used integrals:	peak intensities
Used Gradient strength:	all values (including replicates) used

Peak name	F2 [ppm]	D [m2/s]	error
1	7.190	2.05e-11	6.259e-13
2	7.018	2.05e-11	5.955e-13
3	6.883	1.97e-11	3.899e-13
4	5.138	2.97e-11	3.340e-12
5	4.790	2.79e-11	2.901e-12
6	1.859	2.29e-11	1.212e-12
7	1.709	2.62e-11	2.167e-12
8	1.636	2.59e-11	2.299e-12
9	1.558	2.20e-11	1.393e-12

Figure S25. DOSY NMR spectrum ($CDCl_3$, 25 °C, 600 MHz) of PMS diblock copolymers from run of 21 **Table 2**

4. DSC Thermal Analysis

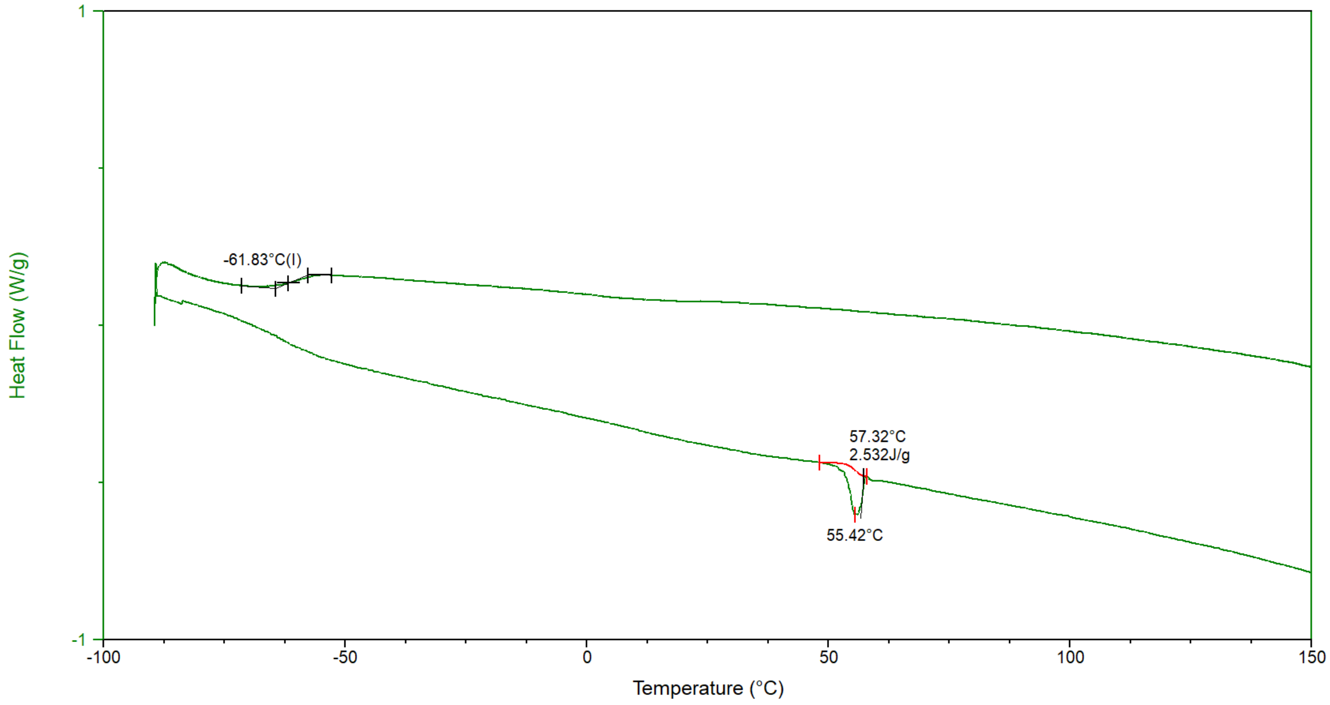

Figure S26. DSC thermogram of PM from run 1 of **Table 1**.

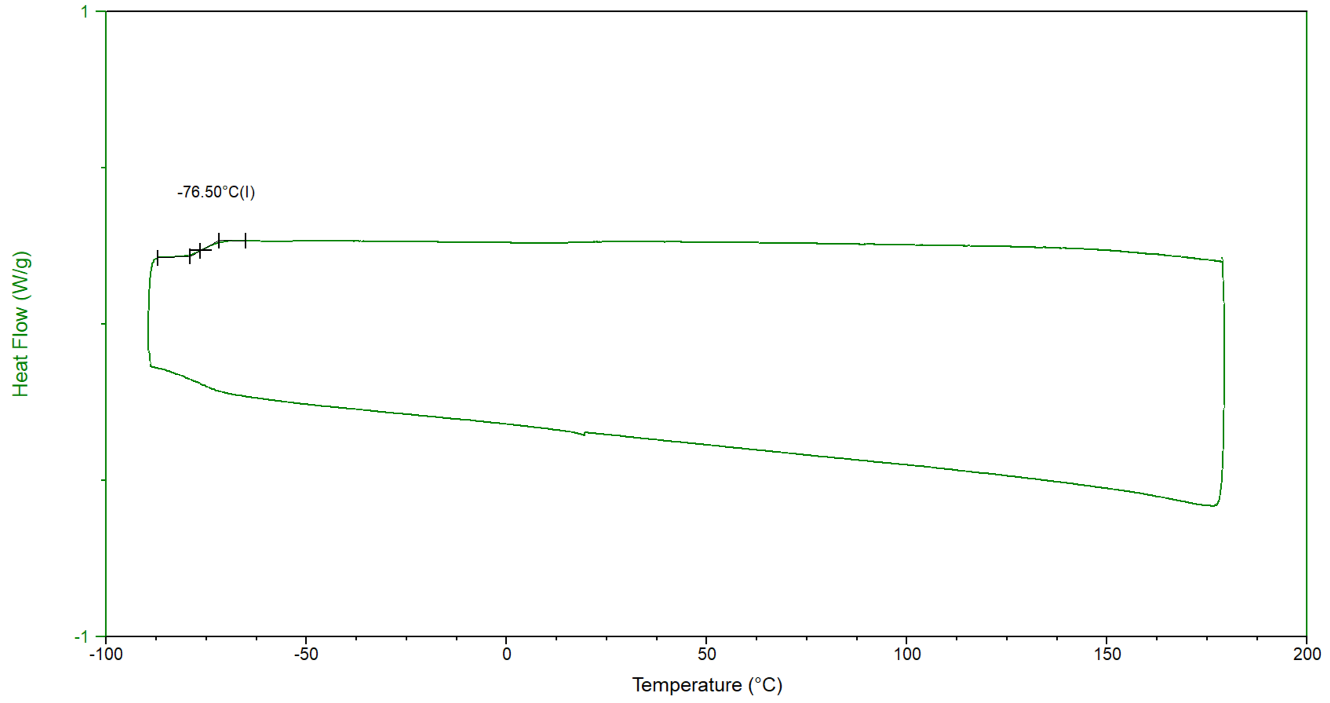

Figure S27. DSC thermogram of PM from run 4 of **Table 1**.

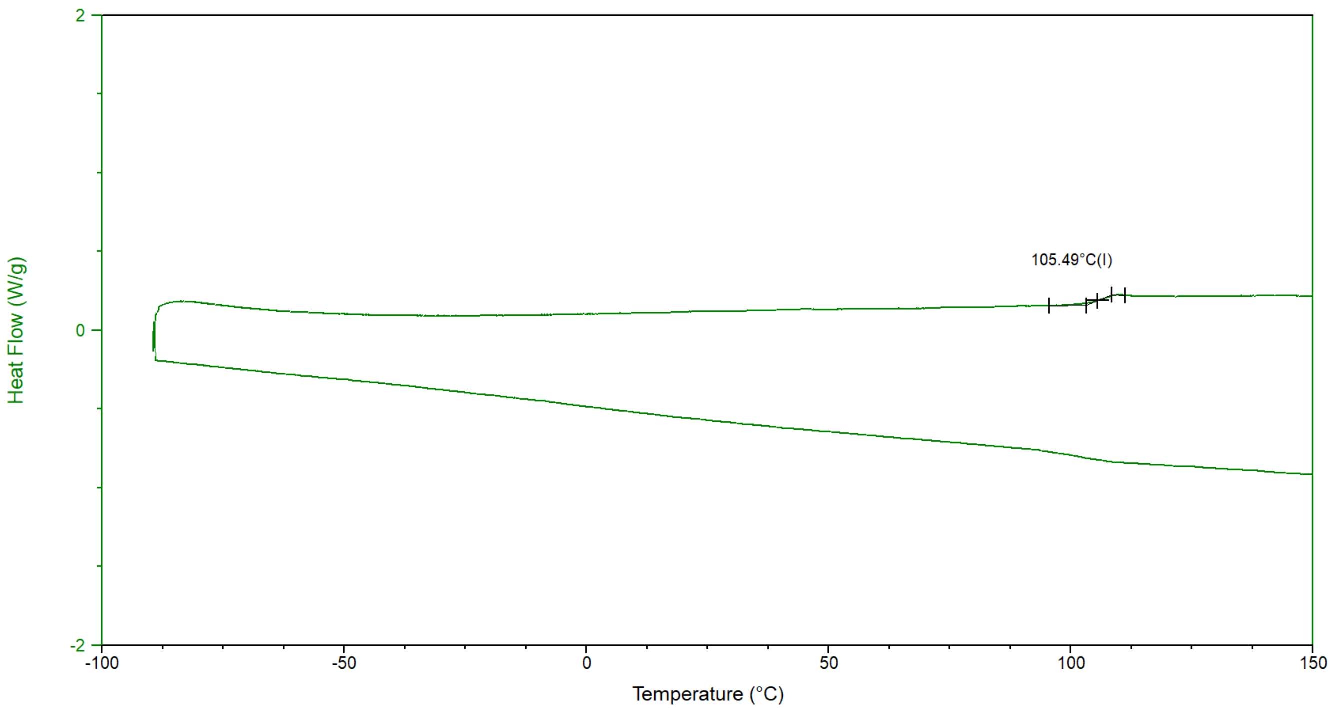

Figure S28. DSC thermogram of PS from run 5 of **Table 1**.

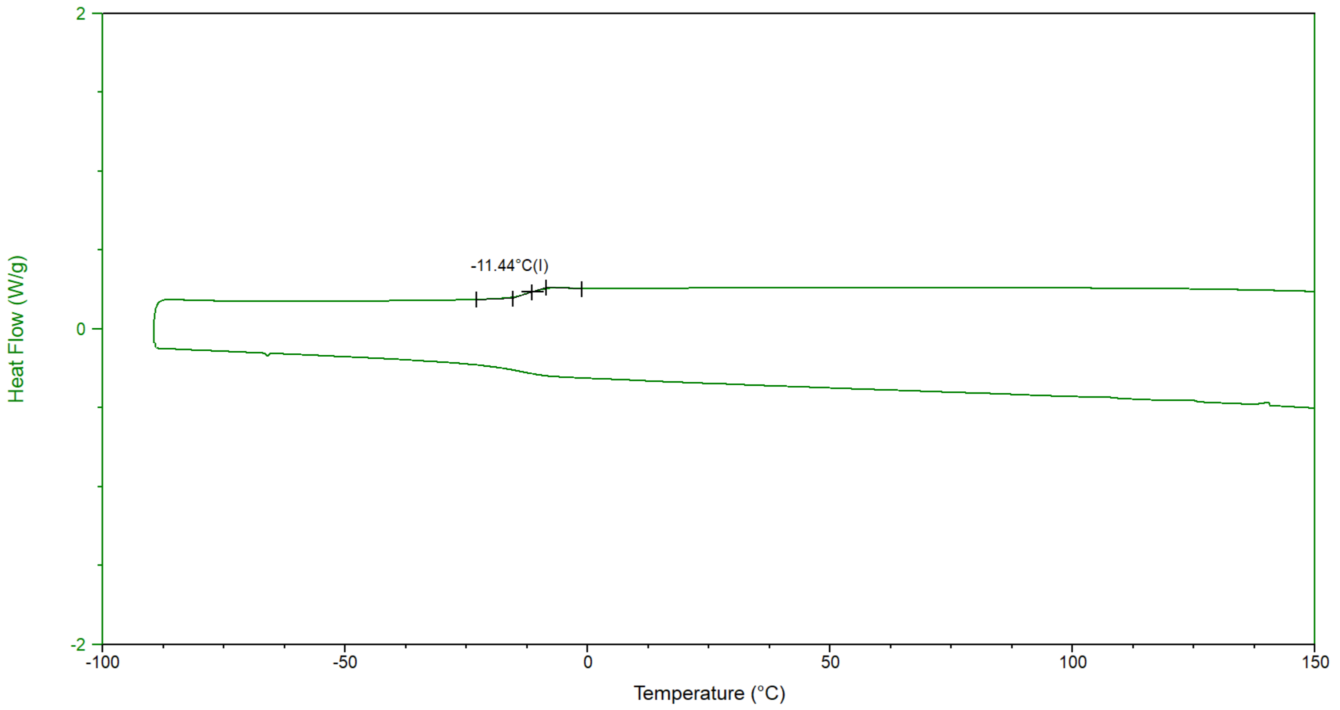

Figure S29. DSC thermogram of PI from run 8 of **Table 1**.

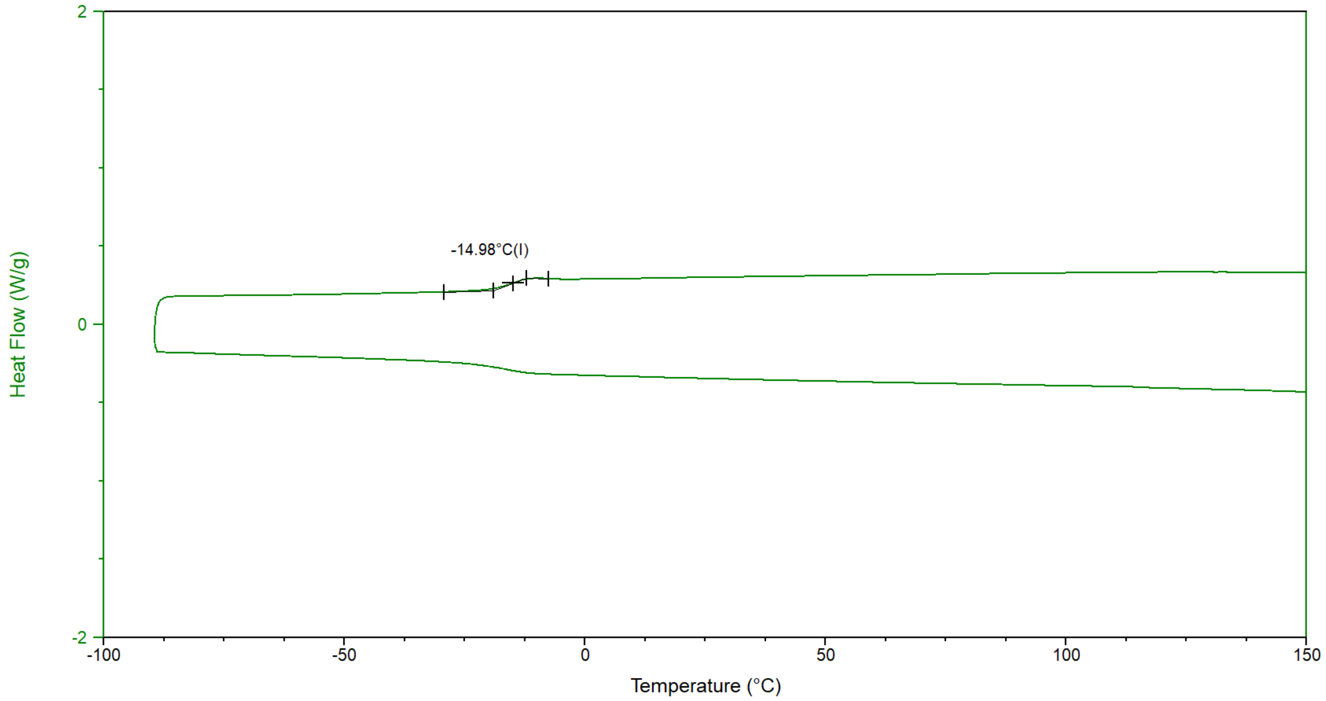

Figure S30. DSC thermogram of PI from run 9 of **Table 1**.

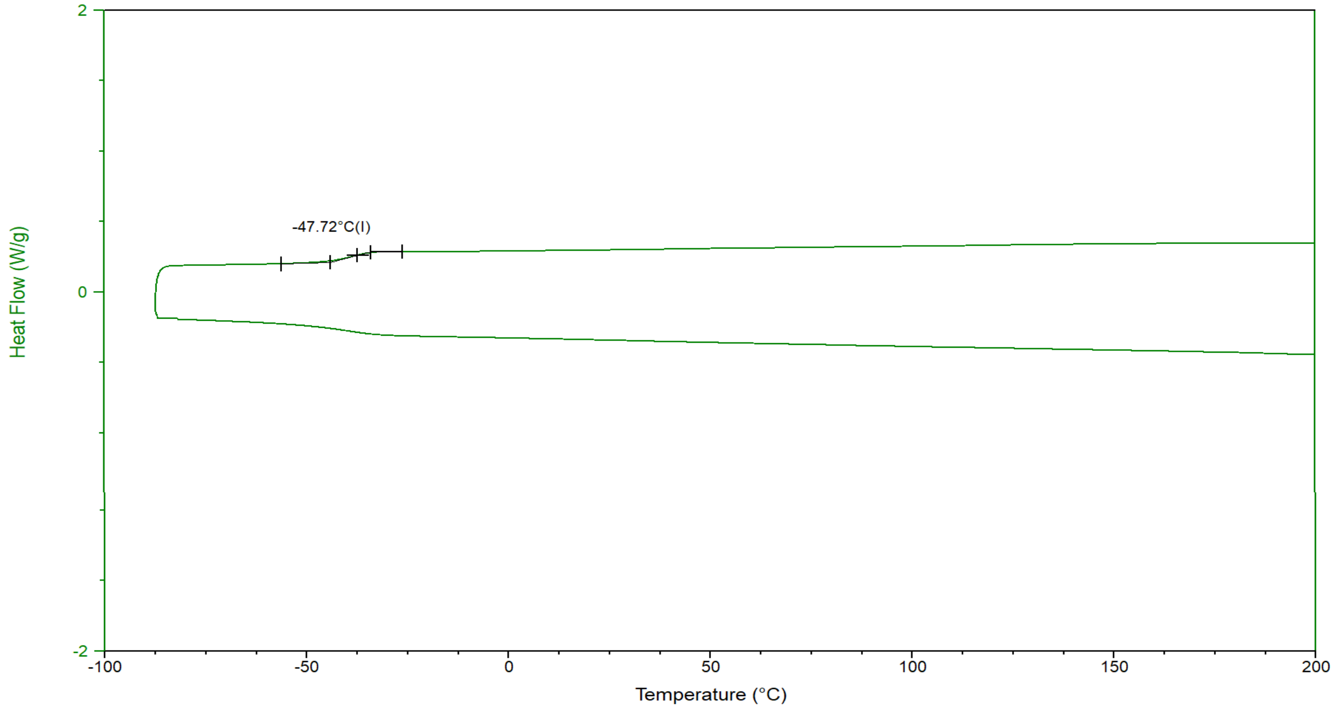

Figure S31. DSC thermogram of PMS from run 10 of **Table 2**.

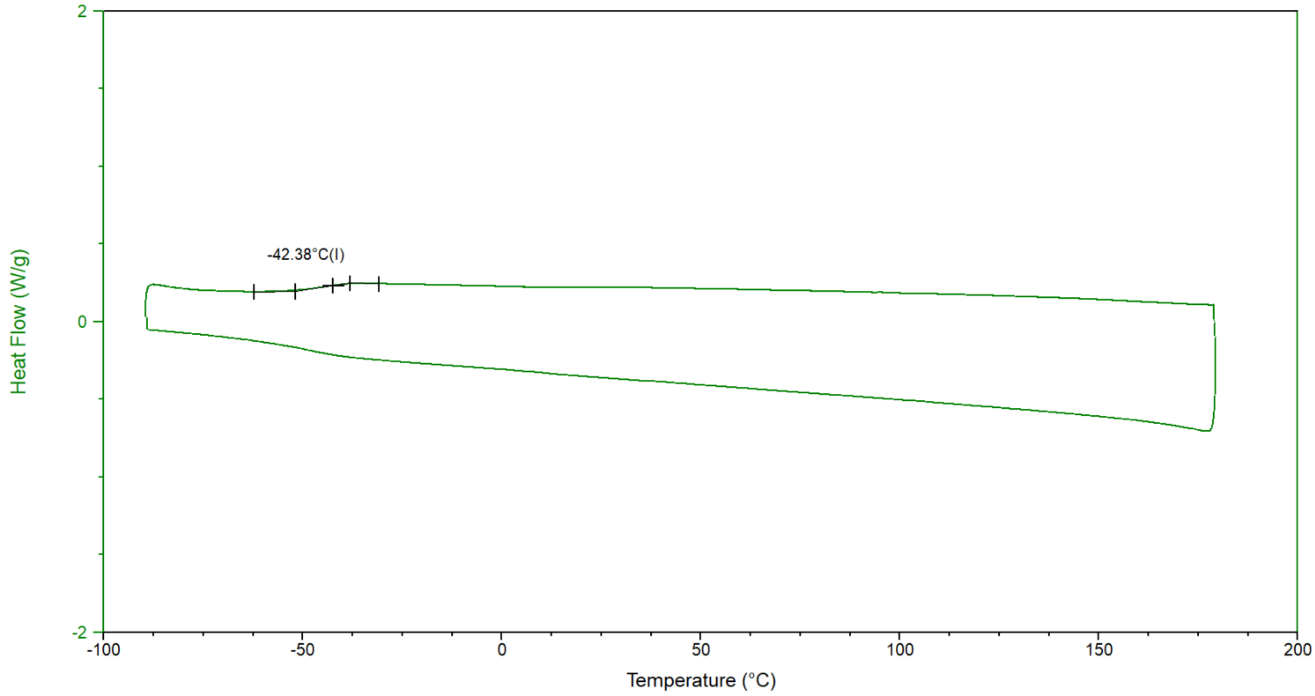

Figure S32. DSC thermogram of PMS from run 12 of **Table 2**.

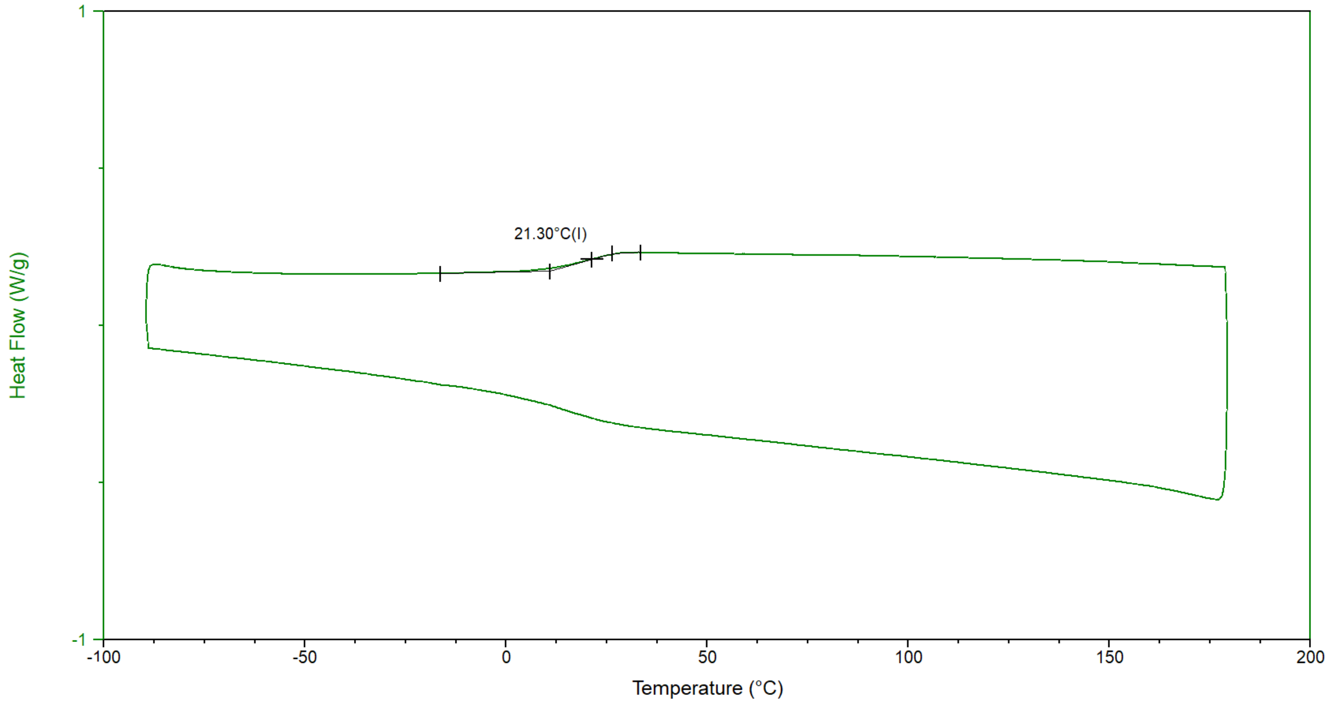

Figure S33. DSC thermogram of PMS from run 14 of **Table 2**.

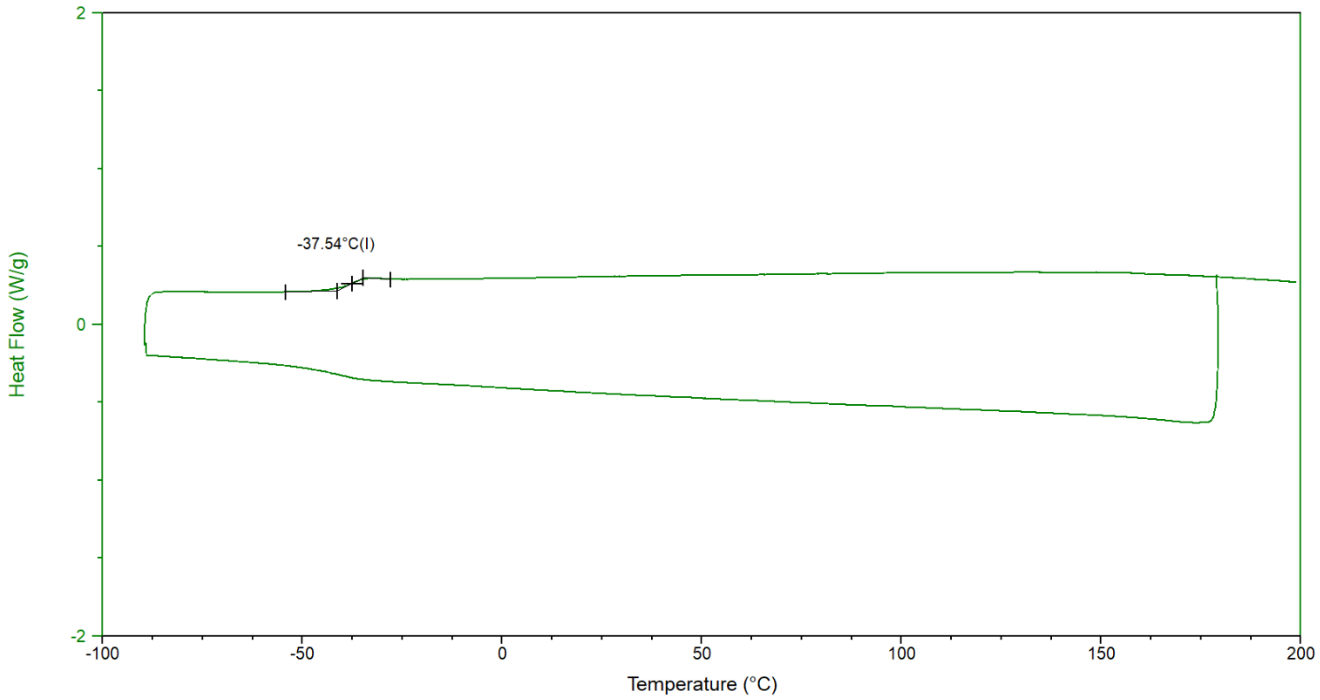

Figure S34. DSC thermogram of PMI from run 16 of **Table 2**.

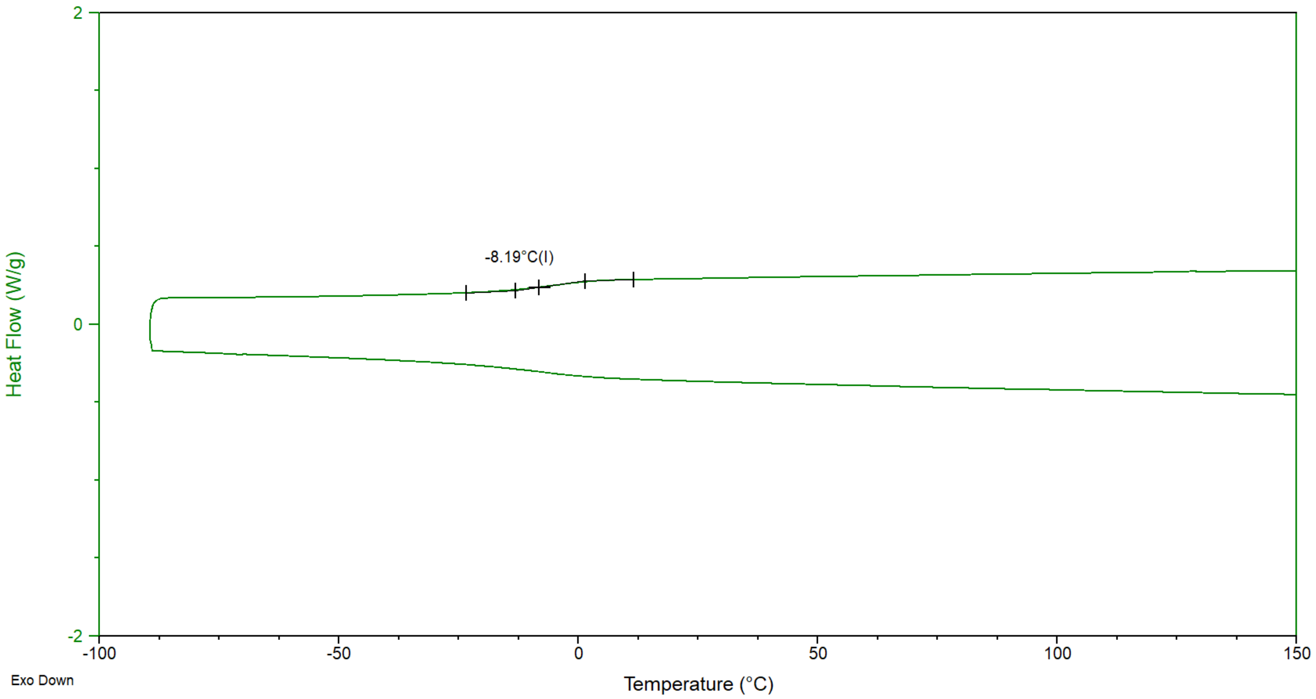

Figure S35. DSC thermogram of PMSI from run 18 of **Table 2**.

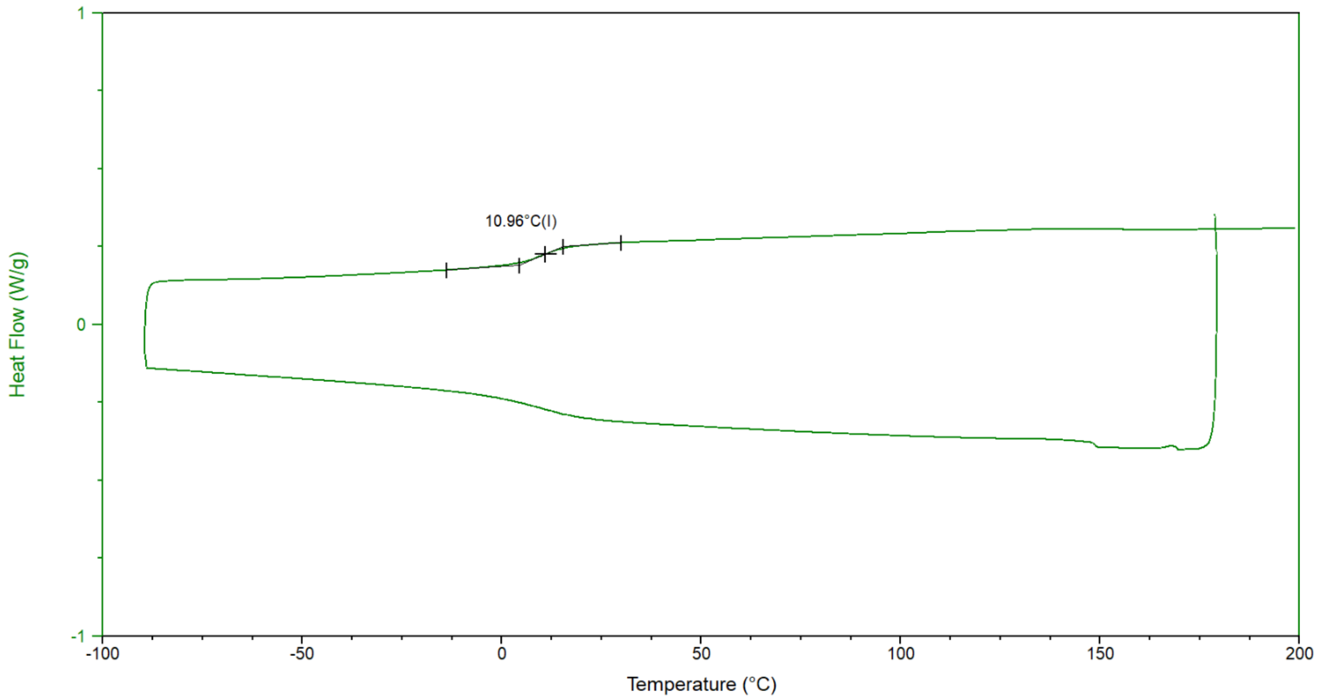

Figure S36. DSC thermogram of PMSI from run 20 of **Table 2**.

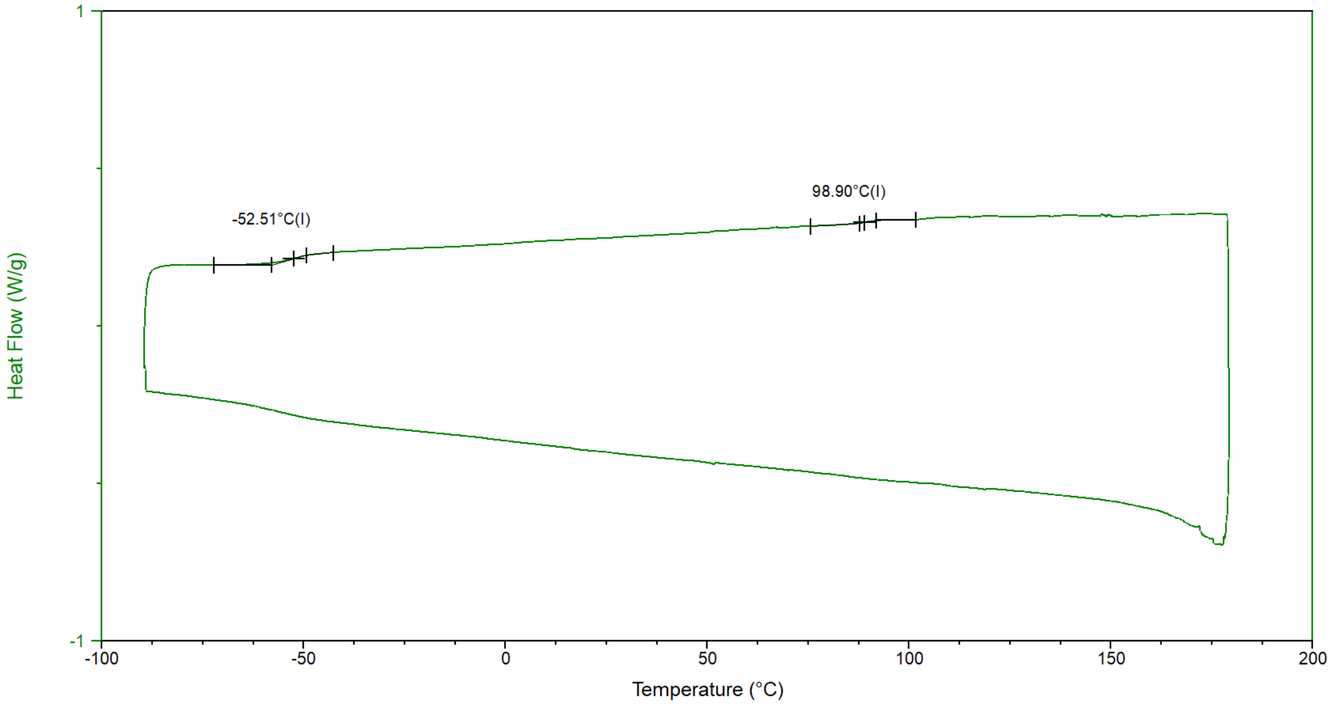

Figure S37. DSC thermogram of diblock PMS from run 21 of **Table 2**.

5. TGA Analysis

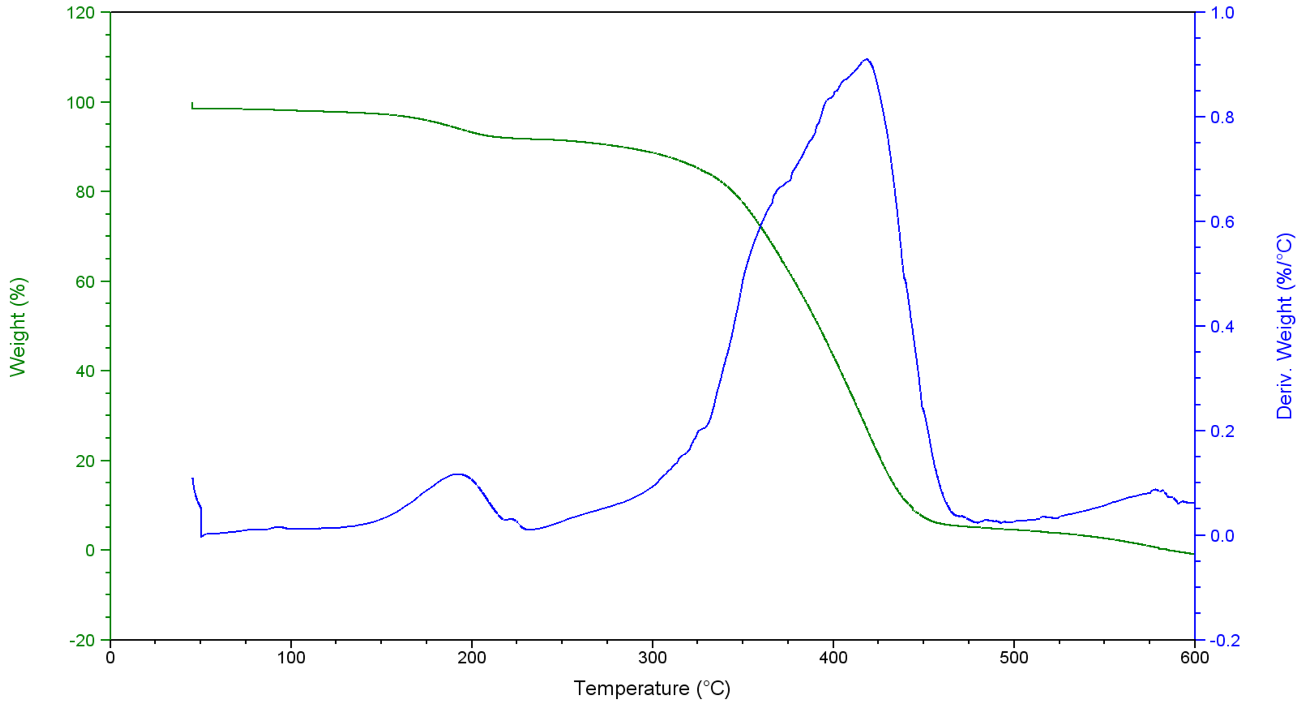

Figure S38. TGA thermogram of PM from run 1 of **Table 1**.

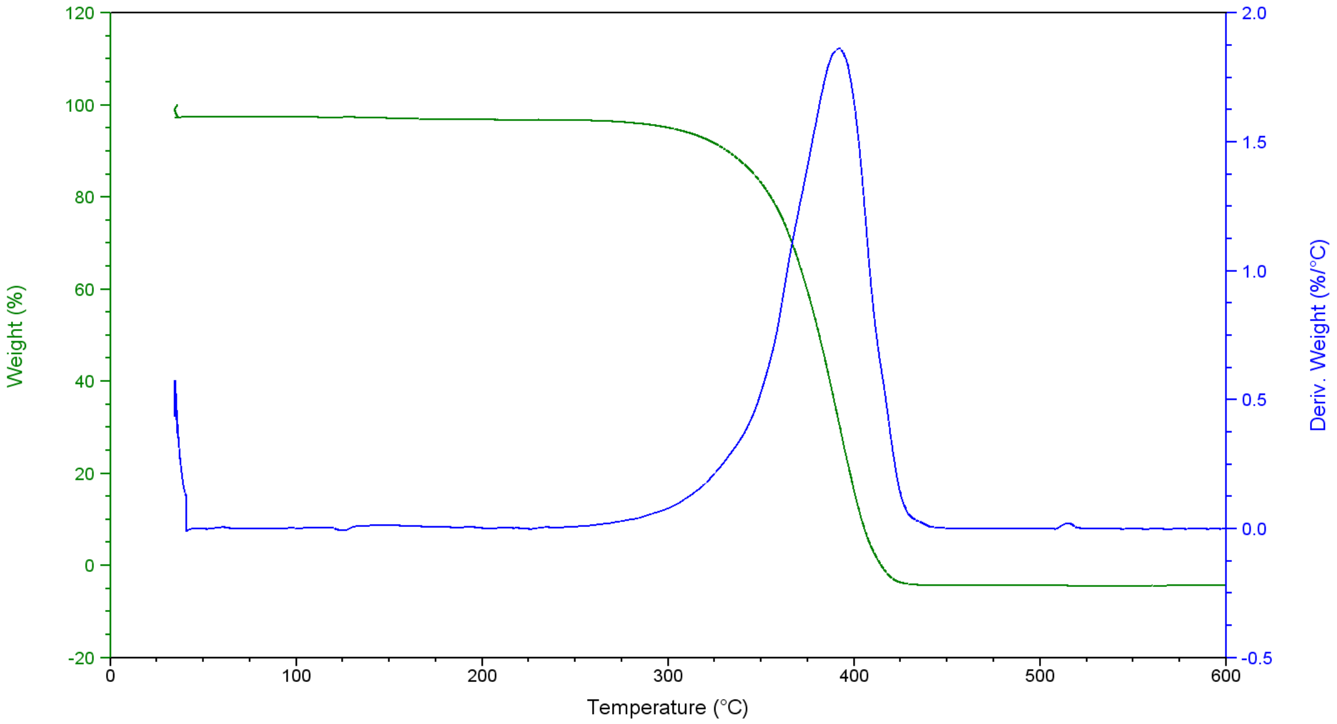

Figure S39. TGA thermogram of PS from run 5 of **Table 1**.

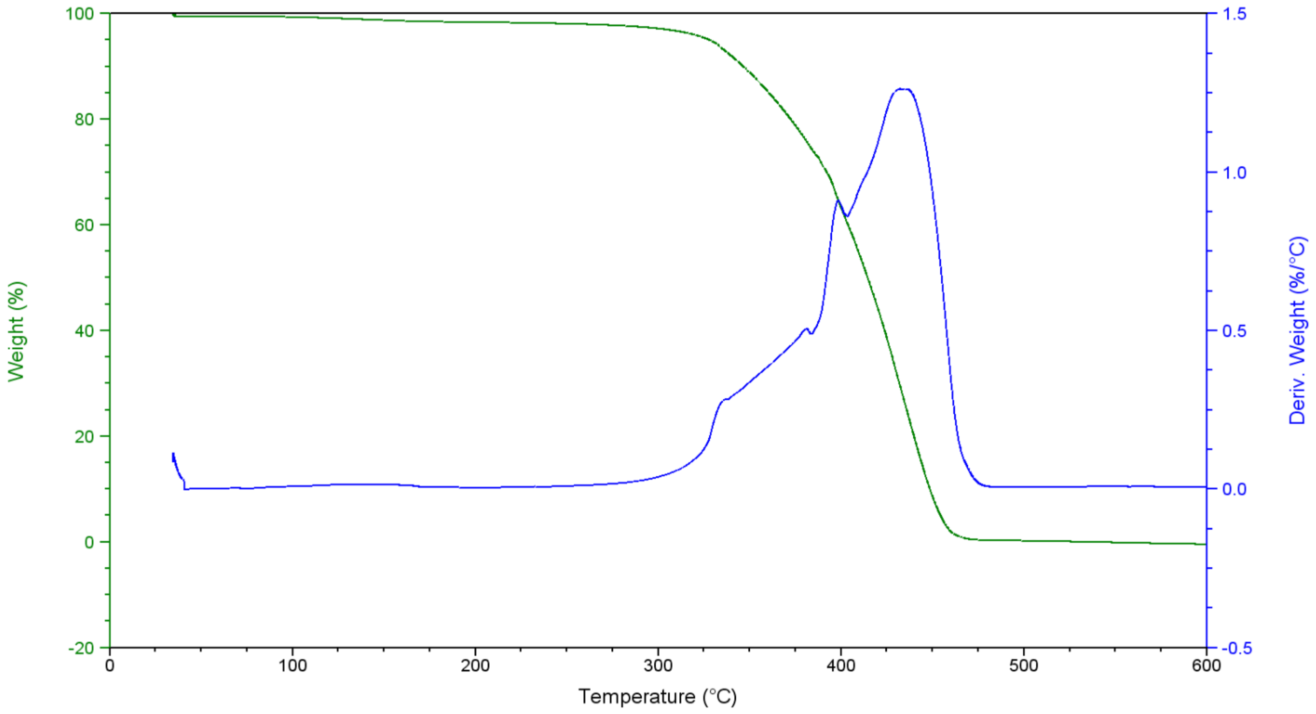

Figure S40. TGA thermogram of PI from run 8 of **Table 1**.

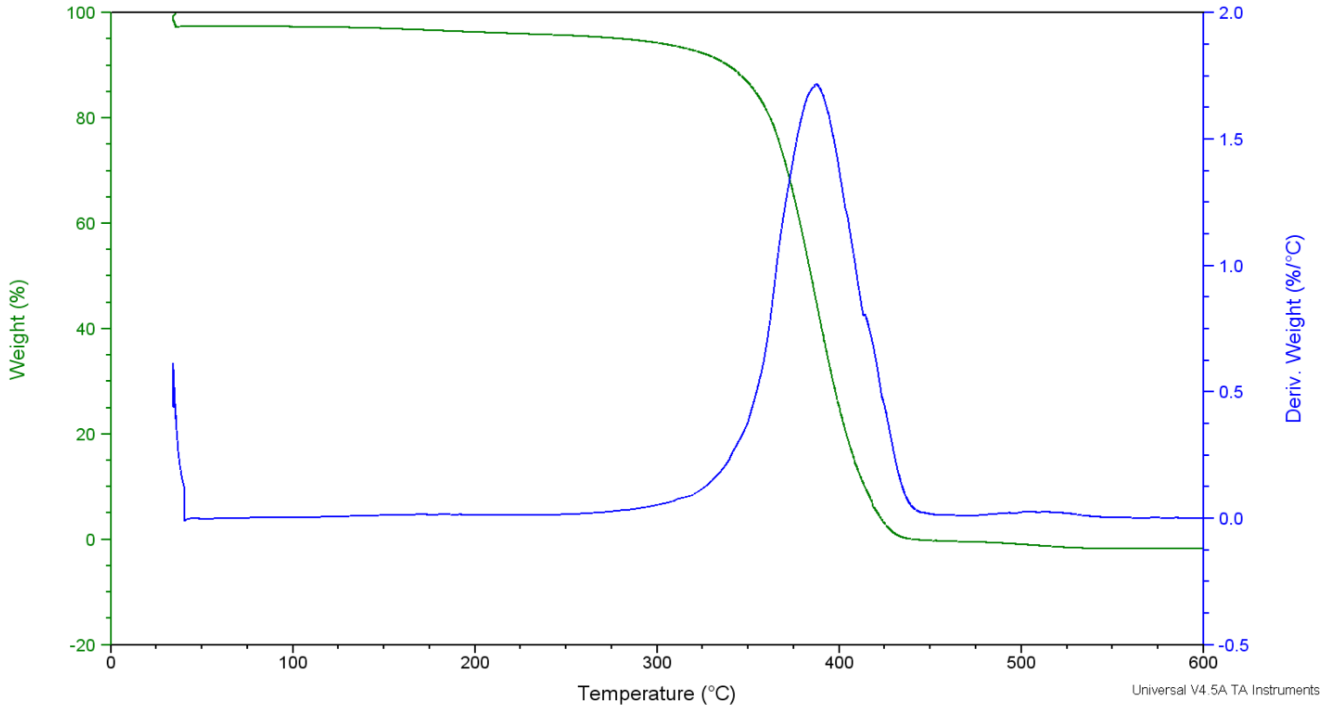

Figure S41. TGA thermogram of PMS from run 12 of **Table 2**.

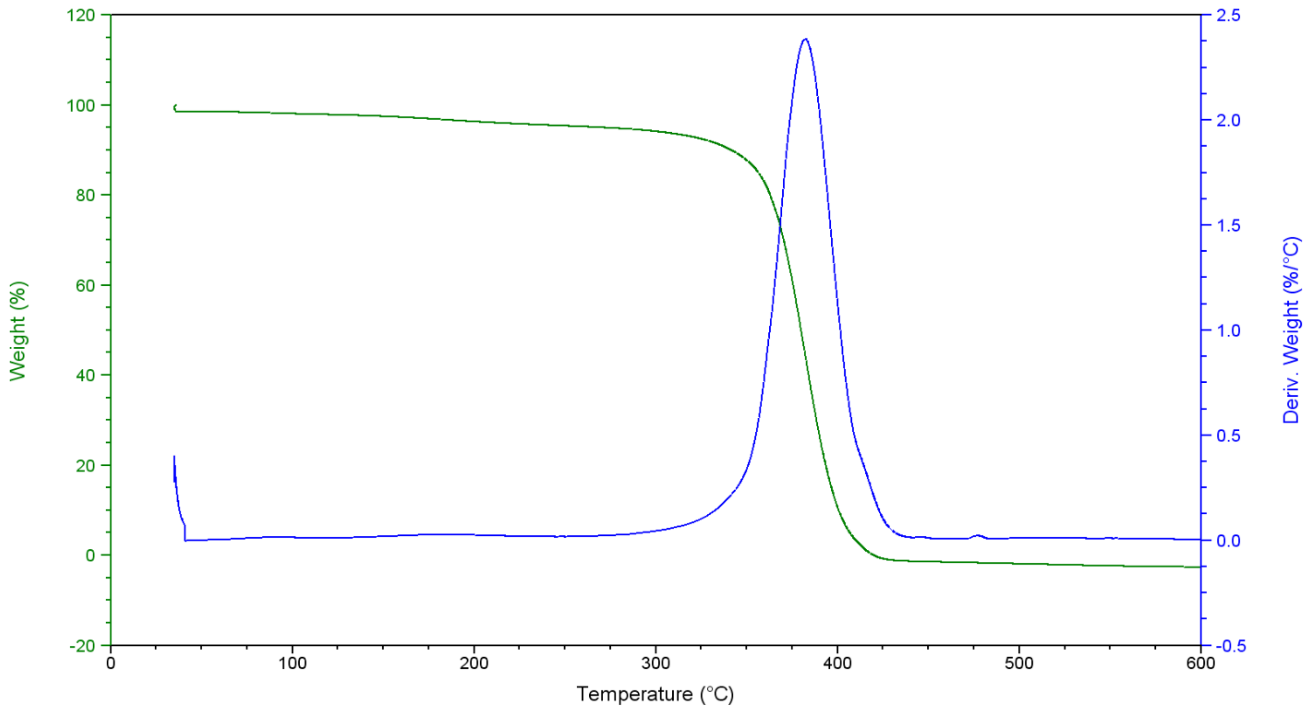

Figure S42. TGA thermogram of PMS from run 14 of **Table 2**.

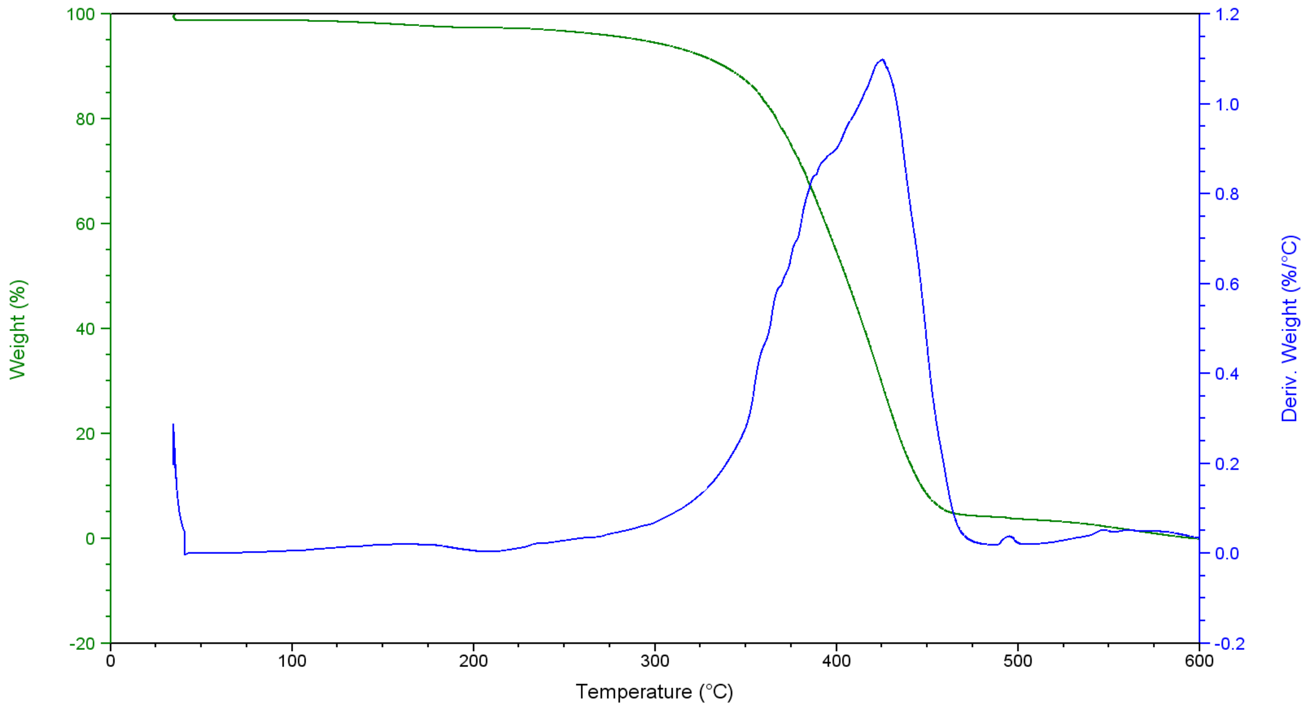

Figure S43. TGA thermogram of PMI from run 15 of **Table 2**.

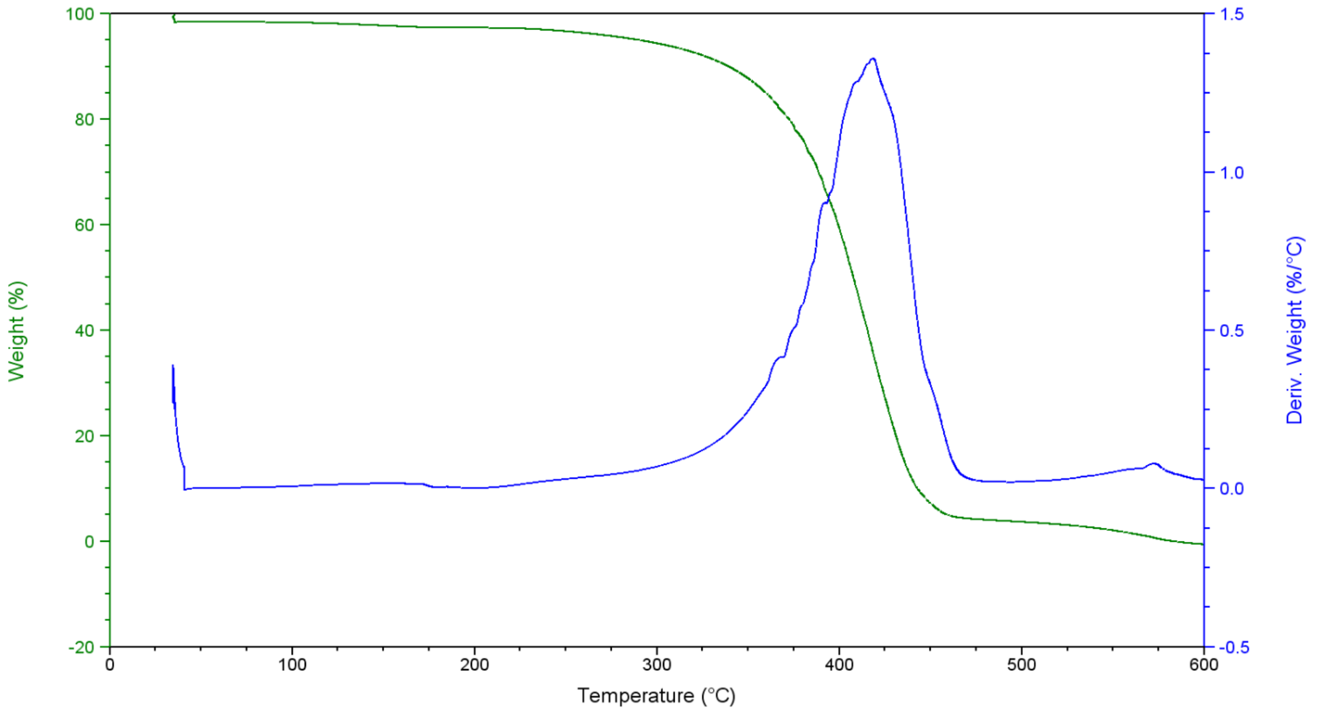

Figure S44. TGA thermogram of PMI from run 16 of **Table 2**.

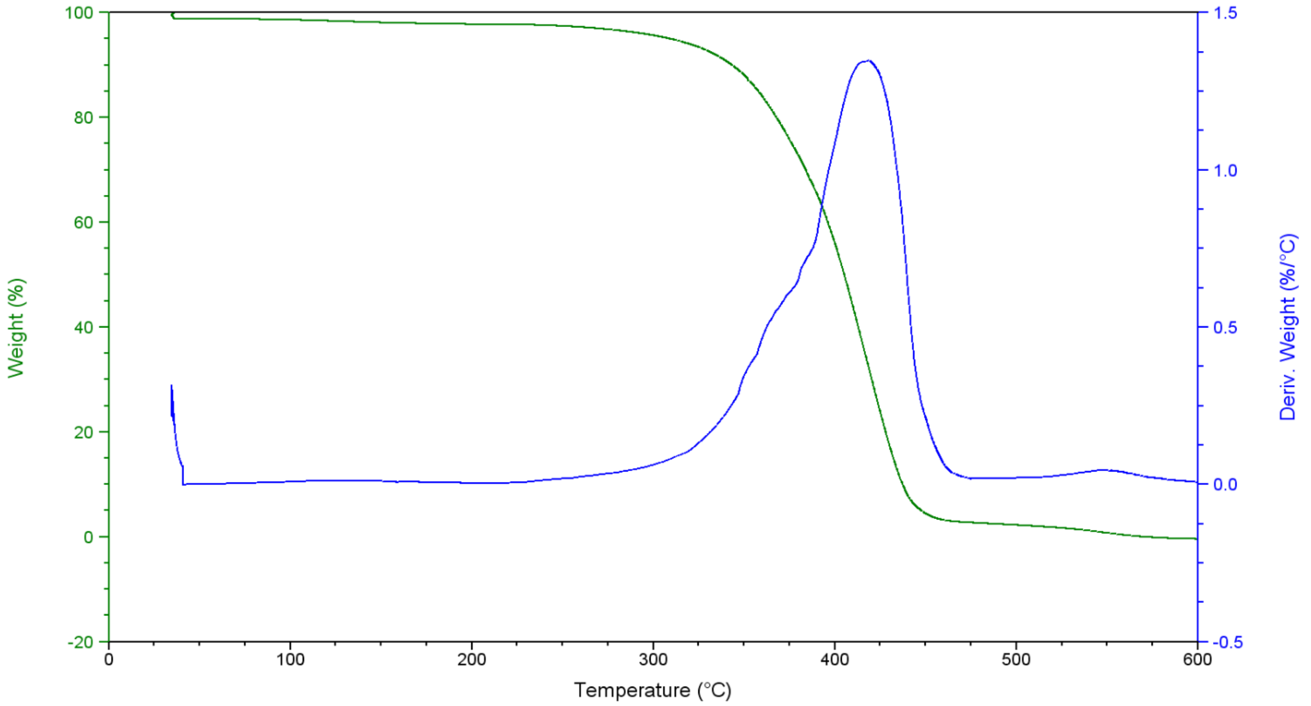

Figure S45. TGA thermogram of PMI from run 17 of **Table 2**.

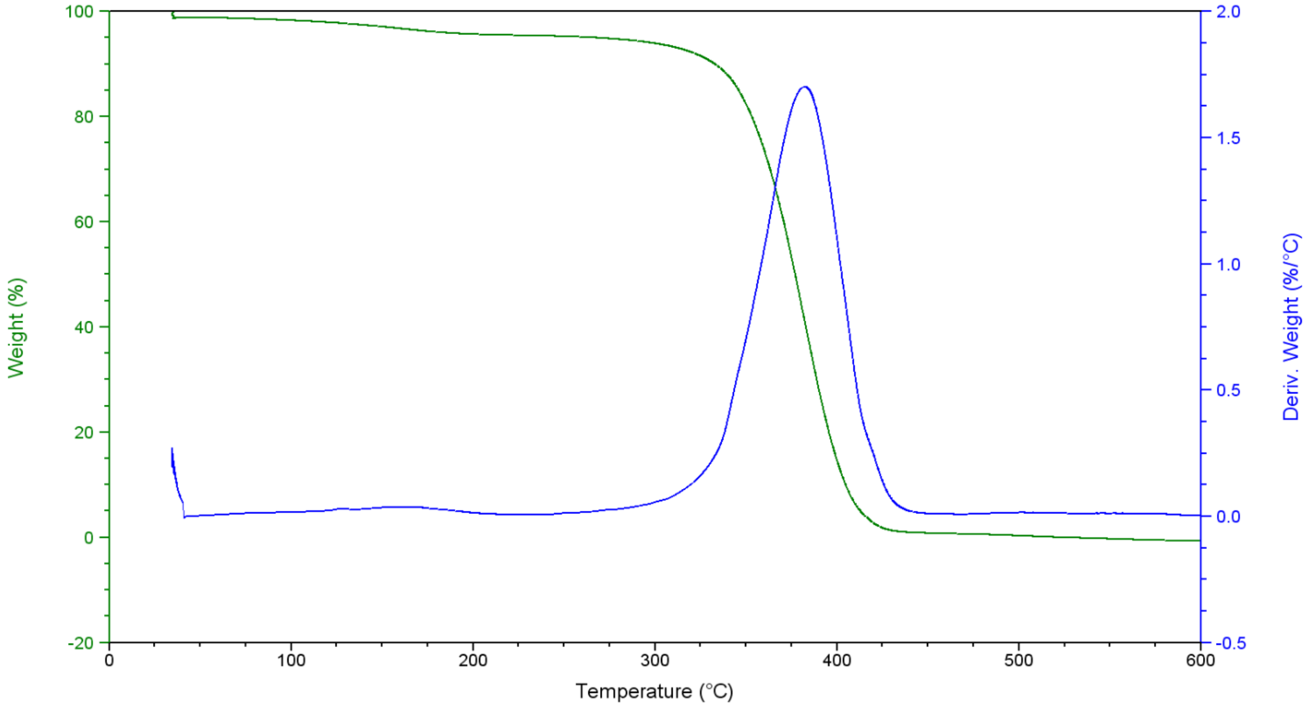

Figure S46. TGA thermogram of PMSI from run 20 of **Table 2**.

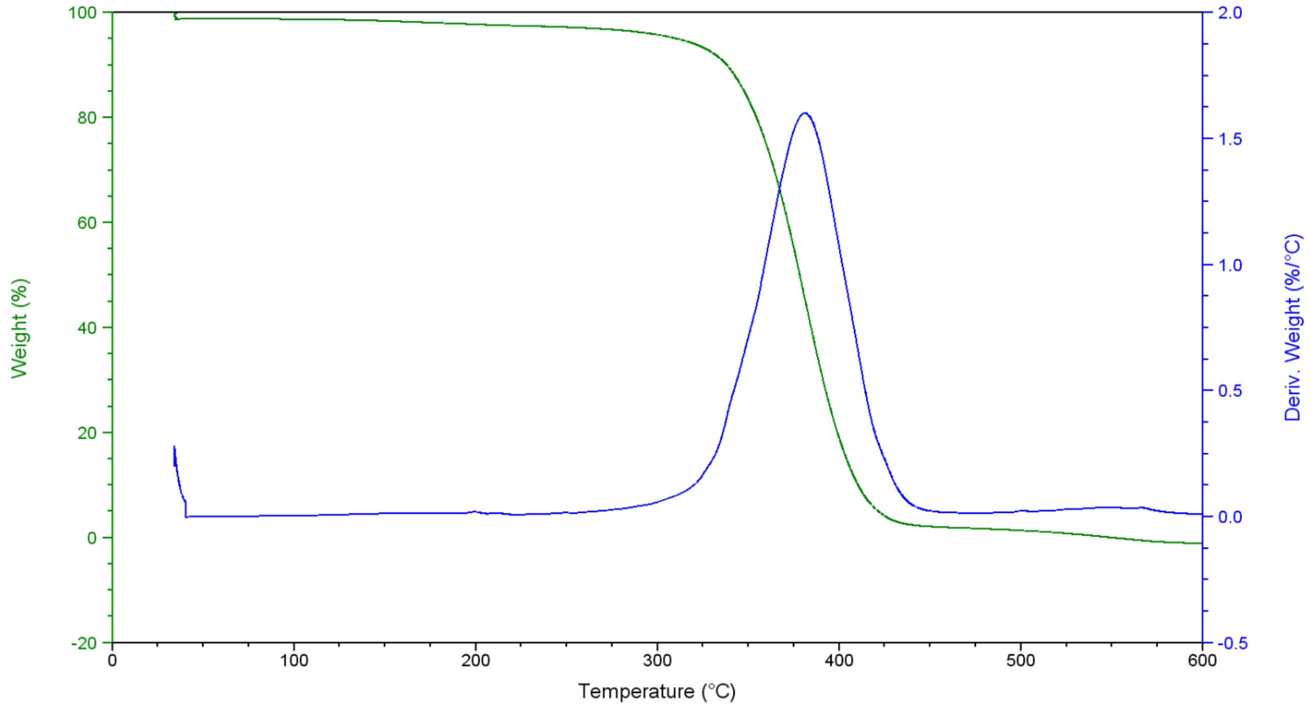

Figure S47. TGA thermogram of diblock PMS from run 21 of **Table 2**.

6. GPC Analysis

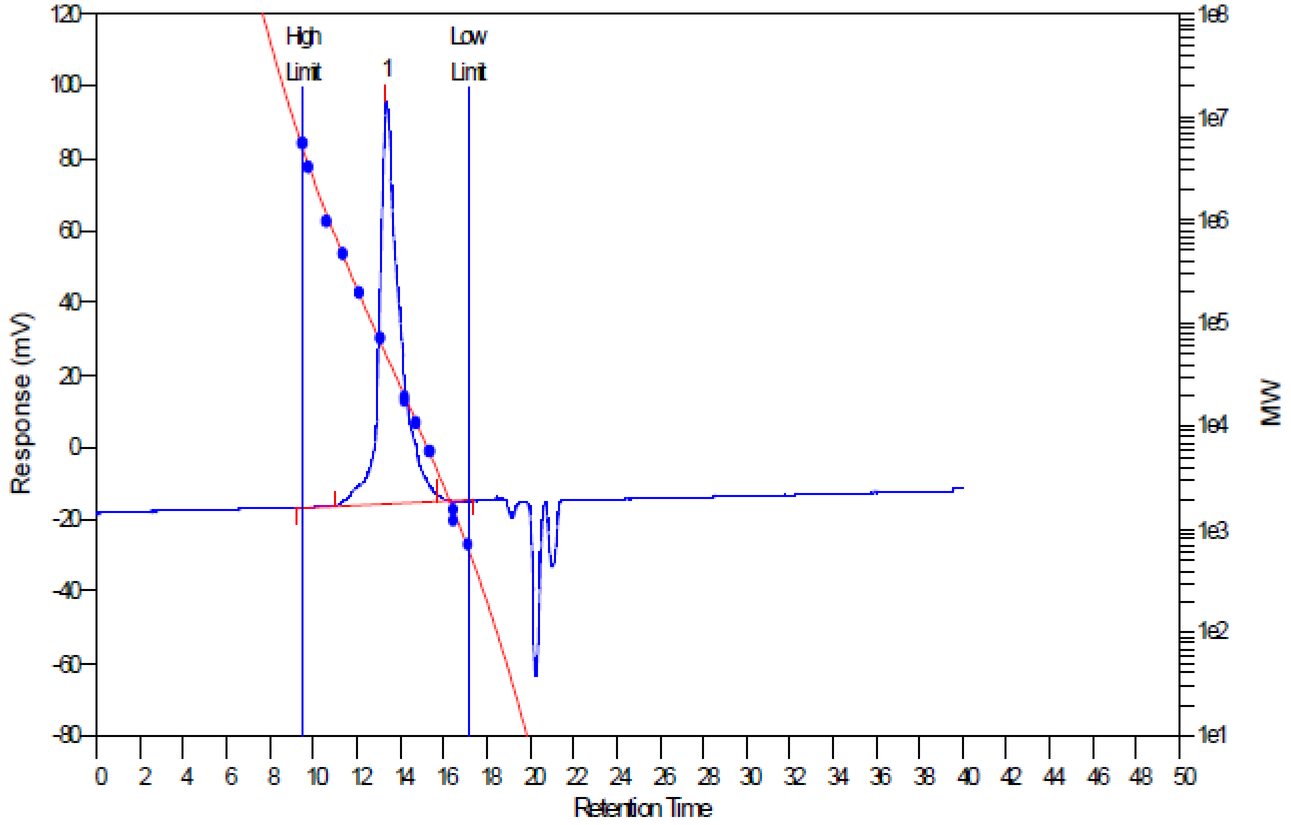

Figure S48. GPC curve of PM from run 1 of **Table 1.**

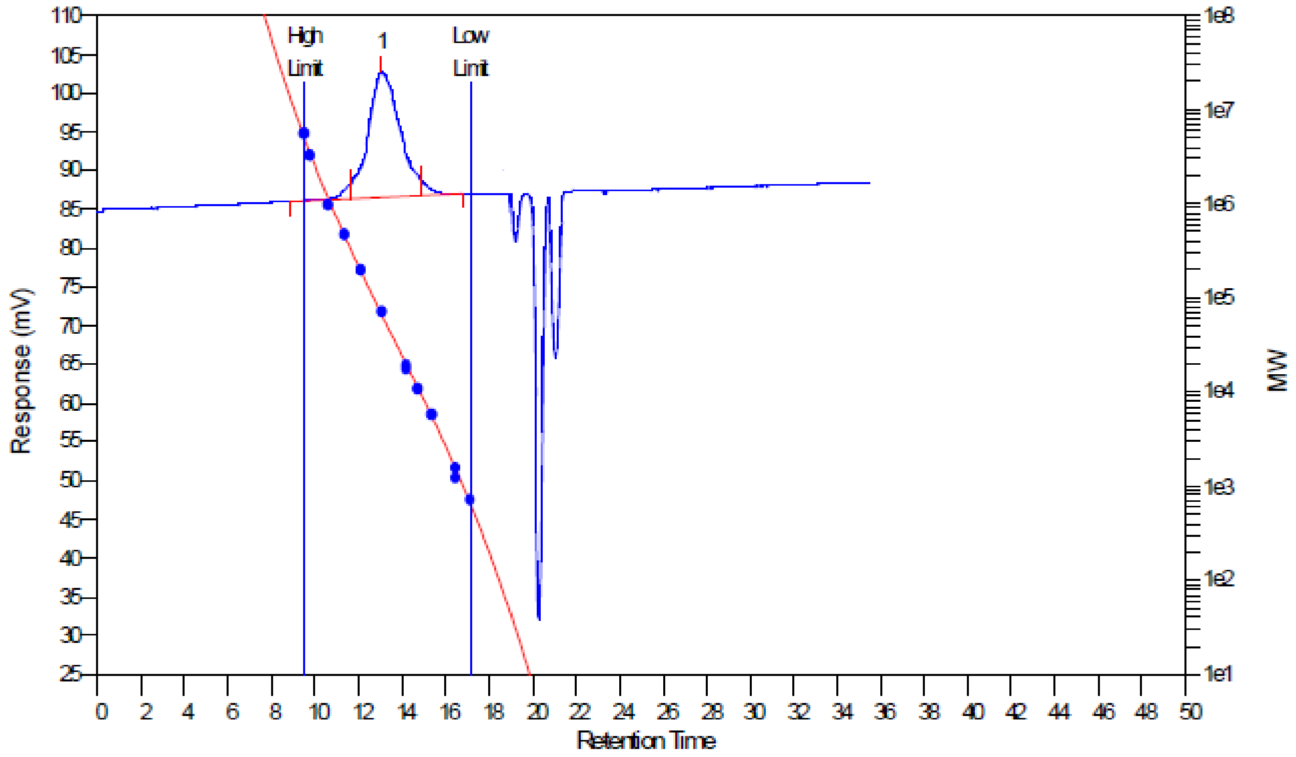

Figure S49. GPC curve of PM from run 2 of **Table 1.**

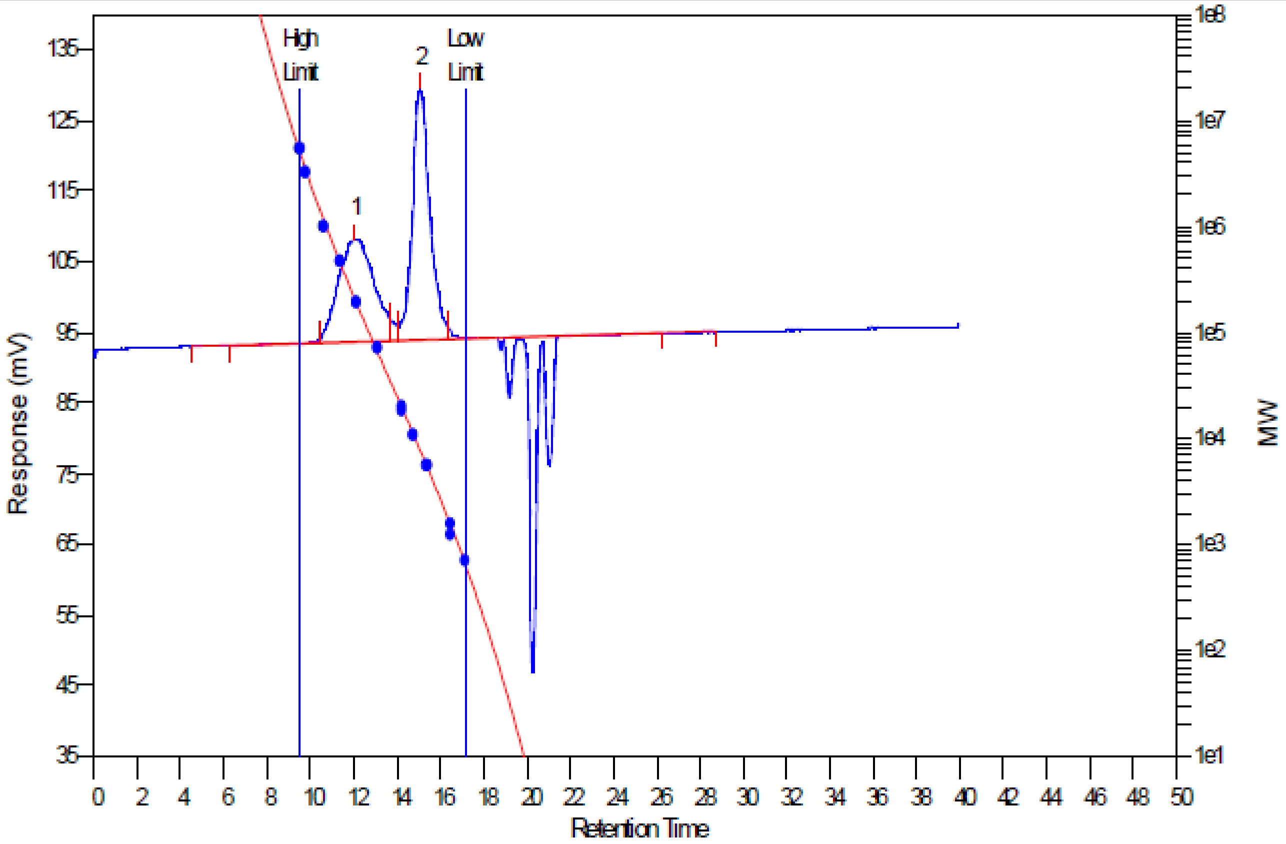

Figure S50. GPC curve of PM from run 3 of **Table 1.**

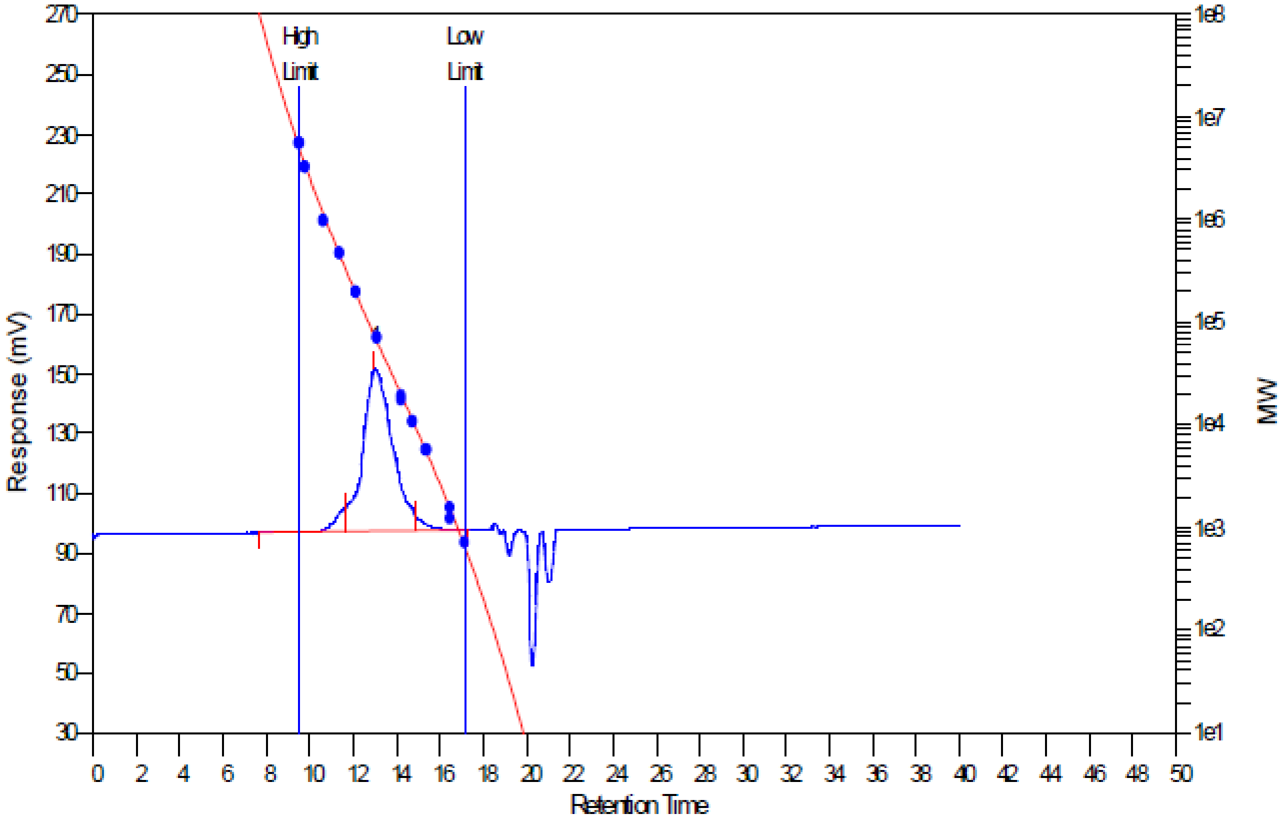

Figure S51. GPC curve of PM from run 4 of **Table 1.**

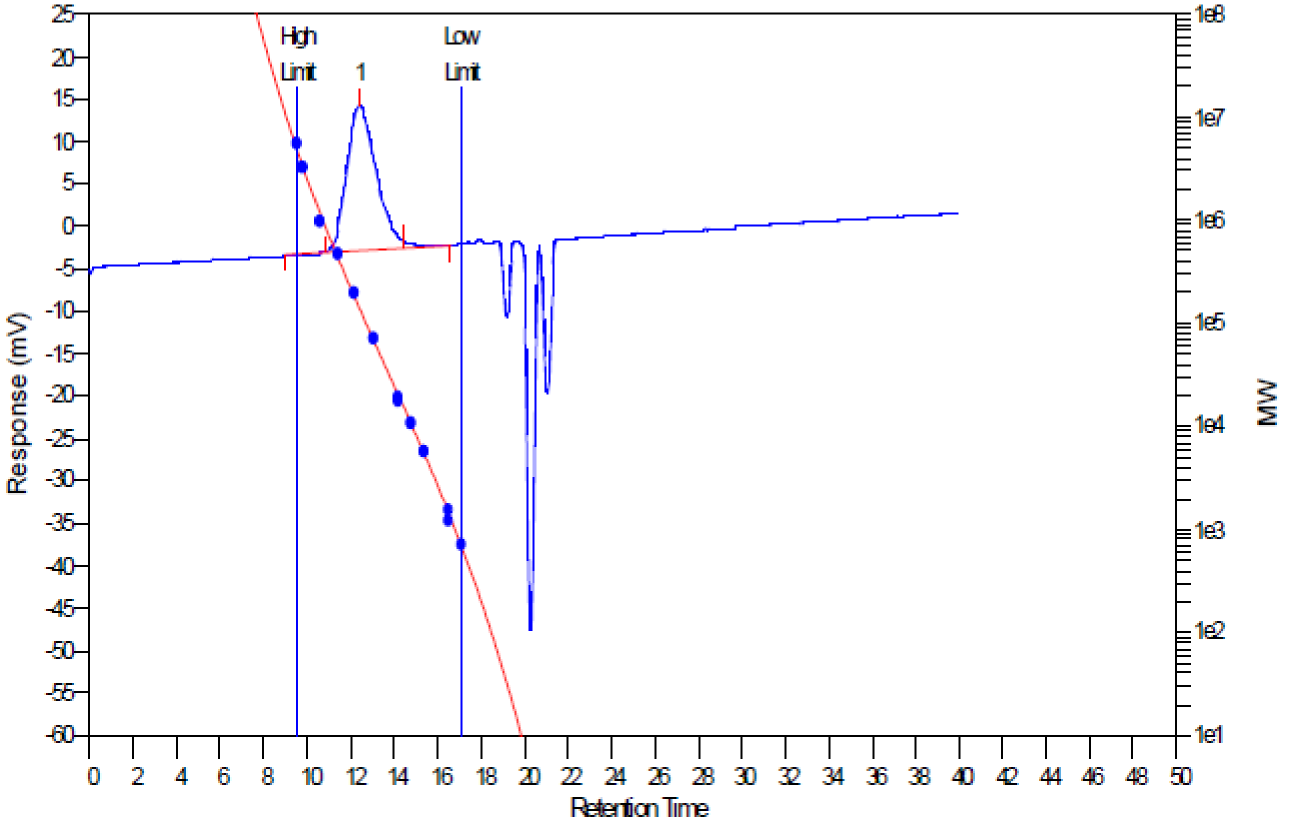

Figure S52. GPC curve of PS from run 5 of **Table 1.**

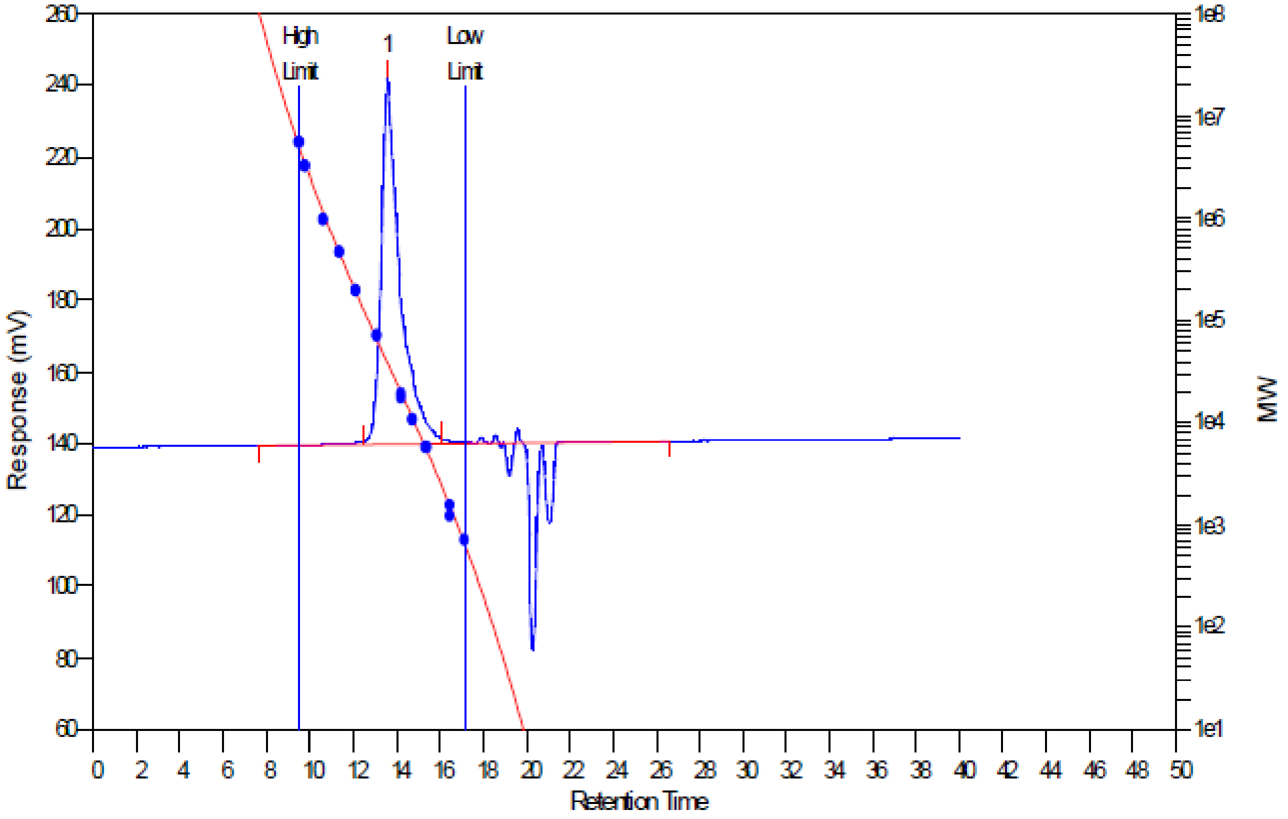

Figure S53. GPC curve of PI from run 8 of **Table 1.**

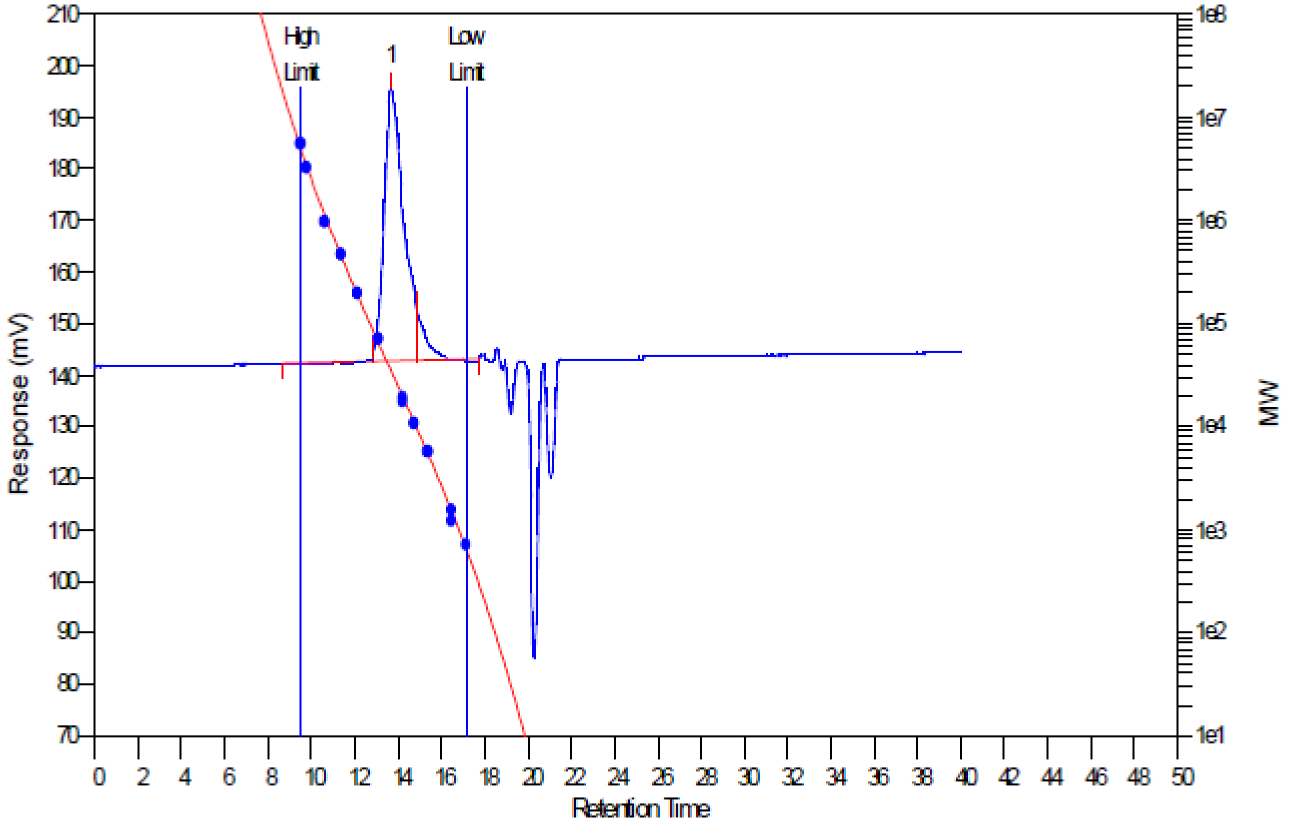

Figure S54. GPC curve of PI from run 9 of **Table 1.**

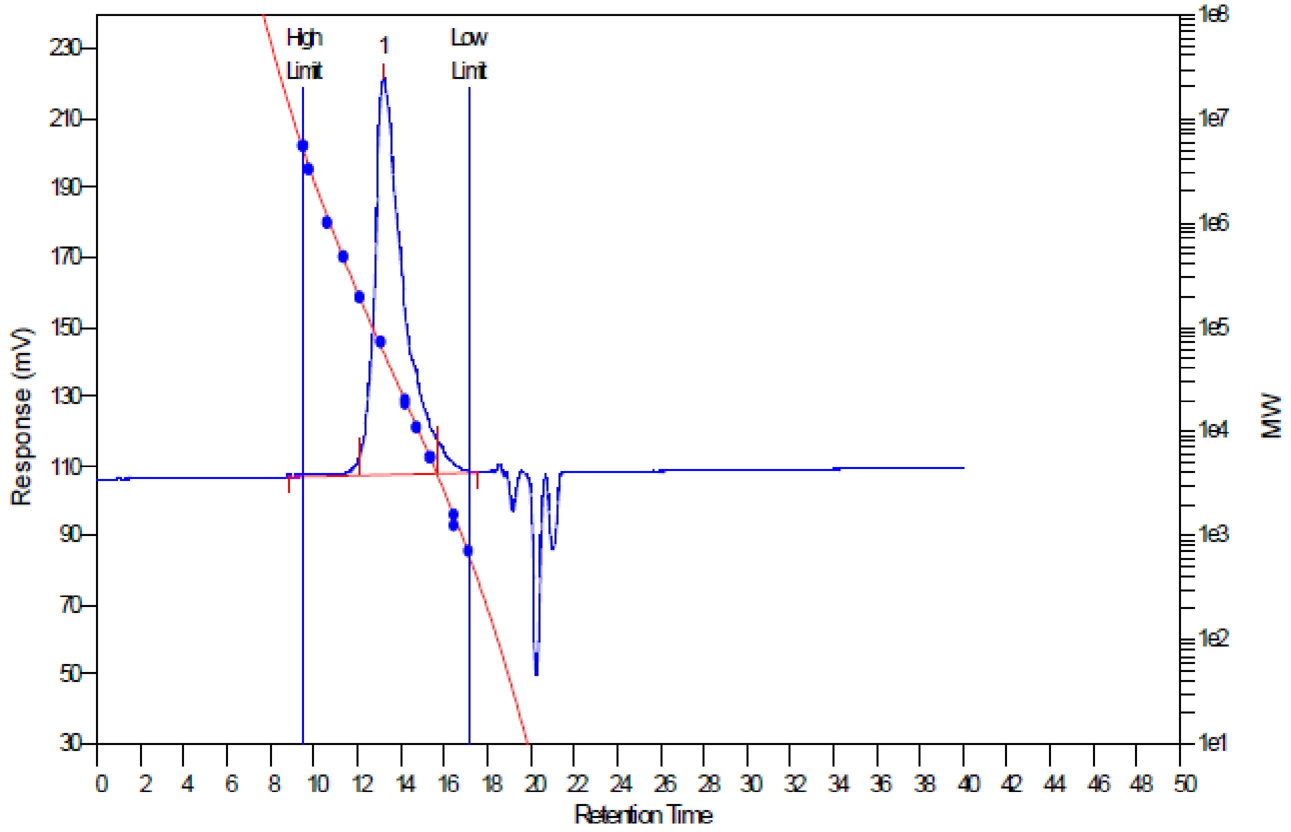

Figure S55. GPC curve of PMS from run 10 of **Table 2.**

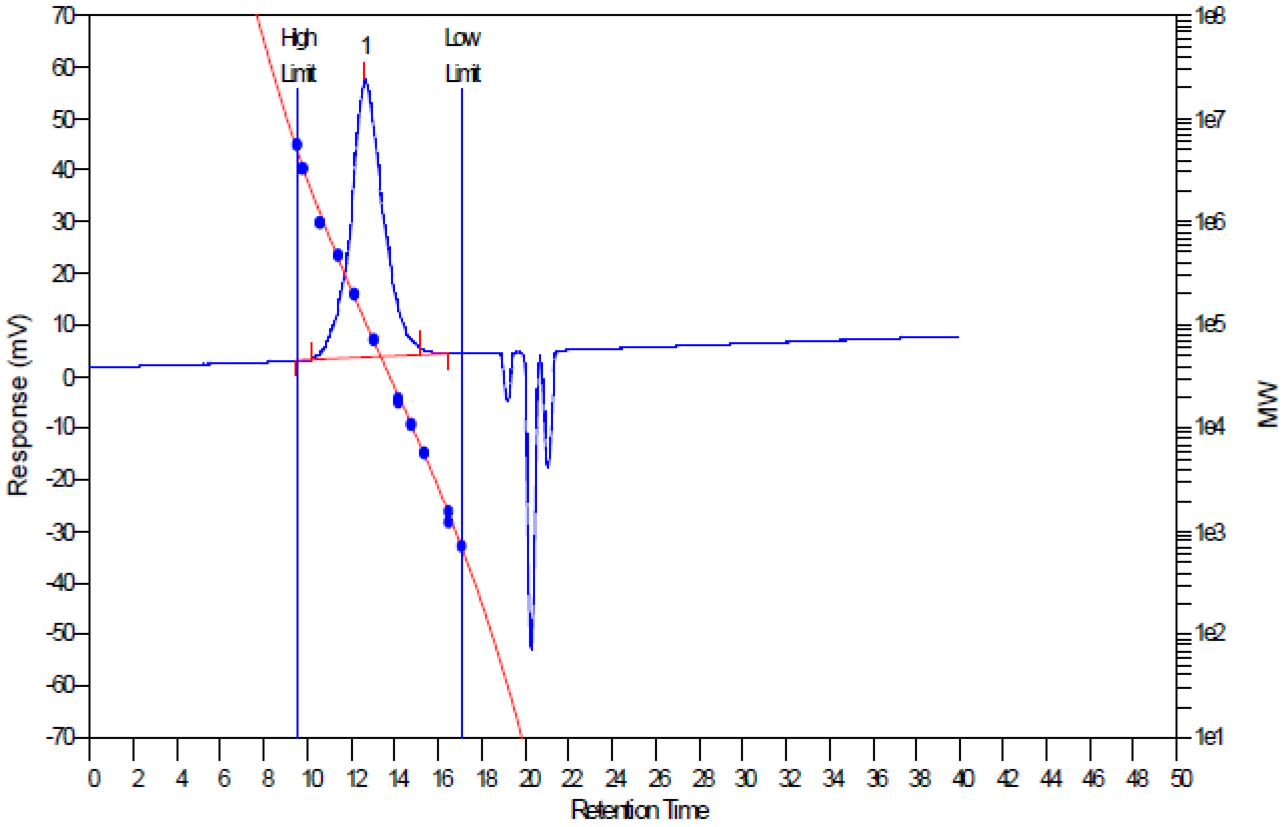

Figure S56. GPC curve of PMS from run 12 of **Table 2.**

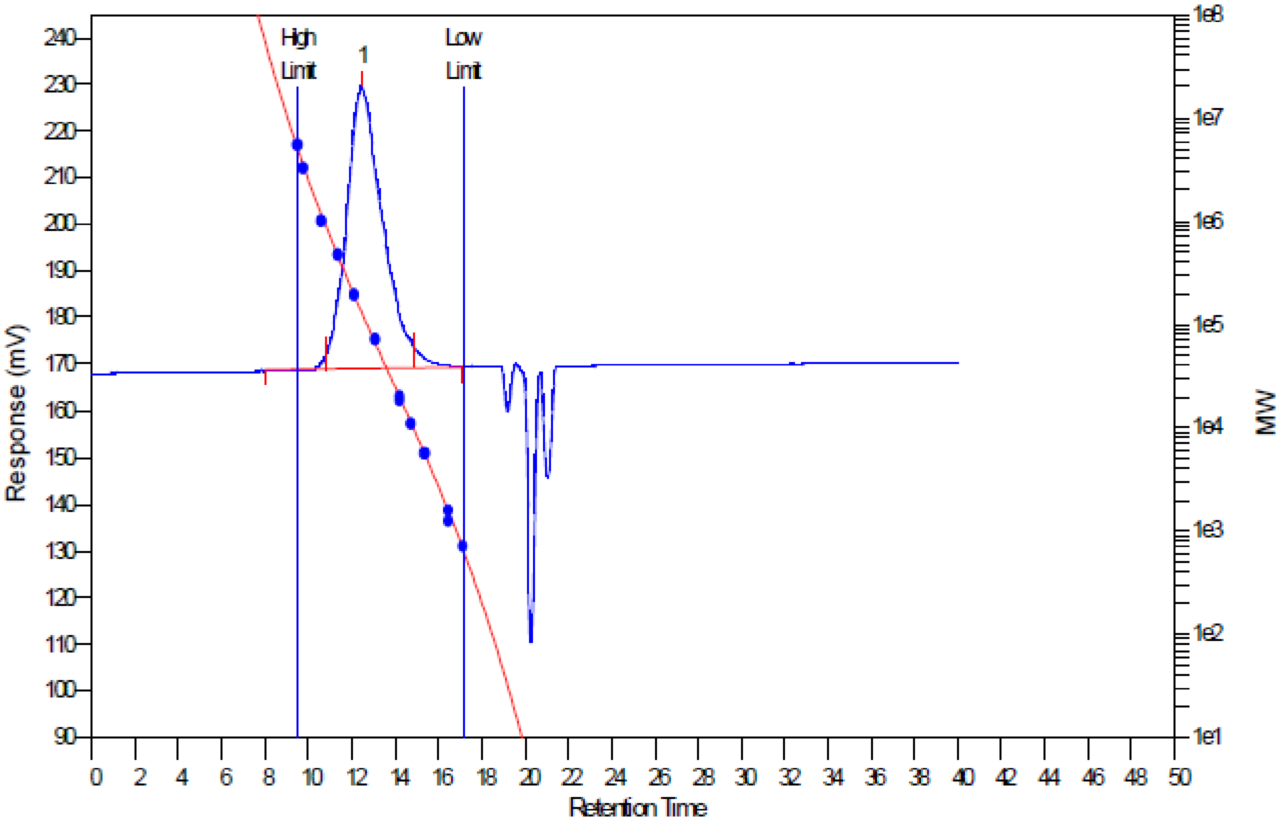

Figure S57. GPC curve of PMS from run 13 of **Table 2.**

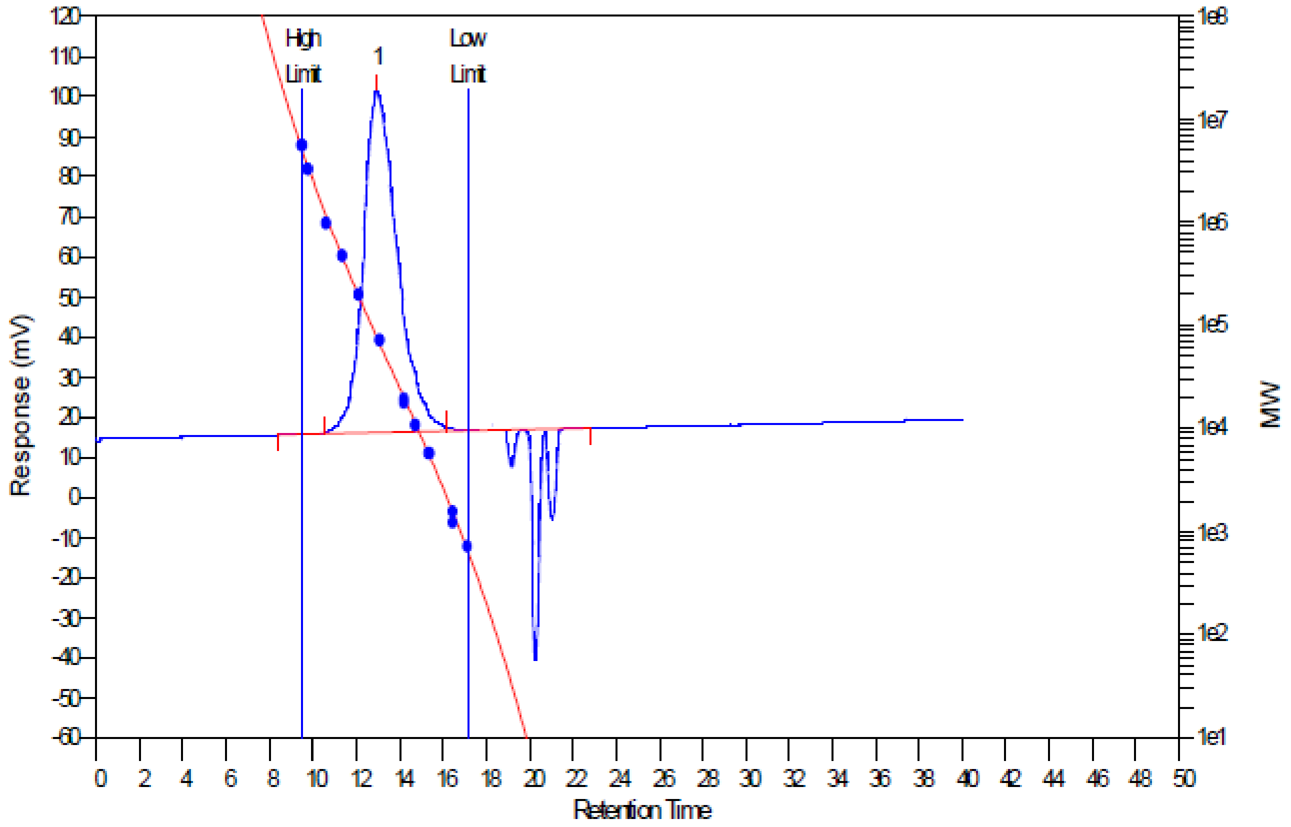

Figure S58. GPC curve of PMI from run 16 of **Table 2.**

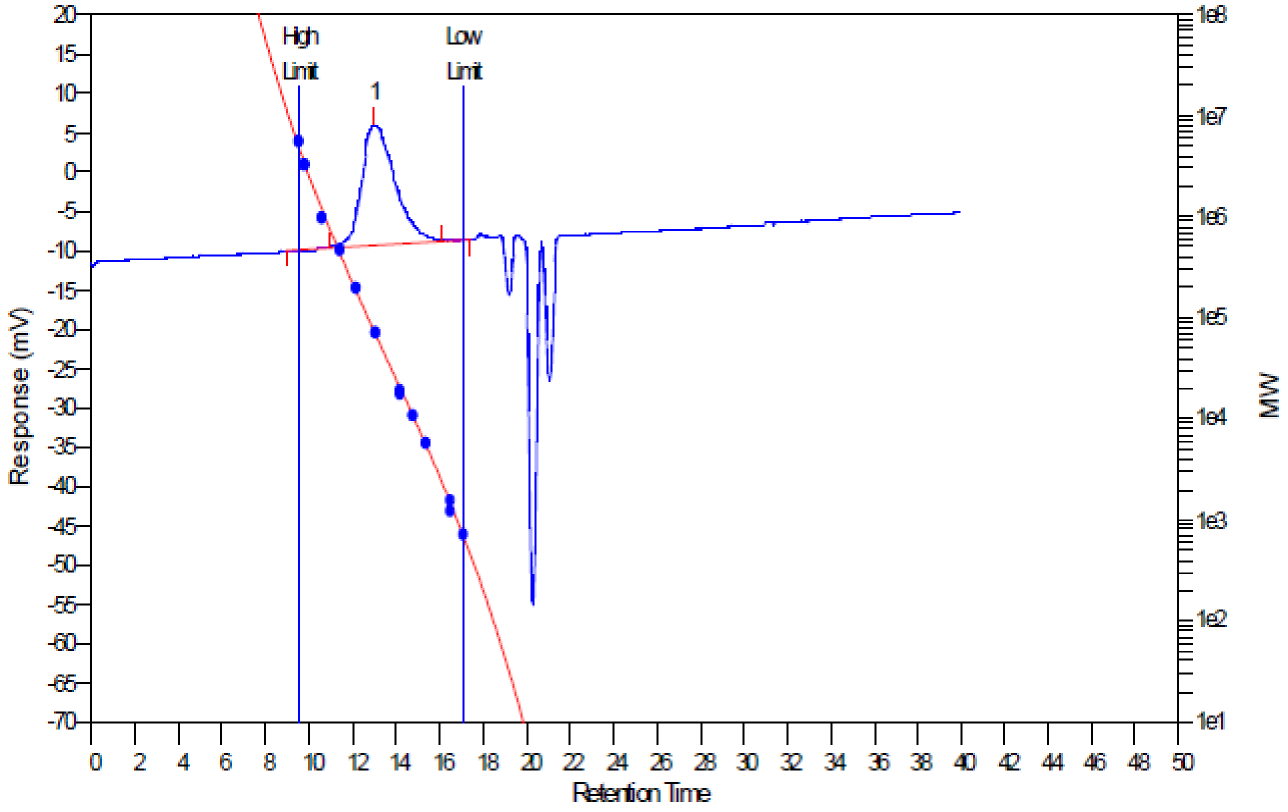

Figure S59. GPC curve of PMI from run 17 of **Table 2.**

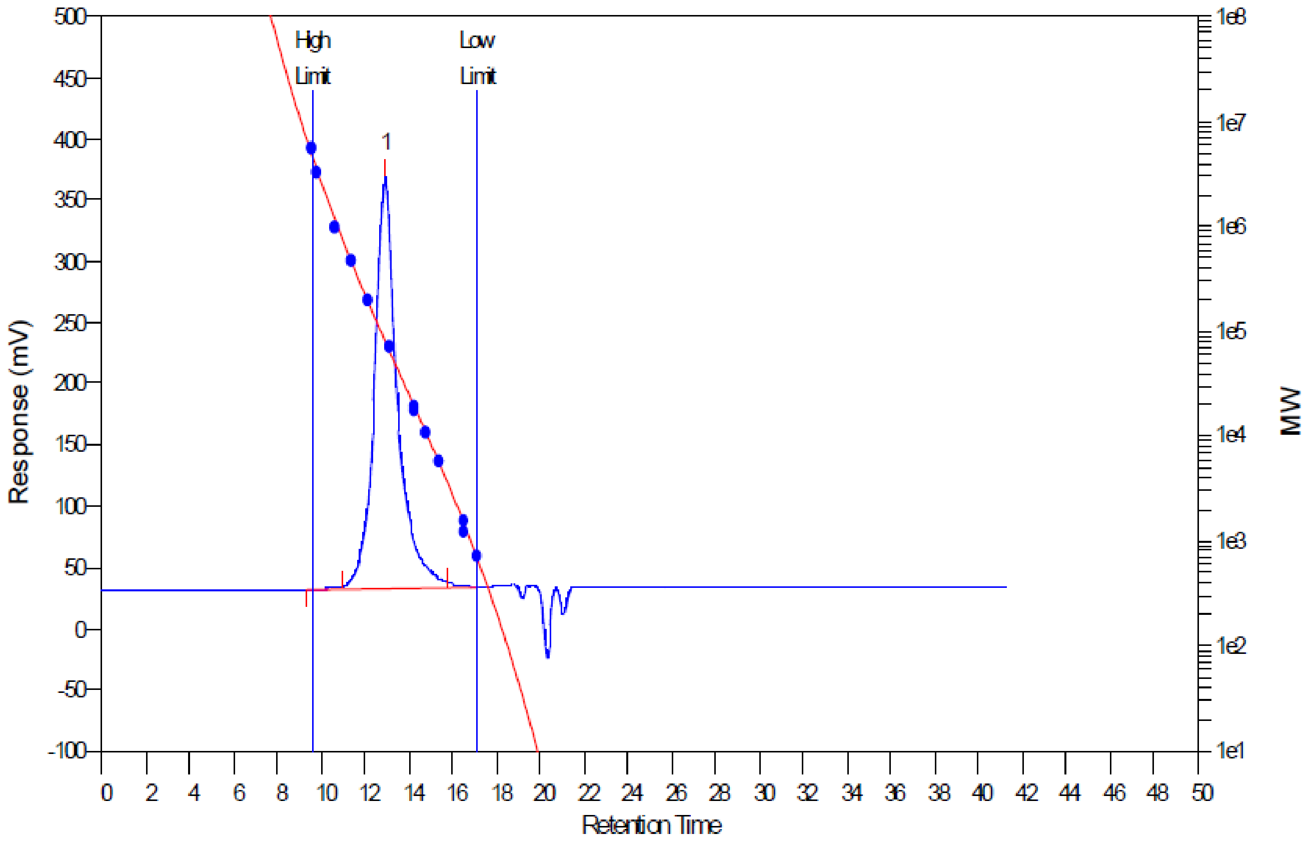

Figure S60. GPC curve of PMSI from run 20 of **Table 2.**

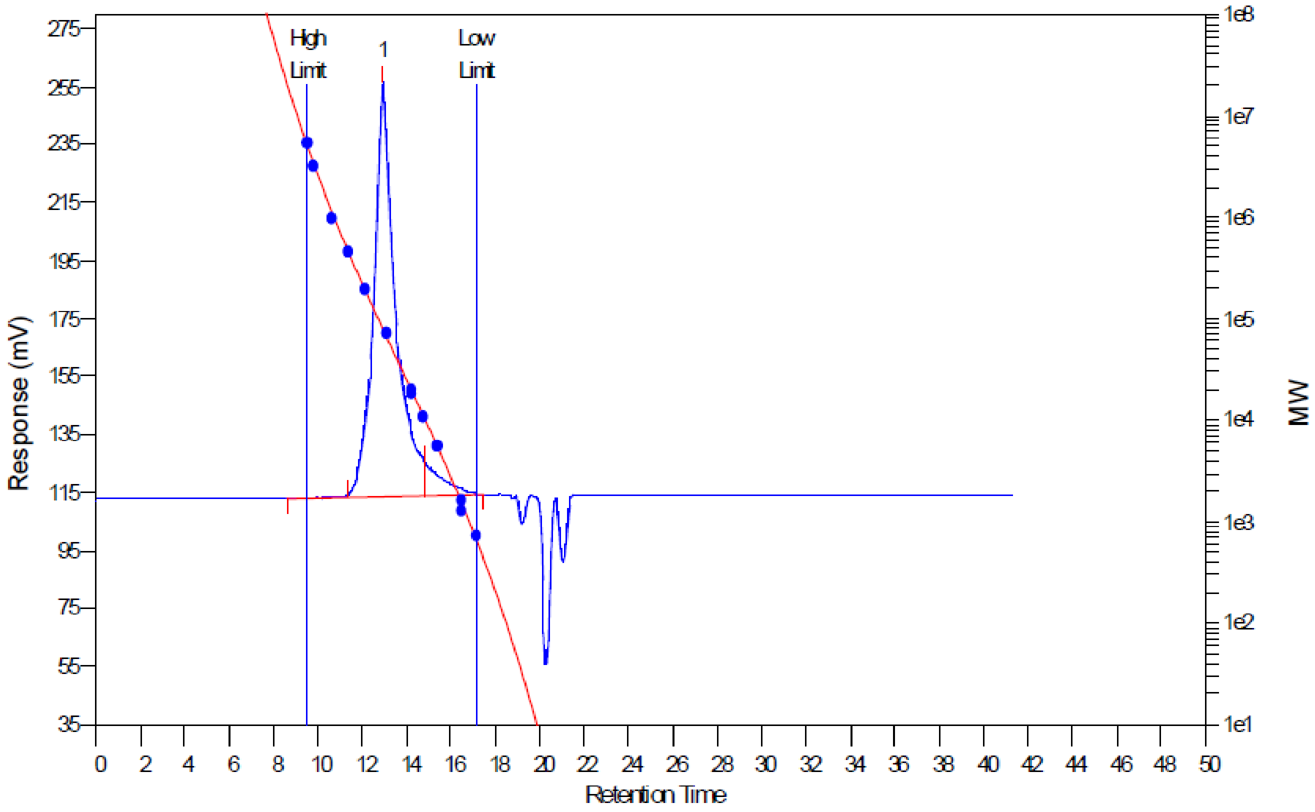

Figure S61. GPC curve of diblock PMS from run 21 of **Table 2.**

7. CAM Analysis

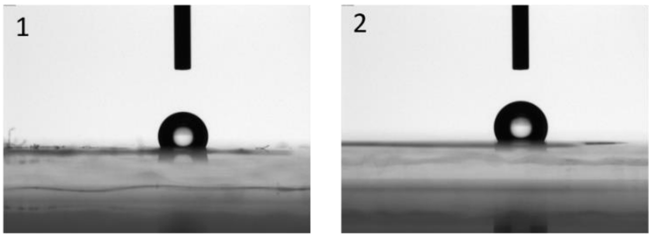

The CAM profiles of PI From run 7 Table 1

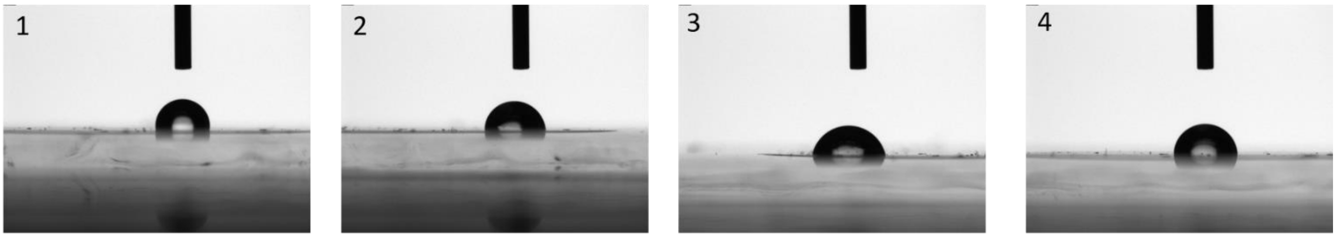

The CAM profiles of PMS From run 14 Table 2

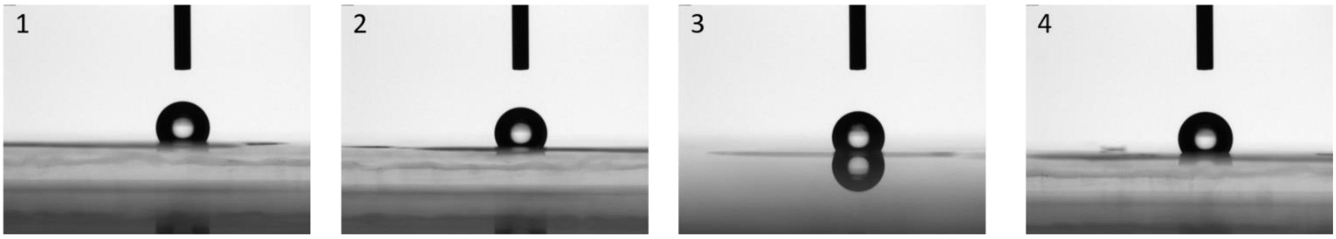

The CAM profiles of PMI From run 17 Table 2

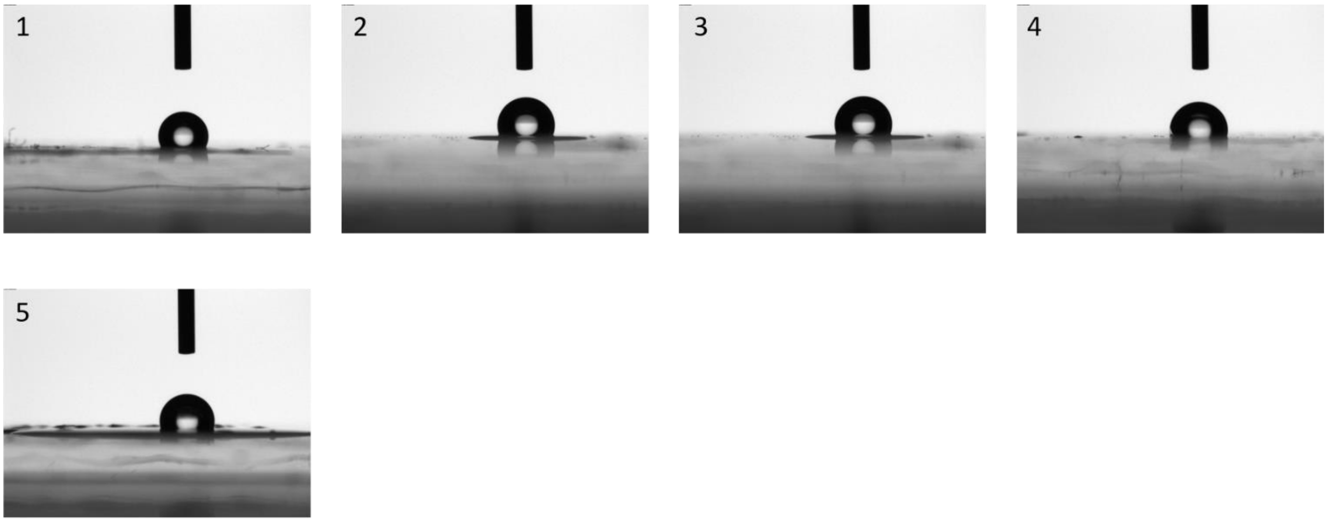

The CAM profiles of PMI From run 19 Table 2

Table T7 – Contact angles for tested samples

Sample	ϴ left	ϴ right	Contact angle
Run 7	114,5	115,6	115,05
"	116,1	112,4	114,25
**Run7**			**114,85**
Run 14	84,3	84,7	84,5
"	84,1	92,1	88,1
"	77,1	77,8	77,45
"	82,9	86,6	84,75
**Run 14**			**83,7**
Run 17	125,6	125,8	125,7
"	121	120,2	120,6
"	121,4	120,7	121,05
"	120,8	121,3	121,05
**Run 17**			**122,1**
Run 19	118,9	120,1	119,5
"	113,7	109,2	111,45
"	108	102	105
"	101,2	110,5	105,85
"	110,8	106,7	108,75
**Run 19**			**110,11**

8. SEM Analysis

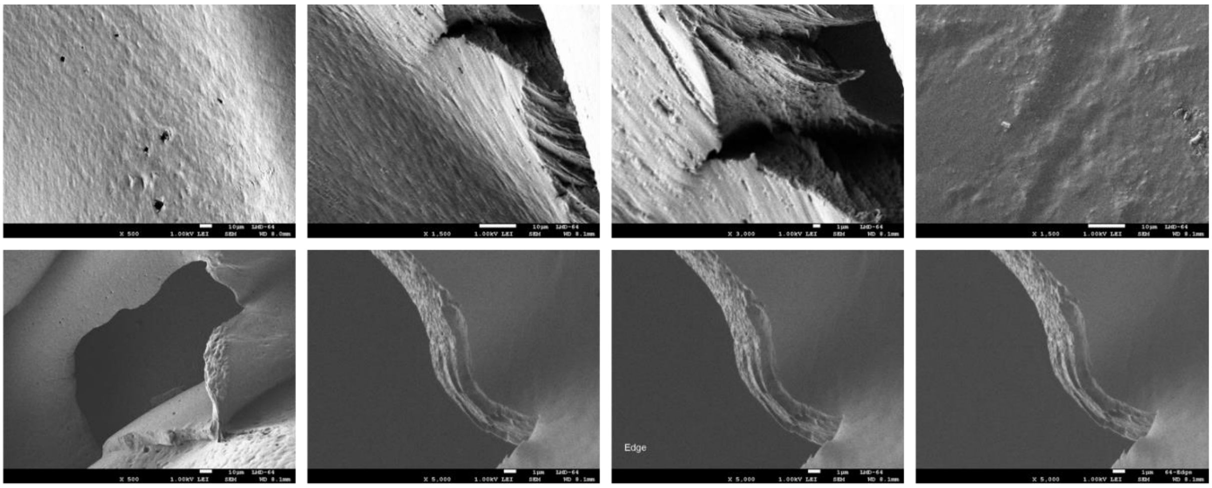

SEM image of PMS from run 14 Table 2

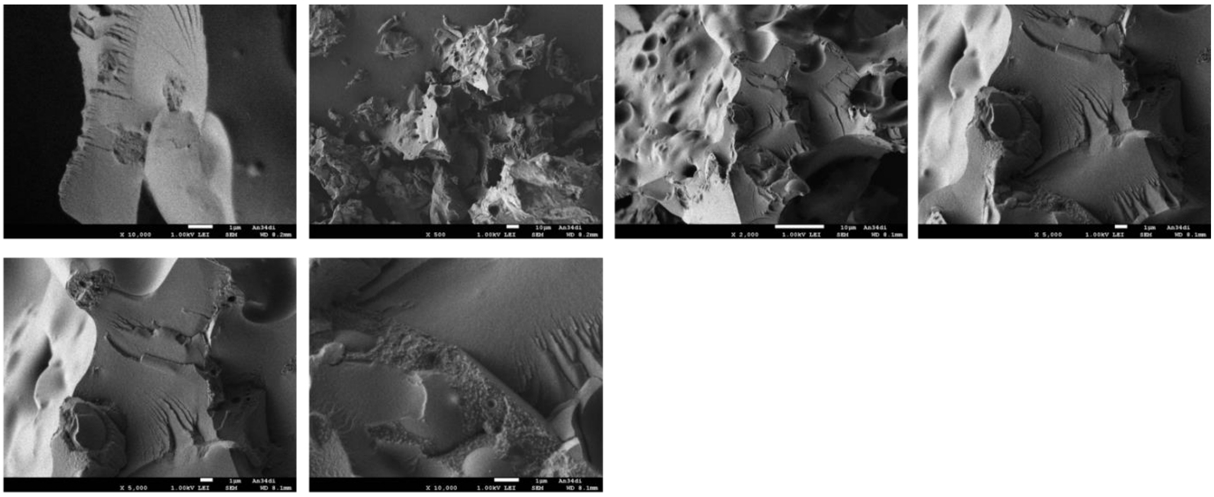

SEM image of PMS from run 21 Table 2

9. Kinetic studies

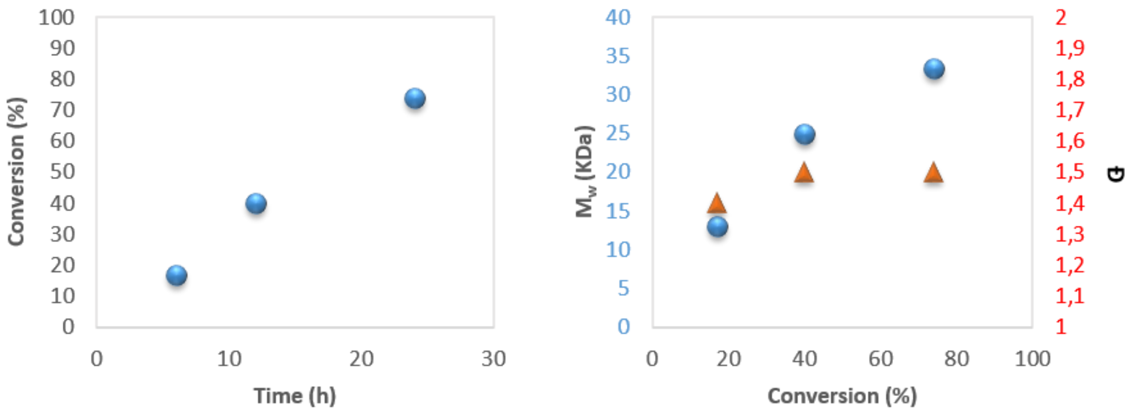

Figure S61. Conversion vs. time plot for isoprene polymerization (on left); plots of M_W (and Đ) vs conversion (on right)

Same experimental conditions of run 7 of **Table 1** (except for time)

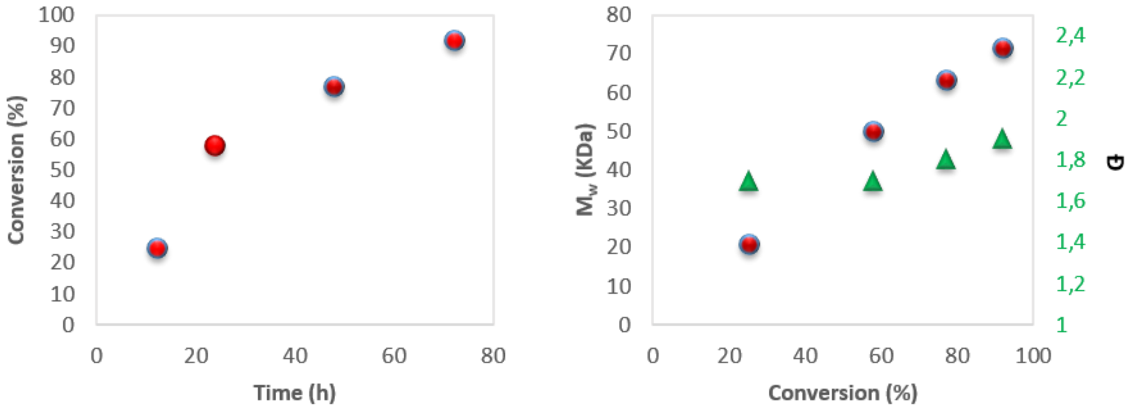

Figure S62. Conversion vs. time plot for myrcene polymerization (on left); plots of M_W (and Đ) vs conversion (on right)

Same experimental conditions of run 1 of **Table 1** (except for time)

10. References

1 M. I. Hulnik, I. V. Vasilenko, A. V. Radchenko, F. Peruch, F. Ganachaud and S. V. Kostjuk, Polym. Chem., 2018, 9, 5690–5700.
2 M. Naddeo, A. Buonerba, E. Luciano, A. Grassi, A. Proto and C. Capacchione, Polymer, 2017, 131, 151–159.
3 X. Jia, X. Zhang and D. Gong, J. Polym. Sci. Part A: Polym. Chem., 2018, 56, 2286–2293.
4 W. Li, J. Zhao, X. Zhang and D. Gong, Ind. Eng. Chem. Res., 2019, 58, 2792–2800.
5 P. Sarkar and A. K. Bhowmick, ACS Sustainable Chem. Eng., 2016, 4, 5462–5474.
6 C. Zhou, Z. Wei, X. Lei and Y. Li, RSC Adv., 2016, 6, 63508–63514.

11. Author Contributions

Conceptualization, M.W., C.C., and D.H.L.; Methodology, M.W. and D.H.L.; Investigation, D.H.L., M.K.; Resources, M.W., C.C.; Data Curation, D.H.L.; Writing—Original Draft Preparation, D.H.L.; Writing—Review and Editing, M.W., C.C., and D.H.L.; Funding Acquisition, M.W., C.C.
